# Stereoselective Synthesis of Densely Substituted Pyrrolidines via a [3 + 2] Cycloaddition Reaction between Chiral *N*-*tert*-Butanesulfinylazadienes and Azomethine Ylides

**DOI:** 10.1021/acs.orglett.3c02572

**Published:** 2023-10-04

**Authors:** Ester Blanco-López, Francisco Foubelo, María de Gracia Retamosa, José M. Sansano

## Abstract

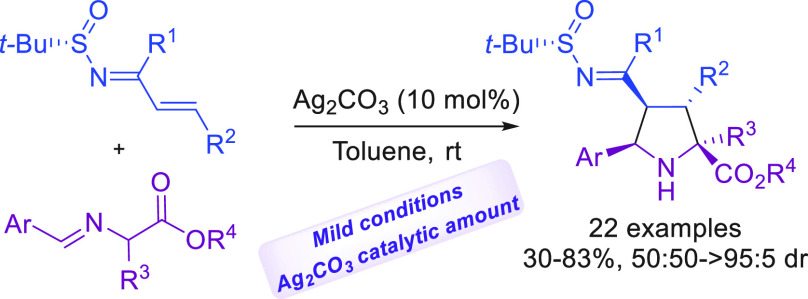

The *N*-*tert*-butanesulfinylimine group behaves as a suitable electron-withdrawing group in 1-azadienes, allowing the diastereoselective synthesis of densely substituted pyrrolidines by 1,3-dipolar cycloadditions (1,3-DCs) with azomethylene ylides. The use of Ag_2_CO_3_ as catalyst has allowed one to obtain a wide variety of proline derivatives with high regio- and diastereoselectivities. Subsequent efficient transformations provide valuable proline derivatives, some of which can be used as organocatalysts. The influence of the *N*-*tert*-butanesulfinyl group on the diastereoselectivity was studied by computational methods.

Diastereoselective and enantioselective 1,3-dipolar cycloadditions (1,3-DCs) are very interesting processes as up to four stereogenic centers can be generated simultaneously.^1^ In particular, azomethine ylides, which are often generated *in situ*, have been demonstrated to be useful intermediates for reaction with alkenes to yield pyrrolidines. The proline derivatives obtained in these transformations have many applications in organic synthesis such as organocatalysts,^2^ antitumor agents,^3^ and antivirals.^4^ The diastereoselective version allows chiral information to be introduced into the dipolar precursor or dipolarophile. In this context, our group has demonstrated the high diastereoselectivity of these processes using a chiral dipole precursor^5^ or a chiral dipolarophile^6^ despite the small size of the groups surrounding the stereogenic center. This strategy has also been used by the Viso group to synthesize chiral imidazolidines using nonracemic *p*-tolylsulfinimines and azomethine ylides, generated *in situ* from iminoesters and LDA (Scheme 1A).^7^ Moreover, they observed that the presence of Lewis acids promoted the formation of the cycloadducts through a highly diastereoselective process with opposite stereochemistry.

**Scheme 1:**
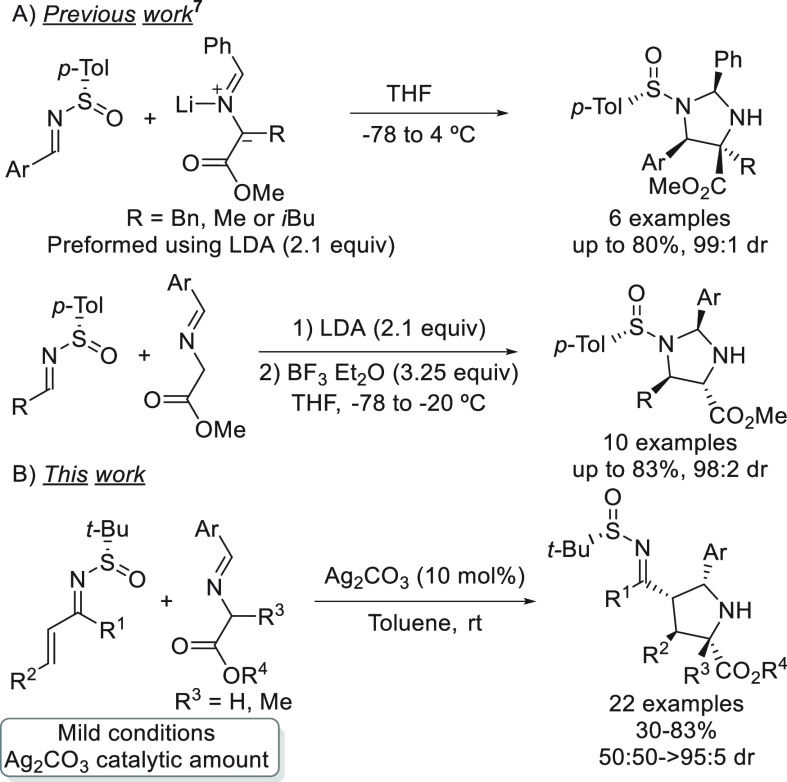
1,3-Dipolar Cycloadditions between Sulfinylimines and Azomethine Ylides

On the other hand, *tert*-butanesulfinyl imines are highly versatile chiral compounds that find extensive application as electrophiles in a wide range of reactions.^8^ The electron-withdrawing sulfinyl group present in these compounds significantly enhances the nucleophilic addition to the iminic carbon, resulting in a high diastereoisomeric excess.^9^ The accessibility of both enantiomers of *tert*-butanesulfinamide^10^ enables the synthesis of *tert*-butanesulfinyl imines^11^ on a large scale and the straightforward deprotection/desulfinylation under mild acidic conditions, along with the possibility of recycling the *tert*-butanesulfinamide group.^12^ This has significantly facilitated the use of these imines for obtaining the corresponding enantioenriched primary amines. These amine derivatives have proven valuable in the synthesis of enantioenriched N*-*heterocycles,^13^ natural alkaloids,^14^ and other biologically active compounds.^15^

Normally, these *N*-*tert*-butanesulfinylazadienes react by the N=C double bond, and there is no example where the reactivity takes place by the conjugated C=C bond. Encouraged to explore an alternative reactivity pattern, we envisioned the use of the *N-tert*-butanesulfinylimine group as an electron-withdrawing group in 1-azadienes, allowing the C–C double bond to act as a good dipolarophile in 1,3-dipolar cycloadditions with azomethine ylides (Scheme 1B).

The reaction between (*S*)-*N*-*tert*-butanesulfinyl imine **1a** and glycine α-imino ester derivative **2a** was chosen as the model system. Initially, different silver and copper sources were evaluated as catalysts (Table 1) using Et_3_N as the additive and toluene (0.1 M) as solvent. While copper salts did not provide any reactivity (Table 1, entries 4–5), silver salts were able to promote this reaction, affording the desired cycloadduct **3aa** with high conversions and moderate to high regio- and diastereoselectivities (entries 1–3). Then, different amounts of reagents and concentrations were evaluated (for details, see Supporting Information), allowing us to obtain quantitative conversion and high diastereoselectivity by using Ag_2_CO_3_ as catalyst and 2 equiv of imino ester **2aa** and increasing the concentration to 0.4 M.

**Table 1:**
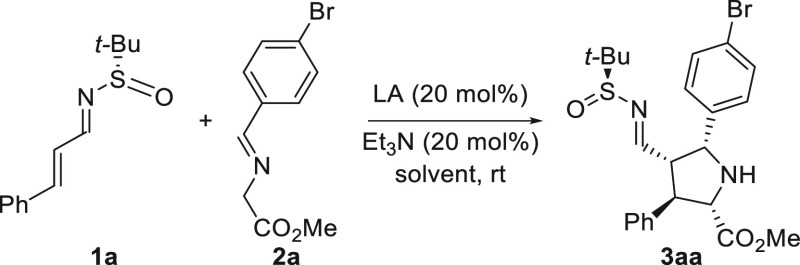
Optimization Reaction Conditions^a^

Entry	LA	Solvent	Conv.^b^ (%)	dr^c^ (%)
1	Ag_2_CO_3_	Toluene	81	92:8
2	AgSbF_6_	Toluene	79	66:34
3	AgOAc	Toluene	79	86:14
4	Cu(OTf)_2_	Toluene	<5	-
5	[(CH_3_CN)_4_Cu]PF_6_	Toluene	<5	-
6^d^	Ag_2_CO_3_	Toluene	>95	92:8
7^d^	Ag_2_CO_3_	THF	>95	88:12
8^d^	Ag_2_CO_3_	CH_3_CN	>95	78:22
9^d^	Ag_2_CO_3_	CH_2_Cl_2_	71	92:8
10^d^	Ag_2_CO_3_	H_2_O	75	59:41
11^d^,^e^	Ag_2_CO_3_	Toluene	>95	92:8
12^d^,^e^,^f^	Ag_2_CO_3_	Toluene	>95	92:8
13^d^,^g^	Ag_2_CO_3_	Toluene	>95	nd

a Reactions were performed with *N*-*tert*-butanesulfinyl imine **1a** (0.1 mmol), α-imino ester **2a** (0.1 mmol), catalyst (20 mol %), and Et_3_N (20 mol %) in toluene (0.1 M) at room temperature for 24 h.

b Conversions were measured by ^1^H NMR of the crude reaction.

c dr was measured by ^1^H NMR of the crude reaction.

d Reaction was performed in toluene (0.4 M) without Et_3_N.

e Ag_2_CO_3_ was reduced to 10 mol %.

f Reaction was performed with (*R*)-*N-tert-*butanesulfinyl imine **1** to generate the enantiomer *ent***-3aa**.

g Reactions were performed with *N*-*p*-tolylsulfinyl imine **1a′** (LA = Lewis acid, nd = not determined).

Further optimization was performed employing a variety of solvents (Table 1, entries 6–10), even though in most cases another regioisomer was observed in low proportion (<15%). THF and acetonitrile lead to quantitative conversions, although diastereomeric ratios decreased in comparison with toluene (entries 7–8 vs 6). On the other hand, dichloromethane and water provided the desired cycloadduct **3aa** in moderate conversion (entries 9–10). Finally, the catalyst loading could be reduced to 10 mol % without compromising conversion, diastereoselectivity, and reaction time (Table 1, entry 11). Using the best conditions, (*R*)-*N*-*tert*-butanesulfinyl imine **1a** was also evaluated affording the enantiomer *ent***-3aa** with the same conversion and diastereoselectivity results (Table 1, entry 12). The importance of the *N*-*tert*-butanesulfinyl imine group was demonstrated when its analogue *N*-*p*-tolylsulfinimine **1a′** was used under the best reaction conditions, giving rise to a complex mixture of products and diastereoisomers (Table 1, entry 13).

Having determined the best reaction conditions, we investigated the scope of the reaction by using a wide variety of imino esters **2** and *N*-*tert*-butanesulfinyl imines **1** (Scheme 2). A selection of aryl-substituted imino ester **2** bearing electron-donating and electron-withdrawing groups were successfully tested, affording adducts **3aa**–**3ag** in moderate to good yields for the isolated major diastereoisomer (30–83%), high regioselectivities, and good to excellent diastereomeric ratios. The reaction could be scaled up to 1 mmol for the synthesis of cycloadduct **3aa** requiring a 36 h reaction time without compromising yield, diastereoselectivity, and reaction time. The thienyl-substituted heteroaromatic imino ester **2h** gave rise to the cycloadduct **3ah** with good regio- and diastereoselectivity and with moderate yield. On the other hand, while the imino ester containing the bulkiest *t*-butyl ester group leads to the corresponding cycloadduct **3ai** in moderate regioselectivity, good yield, and excellent diastereomeric ratio, the imino ester with the benzyl ester group provided the adduct **3aj** in high diastereomeric ratio and regioselectivity. The regioisomer **3ai′** was isolated in moderate yield (20%). The alanine derivative iminoester was also tolerated, affording the cycloadduct **3ak** in moderate yield and diastereoselectivity. Apart from the imino ester, we also evaluated different *N*-*tert*-butanesulfinyl imines. Cinnamaldehyde *N*-*tert*-butanesulfinyl imine derivatives possessing electron-donating and electron-withdrawing groups were well tolerated, leading to the cycloadducts **3ba**–**di** in moderate to good yields, moderate to excellent regioselectivities, and high to excellent diastereomeric ratios. The acrolein *N*-*tert*-butanesulfinyl imine derivative provided the cycloadduct **3ga** in moderate yield with low diastereomeric ratio and could be isolated with an 80:20 ratio of dr. Moreover, the aliphatic (*E*)-crotonaldehyde and (*E*)-2-pentenal *N*-*tert*-butanesulfinyl imine derivatives afforded cycloadducts **3ea**–**fa** in high yields and excellent regioselectivities and diastereomeric ratios. Finally, α,β-unsaturated ketones *N*-*tert*-butanesulfinyl imine derivatives were also allowed to obtain the **3ha**–**ia** cycloaducts in excellent regioselectivities, moderate yields, and high diastereomeric ratios. Isomerization experiments were also carried out to explain some lower diastereomeric ratios. Reaction to synthesize **3ad** was monitored at different times, exhibiting lower diastereoselectivity depending on the reaction time (for details, see Supporting Information). Cycloadducts **3aa** and **3ai′** could be crystallized, and their absolute configurations were elucidated by XRD analysis. Assuming a uniform reaction pathway, the absolute configuration of the other products **3** was assigned by analogy.

**Scheme 2:**
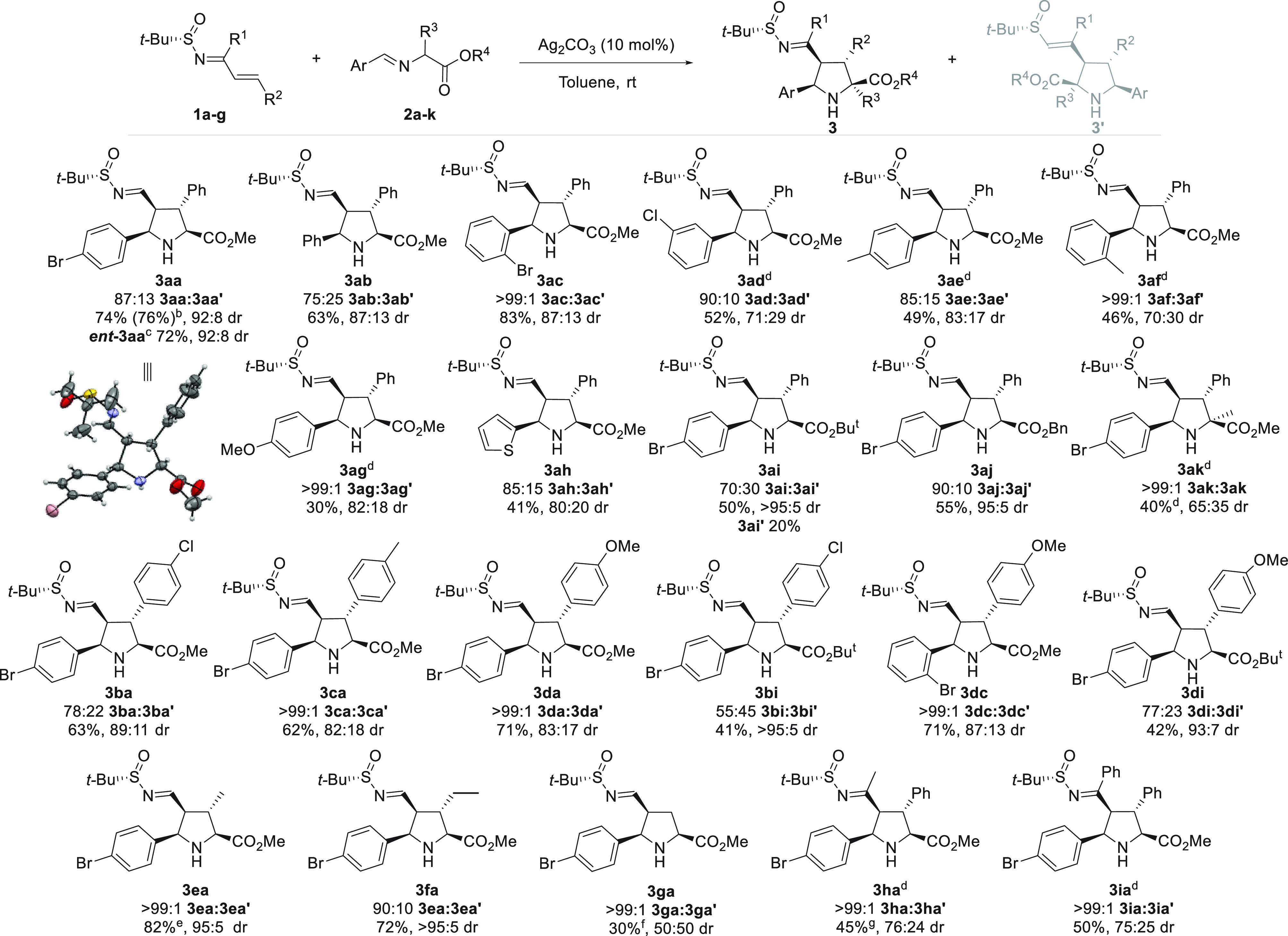
Substrate Scope Reactions were performed with (*S*)-*N*-*tert*-butanesulfinyl imine **1** (0.3 mmol), α-imino ester **2** (0.6 mmol), and Ag_2_CO_3_ (10 mol %) in toluene (0.4 M) at room temperature. Yields (isolated products after flash column chromatography), dr, and regioselectivities determined by ^1^H NMR or LRMS analysis and reaction time are shown in the SI for each product. The second value of dr refers to a mixture of different diastereoisomers. Reaction was performed on a 1 mmol scale. Reaction performed with (*R*)-*N*-*tert*-butanesulfinyl imine **1** to generate the corresponding enantiomer *ent***-3aa**. 5% Et_3_N was employed. Yield refers to a mixture of diastereoisomers (95:5 dr). Yield refers to a mixture of diastereoisomers (80:20 dr). Yield refers to a mixture of diastereoisomers (90:10 dr).

Cycloadducts **3** were easily transformed in appealing derivatives (Scheme 3). For example, *N-*allyl-substituted derivatives **4** were prepared in moderate yield by treatment with an excess (1.5 equiv) of allyl bromide, in the presence of indium metal, in THF at 60 °C for 16 h, avoiding the isomerization of the stereogenic centers. The *N*-*tert*-butanesulfinylimine group of cycloadducts **3aa** and **3dc** was easily reduced with sodium borohydride to afford *N*-*tert*-butanesulfinyl amine derivatives **5** in quantitative yields. Additionally, the removal of the *tert*-butanesulfinyl group was carried out under acidic conditions in Et_2_O, allowing us to produce the cyclization step to afford the bridged 3,6-diazabicyclo[3.2.1]octanes **6** in excellent overall yields.

**Scheme 3:**
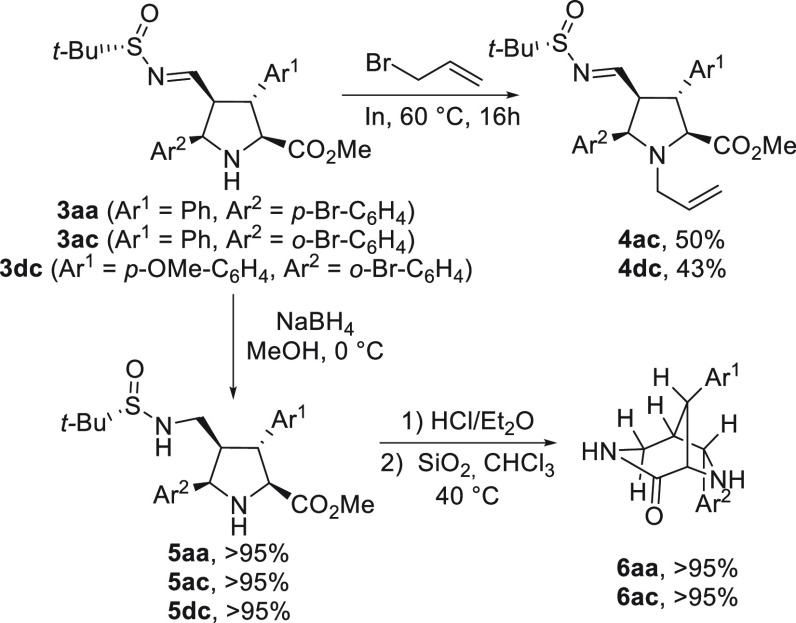
Synthetic Transformations of Adducts **3**

Moreover, to demonstrate the applicability of this new family of densely substituted pyrrolidines, cycloadduct **5aa** was evaluated as an organocatalyst in the asymmetric direct aldol reaction between cyclohexanone **7** and 4-nitrobenzaldehyde **8** (Scheme 4). The aldol adduct **9** was obtained in quantitative conversion and moderate diastereo- and enantioselective ratios (>95% conversion, 78:22% dr, and 68:32 er) in the presence of a 20% catalyst.

**Scheme 4:**
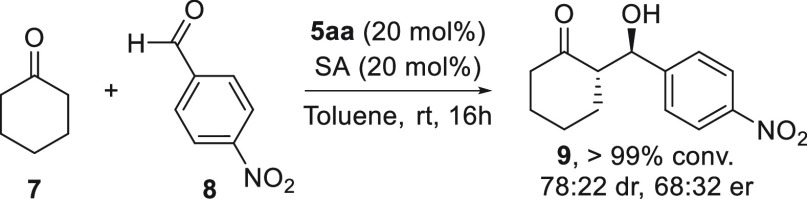
Aldol Reaction Organocatalyzed by **5aa**

Finally, to understand the influence of the *N-tert*-butanesulfinyl group on the diastereoselectivity of this reaction, DFT calculations were performed at the B3LYP level of theory (see Supporting Information for additional details). The computational analysis (DFT, B3LYP level) revealed that a notable interaction can exist between the oxygen atom of the sulfinyl group and the silver atom of the metallodipole (Scheme 5). Apparently the **TS***endo*_**down**_ is more compact, and possessed more energy, than the corresponding **TS***endo*^**up**^. During the optimization of the geometries of the **TS***endo*_**down**_ a very important steric interaction was observed between the *tert-*butyl group and the benzylidene moiety of the **2b-Ag**_**dipole**_, which was negligible in the approach **TS***endo*^**up**^. In fact, this feature distorted the original planarity of the metallodipole to reach the minor diastereomeric structure **3ab′**. In both **TS**s a typical asynchrony was detected, being the incipient carbon–carbon bond, originated by the 1,4-addition, slightly shorter than the second carbon–carbon bond (produced by the Mannich reaction). The difference of 2.8 kcal·mol^–1^ justifies the absolute configuration of the synthesized molecules **3**.

**Scheme 5:**
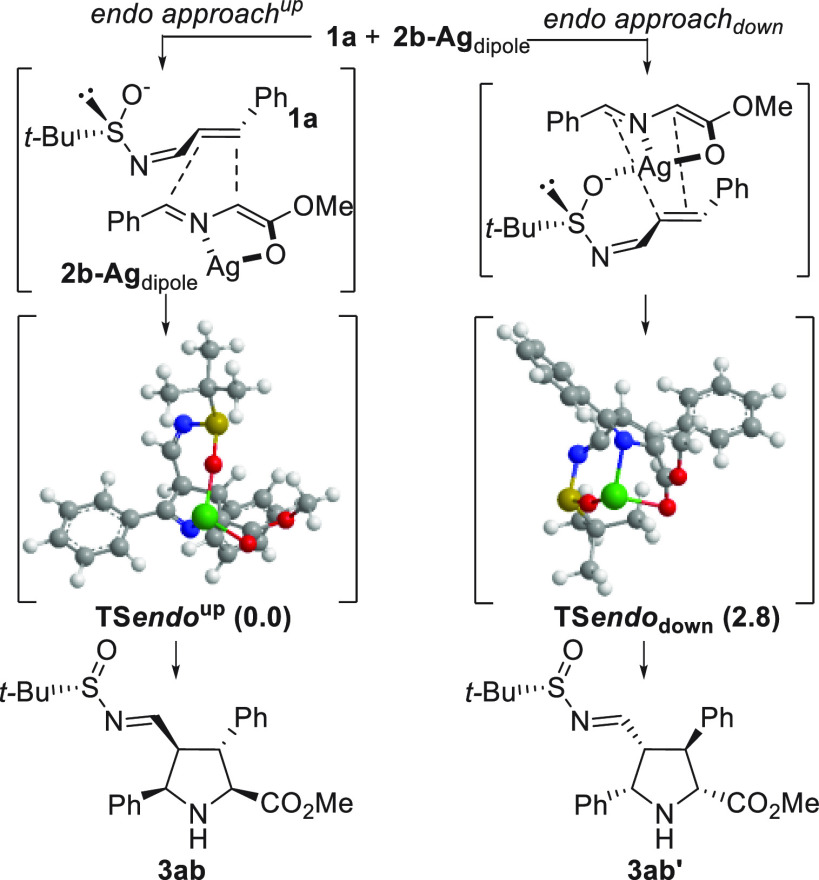
DFT Calculation Analysis of the Two Possible *endo*-Approaches of Alkene **1a** and **2b-Ag**_**dipole**_ The lowest energies (in kcal·mol^–1^) of both **TS**s were optimized at the B3LYP basic level.

In summary, the *N-tert*-butanesulfinylimine group acts as an effective electron-withdrawing group in 1-azadienes, allowing the highly diastereoselective synthesis of a new family of densely substituted pyrrolidines via 1,3-dipolar cycloadditions with azomethine ylides. By using Ag_2_CO_3_ as a catalyst, proline derivatives with up to four stereogenic centers in the pyrrolidine ring have been obtained in moderate to good yields and good to excellent regio- and diastereoselectivities. The (*S*)-configuration of the sulfinyl group is able to induce a (2*S*,3*R*,4*S*,5*R*) absolute configuration in the final pyrrolidines. These derivatives were transformed with high efficiency and selectivity, yielding valuable proline derivatives that can also be employed as organocatalysts. The feasibility of these proline derivatives as organocatalysts has been proved via the asymmetric direct aldol reaction between cyclohexanone and 4-nitrobenzaldehyde, giving rise to the aldol adduct with quantitative conversion and moderate enantioselective and diastereoselective ratios. The interaction between the oxygen atom of the sulfinyl group and the silver atom of the W-shaped metallodipole was studied by computational methods to understand the diastereoselectivity of this reaction.

## Data Availability

The data underlying this study are available in the published article and its Supporting Information.

## Supporting Information Available

Experimental details, characterization data, DFT calculations, and NMR spectra (PDF)
