# Supplementary material for: Stereoselective Synthesis of Densely Substituted Pyrrolidines via a [3 + 2] Cycloaddition Reaction between Chiral N-tert-Butanesulfinylazadienes and Azomethine Ylides
Source: Org Lett. 2023 Oct 4;25(45):8051–6. doi: 10.1021/acs.orglett.3c02572 (PMC10661044; doi:10.1021/acs.orglett.3c02572)
Supplement: Supplementary file 1 — ol3c02572_si_001.pdf [file ol3c02572_si_001.pdf]

Stereoselective Synthesis of Densely Substituted Pyrrolidines via a [3+2] Cycloaddition Reaction between Chiral *N-tert*-Butanesulfinylazadienes and Azomethine Ylides

Ester Blanco-López[a], Francisco Foubelo[a], María de Gracia Retamosa*,[a] and José M. Sansano*,[a]

[a] E. Blanco-López, Dr. M. G. Retamosa, Prof. J. M. Sansano, Prof. F. Foubelo. Departamento de Química Orgánica, Centro de Innovación en Química Avanzada (ORFEO-CINQA) and Institute of Organic Synthesis. Universidad de Alicante. 03080-Alicante, Spain

Email: jmsansano@ua.es

Email: gracia.retamosa@ua.es

TABLE OF CONTENTS

1 General Remarks S2

2 General Procedure for the synthesis of starting materials S3

2.1 General Procedure for the synthesis of α-Imino Esters S3

2.2 General Procedure for the synthesis of *N*-*tert*-butanesulfinyl Imines S3

3 Optimization conditions for the 1,3-Dipolar Reaction S4

3.1 Screening of different metallic salts and bases for the 1,3-Dipolar Reaction.. S4

3.2 Screening of different conditions for the reaction. S5

4 General Procedure for the synthesis of **3**. S5

4.1 Yields and reaction time for the different products. S6

5 Transformation reactions S15

5.1 General procedure for the allylation reaction S15

5.2 General procedure for the reduction of the imine S16

5.3 General procedure for intramolecular cyclization S18

6 Isomerization experiments S19

7 Aldol reaction S20

8 NMR spectra S22

9 X-Ray diffraction structures S56

9.1 X-Ray diffraction of **3aa** (CCDC 2285841) S56

9.2 X-Ray diffraction of **3ai'** (CCDC 2292927) S57

10 Experimental for DFT calculations S58

1 General Remarks

(S_S)-*tert*-Butanesulfinamide and (R_S)-*tert*-Butanesulfinamide was a gift of MEDALCHEMY SL (> 99% ee by chiral HPLC on a Chiracel AS column, 90:10 n-hexane/i-PrOH, 1.2 mL/min, λ=222 nm). Unless otherwise indicated, reagents and substrates were purchased from commercial suppliers. Analytical TLC was performed Schleicher & Schuell F1400/LS 254 silica gel plates, and were visualized under UV light (λ = 254 nm) and with phosphomolybdic acid (PMA). Flash column cromatography was carried out on column silica gel 60 Å (particle size 40-60 μm) using EtOAc and Hexane as eluent. Melting points are uncorrected and were determined with a Reichert Thermovar hot plate apparatus. Optical rotations were measured using a JASCO P-1030 or JASCO DIP-1000 polarimeter with a thermally jacketed 5 cm cell at approximately 23 °C and concentrations (c) are given in g/100 mL. Infrared analyses were performed with a spectrophotometer equipped with an ATR component using a Nicolet 510 P-FT; wavenumbers are given in cm^{-1}. Low-resolution electron impact mass spectra (EI) were obtained at 70 eV using an Agilent 5977B/MSD by injection or DIP; fragment ions in m/z with relative intensities (%) in parentheses. High-resolution mass spectra (HRMS) were carried out in the electron impact mode (EI) at 70 eV, and on an apparatus equipped with a quadrupole time of flight (QTOF) analyzer and the samples were ionized by ESI techniques and introduced through an ultra high pressure liquid chromatography (UPLC) model. ^{1}H NMR spectra were obtained using a Bruker AC-300 or AC-400 and were recorded at 300 or 400 MHz. ^{13}C NMR were recorded at 75 or 101 MHz. $CDCl_3$ was used as solvent and TMS as internal standard (0.00 ppm). Structural assignments were made with additional information from gCOSY, and gHSQC experiments. The data is being reported as: s = singlet, d = doublet, t = triplet, q = quartet, m = multiplet or unresolved, br s = broad signal, coupling constant(s) in Hz, integration. ^{13}C NMR spectra were recorded with ^{1}Hdecoupling at 100 MHz and referenced to $CDCl_3$ at 77.16 ppm. DEPT-135 experiments were performed to assign CH, CH_2 and CH_3. All coupling constants (J) are given in Hertz (Hz) and chemical shifts in ppm. For X-Ray diffraction analyses, Agilent Technologies Super-Nova Atlas Dual Source diffractometer was employed, equipped with two X-ray microphonts, Cu and Mo, CCD area detector and accessory for low temperature measurements.

2 General Procedure for the synthesis of starting materials

2.1 General Procedure for the synthesis of α-Imino Esters

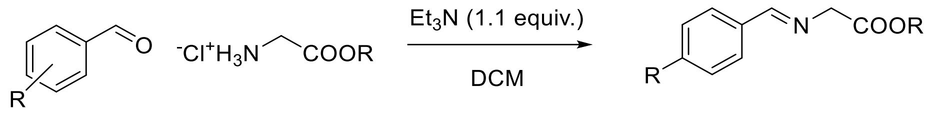

The corresponding amino ester (1.1 mmol) aldehyde (1 mmol) were dissolved in DCM (0.5 M, 2 mL) and Et_3N (1.1 mmol) was added. The mixture was stirred for 16 h at room temperature (25 °C). The mixture was quenched by NaCl (saturated aq.), extracted with DCM (3 × 10 mL), and dried with $MgSO_4$. The crude residue was obtained after evaporation (15 Torr) of the solvent and was used without purification. [1,2]. Spectroscopical data are according to the literature [3]

2.2 General Procedure for the synthesis of *N-tert*-butanesulfinyl Imines

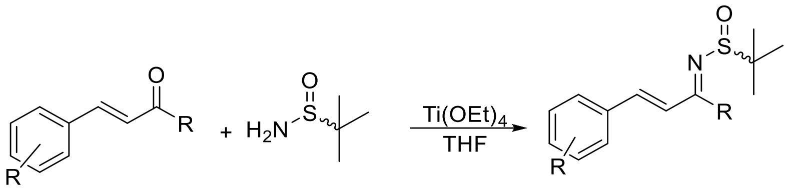

The corresponding *tert*-butanesulfinamide (0.605 g, 5 mmol) and the carbonyl compound (5.5 mmol) were dissolved in dry THF (0.25 M, 20 mL) under argon at 23 °C. Titanium tetraethoxide (2.2281 g, 2.095 mL, 10 mmol) was slowly added to the solution. The reaction mixture was stirred for 12 h at room temperature for aldehydes and at 66 °C for 5 h for ketones. The resulting mixture was hydrolyzed with brine (30 mL), extracted with ethyl acetate (3 × 15 mL), dried with anhydrous $MgSO_4$, and the solvent evaporated (15 Torr). The residue was purified by column chromatography (silica gel, hexane/ethyl acetate) to yield pure compounds **1a-g**. [4,5]. Spectroscopical data are according to the cited literature [6,7.]

[1] Caleffi, G.S.; Larrañaga, O.; Ferrándiz-Saperas, M.; Costa, P.R.R.; Nájera, C.; de Cózar, A.; Cossío, F.P. and Sansano, J.M. Switching diastereoselectivity in catalytic enantioselective (3+ 2) cycloadditions of azomethine ylides promoted by metal salts and privileged segphos-derived ligands. *J. Org. Chem.* **2019,** *84*, 10593–10605.

[2] García-Mingüens, E.; Ferrándiz-Saperas, M.; Retamosa, M. de Gracia; Nájera, C.; Yus, M. and Sansano J.M. Enantioselective 1,3-Dipolar Cycloaddition Using (Z)-α-Amidonitroalkenes as a Key Step to the Access to Chiral cis-3,4-Diaminopyrrolidines. *Molecules* **2022**, *27*, 4579.

[3] López-Pérez, A.; Adrio, J. and Carretero, J. C. Bis-Sulfonyl Ethylene as Masked Acetylene Equivalent in Catalytic Asymmetric [3 + 2] Cycloaddition of Azomethine Ylides. *J. Am. Chem. Soc*. **2008,** *130*, 10084–10085.

[4] a) Liu, G.; Cogan, D. A. and Ellman, J. A. Catalytic Asymmetric Synthesis of tert-Butanesulfinamide. Application to the Asymmetric Synthesis of Amines. *J. Am.Chem.Soc*. **1997**, *119*, 9913–9914 b) Liu, G.; Cogan, D.A.; Owens, T.D.; Tang, T.P. and Ellman, J.A. Synthesis of Enantiomerically Pure N-tert-Butanesulfinyl Imines (tert-Butanesulfinimines) by the Direct Condensation of tert-Butanesulfinamide with Aldehydes and Ketones *J. Org.Chem.***1999**, *64*, 1278–128.

[5] García-Muñoz, M.J.; Zacconi, F.; Foubelo, F.; and Yus, M. Indium-Promoted Diastereo- and Regioselective Propargylation of Chiral Sulfinylimines. *Eur. J. Org. Chem.,* **2013**, 1287-1295.

[6] Groch, J. R.; Lauta, N.R. and Njardarson, J.T. One Step Selective Counterion Dependent Formation of Conjugated Chiral N-Sulfinylimines from Aldehydes. *Organic Letters* **2023**, *25* (2), 395-399.

[7] Zhang, H.; Li, Y.; Xu, F.; Ren, X.F.; Wang, L.; Zhou, P. and Sun, Z. 2490 Stereoselective Synthesis of γ, δ-Unsaturated β-Amino Sulfones from Ellman's N-tert-Butylsulfinyl Ketimines and Methyl Phenyl Sulfone. *Synlett* **2012**, *23*, 2485–2490.

3 Optimization conditions for the 1,3-Dipolar Reaction

3.1 Screening of different metallic salts and bases for the 1,3-Dipolar Reaction.

Different Silver and Copper salts were evaluated in order to increase conversion and dr for obtaining *endo* diastereomer.

Table S1. Screening of different metallic salts for the 1,3-Dipolar Reaction.

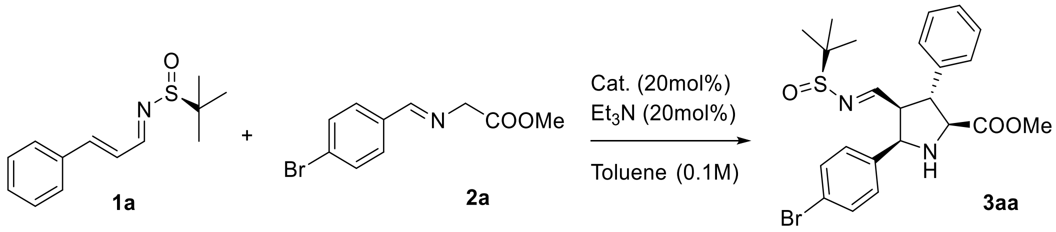

Entry	Catalyst	Conversion[b]	dr[c]
1	Ag_2CO_3	81	92:8[d]
**2**	$AgSbF_6$	79	66:34[d]
**3**	AgOTf	nd	62:38[d]
**4**	CH_3COOAg	79	86:14[d]
**5**	AgF	>95	67:33[d]
**6**	$(CF_3SO_3)_2Cu$ 98%	<5	-
**7**	$[(CH_3CN)_4Cu]PF_6$	<5	-
**8**	$(CF_3SO_3Cu)_2 \cdot C_6H_5CH_3$ (2:1 complex)	<5	-

[a] Reactions were perform using α-Imino Ester **2a** (0.1 mmol, 25.6 mg), *N*-*tert*-butanesulfinyl Imine **1a** (0.1 mmol, 23.5 mg), in presence of the corresponding catalyst (20 mmol%) and Et_3N (20 mmol%, 2.5µL) in toluene (0.1 M, 1 mL) at rt for 24 h. [b] Conversions were measured by ^1H NMR of crude reaction mixtures. [c] was measured by ^1H NMR of crude reaction mixtures. [d] Diastomer mixture.

3.2 Screening of different conditions for the reaction.

Table S2. Screening of different conditions for the reaction. Changes in solvent, temperature, molarity and imino ester equivalents.

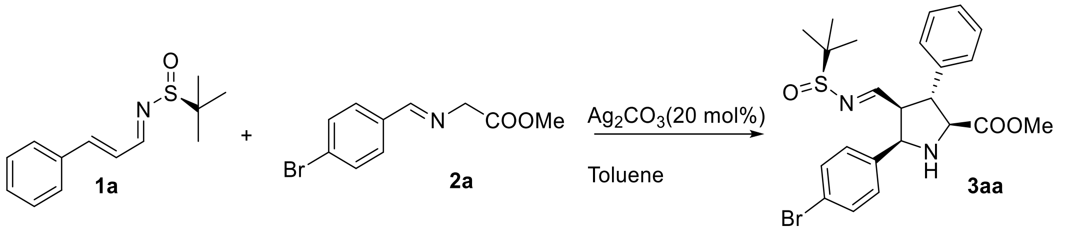

Entry	Solvent	Imino ester equiv.	Molarity	Conversion[b]	dr [c]
**1**	Toluene	1	0.2	43	92:8 [d]
**2**[e]	Toluene	1	0.2	80	81:19 [d]
**3**	Toluene	2	0.2	68	92:8 [d]
**4**	Toluene	1	0.4	80	92:8 [d]
**5**	Toluene	2	0.4	>95	92:8 [d]
**6**	THF	2	0.4	>95	88:12 [d]
**7**	CH_3CN	2	0.4	>95	78:22 [d]
**8**	CH_2Cl_2	2	0.4	71	92:8 [d]
**9**	H_2O	2	0.4	75	59:41 [d]
**10**[f]	Toluene	2	0.4	>95	92:8 [d]
**11**[f,g]	Toluene	2	0.4	>95	92:8 [d]

[a] Reactions were perform using α-Imino Ester **2a** (0.1 mmol/0.2 mmol, 25.6/51.2 mg), *N*-*tert*-butanesulfinyl Imine **1a** (0.1 mmol, 23.5 mg), in presence of Silver Carbonate (20 mol%, 5.51 mg) in toluene at rt for 24 h. [b] Conversions were measured by ^{1}H NMR of crude reaction mixtures. [c] was measured by ^{1}H NMR of crude reaction mixtures. [d] Diastomer mixture. [e] Entry 2 was performed at 40ºC in a sand bath. [f] mol% of the catalyst Ag_2CO_3 was reduced to the 10 mol%. [g] The reaction was performed using α-Imino Ester **2a** (2 mmol, 512 mg), *N*-*tert*-butanesulfinyl Imine **1a** (1 mmol, 235 mg), in presence of Silver Carbonate (20 mol%, 27.5 mg) in toluene at rt for 36 h.

4 General Procedure for the synthesis of 3.

A mixture of the corresponding *N*-*tert*-butanesulfinyl imine **1a-g** (0.3 mmol), *α*-imino ester **2a-k** (0.6 mmol) and Silver Carbonate (10 mol %, 8.3 mg) in toluene (0.4 M, 0.75 mL) was stirred in a round bottom vial at room temperature and monitorized by TLC. In some cases, were necessary the use of Et_3N (5 mol %, 2µL). Then the resulting mixture was evaporated and the residue was purified by column chromatography (silica gel, hexane/EtOAc).

4.1 Yields and reaction time for the different products.

Table S3. Summary of reaction times, base conditions, regio and diastereomeric ratios and yields.

SCOPE	3	Time of reaction	Et_3N	3:3’	dr	Yield 3
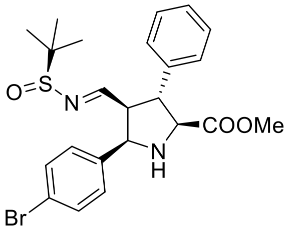	**3aa**	24 h<br><br>36 h for 1 mmol scale	No	**87**:13	**92**:8	108.8 mg<br>74% by column chromatography<br>60% by Et_2O recrystallization<br><br>**1 mmol scale**:<br>373.5 mg<br>76% by column chromatography
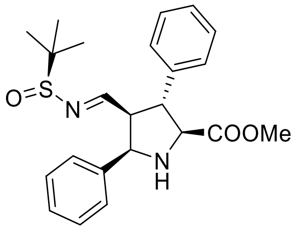	**3ab**	48 h	No	**75**:25	**87**:13	77.8 mg<br>63%
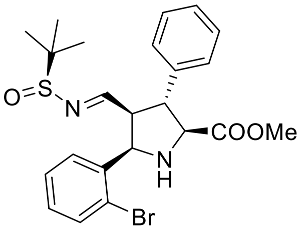	**3ac**	24 h	No	>**99**:1	**87**:13	122.2 mg<br>83%
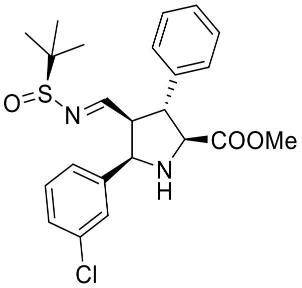	**3ad**	48 h	5%	**90:**10	**71**:29	69.6 mg<br>52%
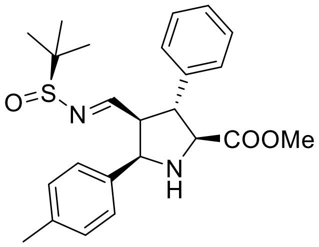	**3ae**	72 h	5%	**85**:15	**83**:17	60.4 mg<br>49%
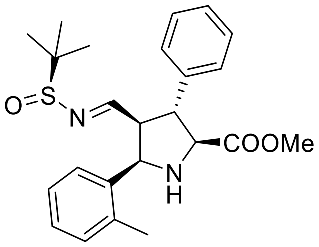	**3af**	72 h	5%	>**99**:1	**70**:30	Conversion:<br>88%<br>Yield:<br>56.7 mg<br>46%

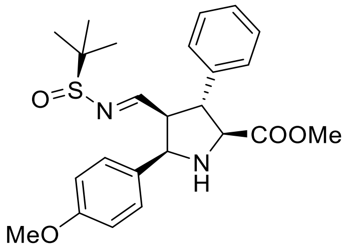	**3ag**	72 h	5%	>**99**:1	**82**:18	Conversion: 51%<br>Yield:<br>39.8 mg<br>30%
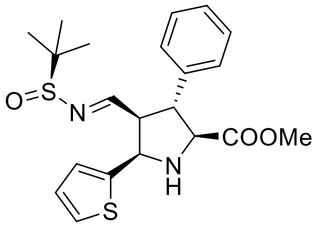	**3ah**	24 h	No	**85**:15	**80**:20	51.4 mg<br>41%
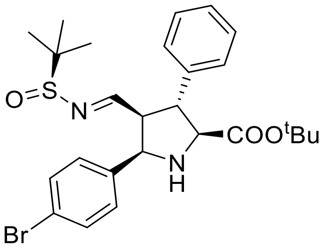	**3ai**	48 h	No	**70**:30	>**95**:5	79.8 mg, 50%<br>(31.9 mg, 20%<br>for **3ai'**)
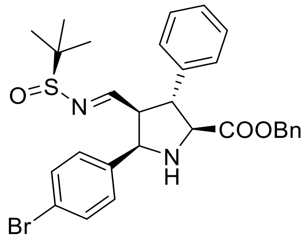	**3aj**	24h	No	**90**:10	**95**:5	93.4 mg<br>55%
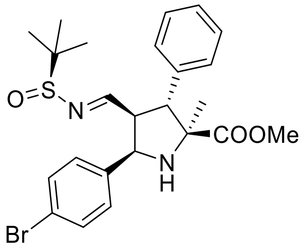	**3ak**	72 h	5%	>**99**:1	**65**:35	Conversion: 75%<br>Yield: 60.4 mg,<br>40 %<br>dr after<br>purification<br>80:20
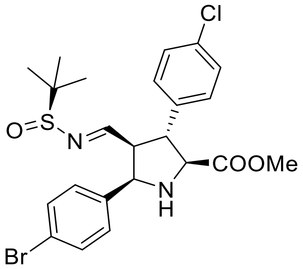	**3ba**	24 h	No	**78**:22	**89**:11	99.0 mg<br>63%
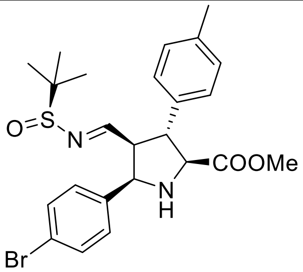	**3ca**	24 h	No	>**99**:1	**82**:12	93.7 mg<br>62%

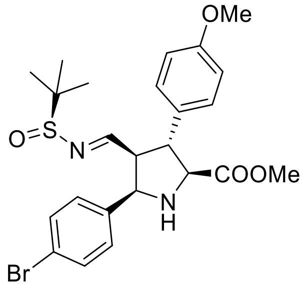	**3da**	72 h	No	>**99**:1	**83**:17	110.7 mg<br>71%
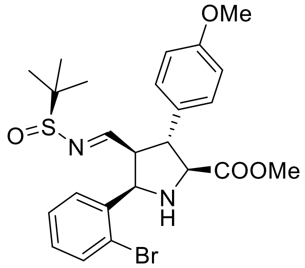	**3dc**	24 h	No	>**99**:1	**87**:13	109.2 mg<br>71%
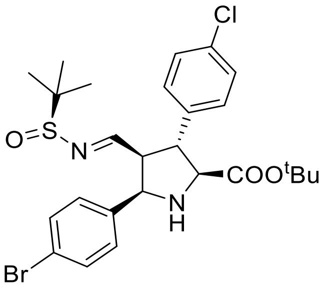	**3bi**	48 h	No	**55**:45	>**95**:5	69.6 mg<br>41%
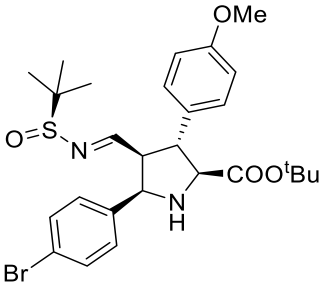	**3di**	24 h	No	**77**:23	**93**:7	70.6 mg<br>42%
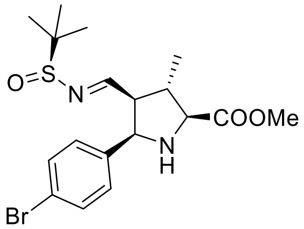	**3ea**	24 h	No	>**99**:1	**95**:5	105.6 mg<br>82%<br>dr after purification<br>95:5
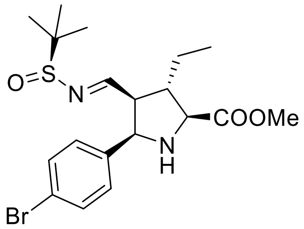	**3fa**	24 h	No	**90**:10	>**95**:5	95.7 mg<br>72%
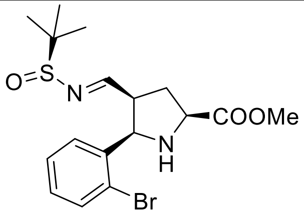	**3ga**	24 h	No	>**99**:1	**50**:50	37.3 mg<br>30%<br>dr after purification<br>80:20

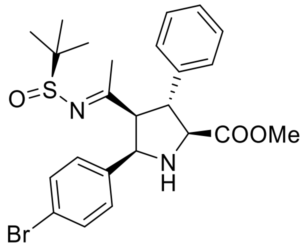	**3ha**	72 h	5 %	>**99**:1	**76**:24	Conversion: 84% Yield: 68.0 mg 45 % dr after purification 90:10
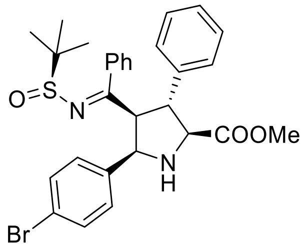	**3ia**	72 h	5%	>**99**:1	**75**:25	85 mg 50%

3aa. Methyl (2*S*,3*R*,4*S*,5*R*)-5-(4-bromophenyl)-4-((*E*)-(((*S*)-*tert*-butylsulfinyl)imino)methyl)-3-phenylpyrrolidine-2-carboxylate.

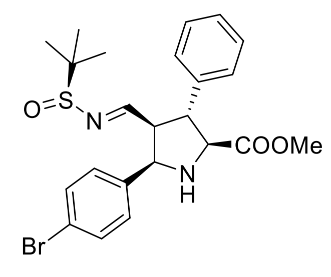

White solid; **Mp**: 75-76 °C; ***R*F** 0.55 (1:1 AcOEt/Hex); **[α]$^{20}_{D}$** +79.9 (c 0.63, CH_2Cl_2); **IR** (neat) ν: 2958, 1727, 1616, 1484, 1392, 1068, 1006, 817, 755 cm^{-1} ; **^{1}H NMR** (400 MHz, $CDCl_3$) δ 7.49 – 7.45 (m, 3H, Ar*H* and N=C*H*), 7.34 – 7.23 (m, 7H, Ar*H*), 4.90 (d, *J* = 7.6 Hz, 1H, NHC*H*Ar), 4.13 (d, *J* = 8.6 Hz, 1H, NHC*H*CO), 3.82 – 3.73 (m, 2H, NCHC*H*C*H*), 3.71 (s, 3H, OC*H*3), 1.60 (s, 1H, N*H*) 0.87 (s, 9H, 3xCH_3); **^{13}C NMR** (101 MHz, $CDCl_3$) δ 173.6 (C, *C*=O), 168.1 (CH, N=CH), 139.4(C, Ar*C*), 139.2(C, Ar*C*), 132.4(CH, Ar*C*), 129.3(CH, Ar*C*), 129.3(CH, Ar*C*), 128.4(CH, Ar*C*), 127.8(CH, Ar*C*), 122.2(*C*Br), 67.9(CH, NH*C*HCO), 64.6(CH, Ar*C*HNH), 58.0(CH, *C*HPh), 56.9(C, OS*C*(CH_3)3), 52.7(CH, O*C*H3), 51.6(CH, *C*HPh), 22.4(CH_3, OSC(*C*H3)3); **MS** (EI) *m/z*: 384 (M^+-$C_4H_{10}OS$, 3%), 327 (69), 325 (73), 257 (97), 255 (100), 227 (26), 225 (26); **HRMS** (ESI-TOF): m/z calcd $C_{19}H_{19}BrN_2O_3S$ [M^+ -C_4H_8] 434.03, found 436.0259.

***ent*-3aa'.** Methyl (2*S*,3*R*,4*S*,5*R*)-5-(4-bromophenyl)-4-((*E*)-(((*R*)-*tert*-butylsulfinyl)imino)methyl)-3-phenylpyrrolidine-2-carboxylate.

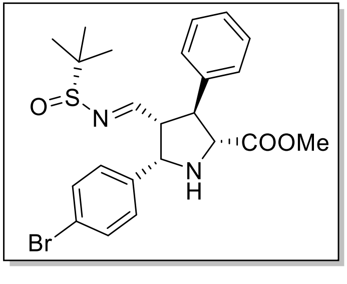

White solid; **Mp**: 75-76 °C; ***R*F** 0.55 (1:1 AcOEt/Hex); **[α]$^{20}_{D}$** **+79.9** (0.60, CH_2Cl_2); **IR** (neat) ν: 2958, 1727, 1616, 1484, 1392, 1068, 1006, 817, 755 cm^{-1}; **^{1}H NMR** (300 MHz, $CDCl_3$) δ [7.49 – 7.44 (m, 3H), 7.34 – 7.23 (m, 7H) Ar*H* and N=C*H*], 4.90 (d, *J* = 7.5 Hz, 1H, , NHC*H*Ar), 4.13 (d, *J* = 8.7 Hz, 1H, NHC*H*CO), 3.84 – 3.73 (m, 2H, NCHC*H*C*H*), 3.70 (s, 3H, OC*H*3), 0.86 (s, 9H, 3xCH_3); **^{13}C NMR** (101 MHz, $CDCl_3$) δ 173.6 (C, *C*=O), 168.1 (CH, N=*C*H), 139.4(C, Ar*C*), 139.2(C, Ar*C*), 132.4(CH, Ar*C*), 129.3(CH, Ar*C*), 129.3(CH, Ar*C*), 128.4(CH, Ar*C*), 127.8(CH, Ar*C*), 122.2(*C*Br), 67.9(CH, NH*C*HCO), 64.6(CH, Ar*C*HNH), 58.0(CH, *C*HPh), 56.9(C, OS*C*(CH_3)3), 52.7(CH, O*C*H3), 51.6(CH, *C*HPh), 22.4(CH_3, OSC(*C*H3)3). **MS** (EI) *m/z*: 384 (M^+-$C_4H_{10}OS$, 3%), 327 (69), 325 (73), 257 (97), 255 (100), 227 (26), 225 (26); **HRMS** (ESI-TOF): m/z calcd $C_{19}H_{19}BrN_2O_3S$ [M^+ -C_4H_8] 434.03, found 436.0259.

3ab. Methyl (2*S*,3*R*,4*S*,5R)-4-((*E*)-(((*S*)-*tert*-butylsulfinyl)imino)methyl)-3,5-diphenylpyrrolidine-2-carboxylate

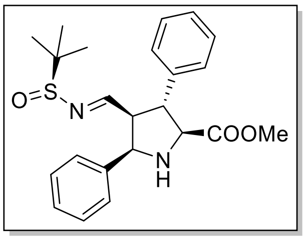

Yellow viscous solid; ***R*F** 0.50 (1:1 AcOEt/Hex); **[α]$^{20}_{D}$** +102.2 (c 0.77, CH_2Cl_2); **IR** (neat) ν: 2954, 1739, 1616, 1492, 1365, 1079, 1022, 856, 755 cm^{-1}; **^{1}H NMR** (300 MHz, $CDCl_3$) δ 7.47 – 7.41 (m, 3H), 7.37 (ddd, *J* = 7.5, 6.6, 1.3 Hz, 3H), 7.33 (d, *J* = 1.2 Hz, 1H), 7.32 – 7.29 (m, 3H), 7.28 – 7.26 (m, 1H), 4.97 (d, *J* = 7.7 Hz, 1H), 4.16 (d, *J* = 8.7 Hz, 1H), 3.86 – 3.77 (m, 2H), 3.73 (s, 3H), 0.88 (s, 9H); **^{13}C NMR** (101 MHz, $CDCl_3$) δ 173.7, 168.6, 139.8, 139.7, 129.3, 129.2,128.4, 128.3, 127.7, 127.50, 68.0, 65.4, 58.4, 56.9, 52.7, 51.9, 22.4; **MS** (EI) *m/z*: 248 (M$^+$- $C_6H_{12}O_3S$, 22%), 247 (97), 230 (8), 177(100), 169 (6); **HRMS** (ESI-TOF): m/z Calcd for $C_{17}H_{15}N_2$ [M+ -$C_6H_{13}O_3S$] 247.1242, found 247.1235.

3ac. Methyl (2*S*,3*R*,4*S*,5*R*)-5-(2-bromophenyl)-4-((*E*)-(((*S*)-*tert*-butylsulfinyl)imino)methyl)-3-phenylpyrrolidine-2-carboxylate

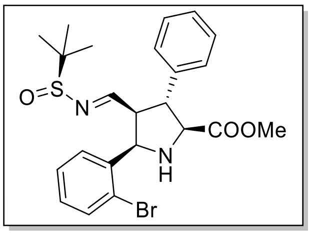

White solid; **Mp**: 40-41°C; ***R*F** 0.65 (1:1 AcOEt/Hex); **[α]$^{20}_{D}$** +117.4 (c 1.62, CH_2Cl_2); **IR** (neat) ν: 2954, 1735, 1616, 1446, 1365, 1079, 1018, 829, 752, 698 cm^{-1}; **^{1}H NMR** (300 MHz, $CDCl_3$) δ 7.84 (dd, *J* = 7.8, 1.7 Hz, 1H), 7.53 (dd, *J* = 7.9, 1.3 Hz, 1H), 7.42 (d, *J* = 5.8 Hz, 1H), 7.36 (d, *J* = 1.3 Hz, 1H), 7.32 (d, *J* = 4.4 Hz, 4H), 7.26 (s, 1H), 7.16 (dd, *J* = 7.7, 1.7 Hz, 1H), 5.25 (d, *J* = 7.6 Hz, 1H), 4.11 (d, *J* = 8.6 Hz, 1H), 3.94 – 3.88 (m, 2H), 3.72 (s, 3H), 0.92 (s, 9H); **^{13}C NMR** (101 MHz, $CDCl_3$) δ 173.0, 167.4, 139.7, 138.4, 133.0, 129.5, 129.1, 128.9, 128.2, 127.9, 127.4, 123.1, 67.3, 63.5, 56.5, 55.4, 52.3, 50.4, 22.2; **MS** (EI) *m/z*: 327 (M$^+$-$C_6H_{11}O_3S$, 84%), 325 (85), 257 (98), 255 (100), 226 (17), 224 (17); **HRMS** (ESI-TOF): m/z Calcd for $C_{17}H_{14}BrN_2$ [M+ -$C_6H_{12}O_3S$] 325.0329, found 325.034.

3ad. Methyl (2*S*,3*R*,4*S*,5*R*)-4-((*E*)-(((*S*)-*tert*-butylsulfinyl)imino)methyl)-5-(3-chlorophenyl)-3-phenylpyrrolidine-2-carboxylate.

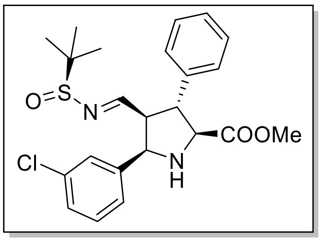

Yellow vicous solid; ***R*F** 0.57 (1:1 AcOEt/Hex); **[α]$^{20}_{D}$** +69.4 (c 0.50, CH_2Cl_2); **IR** (neat) ν: 3328, 3031, 1739, 1616, 1365, 1207, 910, 790 cm^{-1}; **^{1}H NMR** (400 MHz, $CDCl_3$) δ 7.50 – 7.48 (m, 1H), 7.44 (d, *J* = 6.1 Hz, 1H), 7.33 – 7.26 (m, 6H), 7.25 – 7.21 (m, 2H), 4.91 (d, *J* = 8.3 Hz, 1H), 4.13 (d, *J* = 9.0 Hz, 1H), 3.86 – 3.80 (m, 1H), 3.76 – 3.72 (m, 1H), 3.72 (s, 3H), 0.88 (s, 9H); **^{13}C NMR** (101 MHz, $CDCl_3$) δ 173.5, 168.0 , 142.2, 139.5, 135.3, 130.4, 129.2, 128.5, 128.3, 127.8, 127.7, 125.7, 67.8, 64.6, 58.0, 56.9, 52.7, 51.5, 22.4; **MS** (EI) *m/z*: 341 (M$^+$-C_4H_9OS, 2%), 282(20), 281(100), 211(62), 151 (50); **HRMS** (ESI-TOF): m/z Calcd for $C_{17}H_{14}ClN_2$ [M+ -$C_6H_{13}O_3S$] 281.0846, found 281.0841.

3ae. Methyl (2S,3R,4S,5R)-4-((*E*)-(((*S*)-*tert*-butylsulfinyl)imino)methyl)-3-phenyl-5-(p-tolyl)pyrrolidine-2-carboxylate

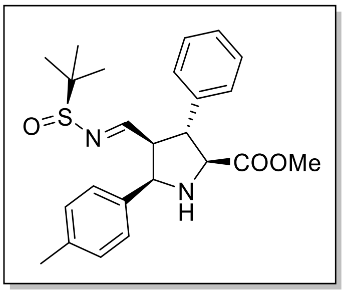

White solid; **Mp**: 92-93°C; ***R*F** 0.38 (1:1 AcOEt/Hex); **[α]$^{20}_{D}$** +116.4 (c 0.56, CH_2Cl_2); **IR** (neat) ν: 2954, 1731, 1619, 1446, 1268, 1214, 1025, 755 cm^{-1}; **^{1}H NMR** (300 MHz, $CDCl_3$) δ 7.45 (d, *J* = 6.1 Hz, 1H), 7.34 – 7.26 (m, 6H), 7.25 – 7.22 (m, 1H), 7.18 – 7.12 (m, 2H), 4.90 (d, *J* = 7.8 Hz, 1H), 4.11 (d, *J* = 8.8 Hz, 1H), 3.83 – 3.71 (m, 2H), 3.70 (s, 3H), 2.31 (s, 3H), 0.86 (s, 9H); **^{13}C NMR** (101 MHz, $CDCl_3$) δ 173.4 , 168.4 , 139.4, 137.6, 136.4, 129.6, 128.8, 128.0, 127.4, 127.0, 67.8, 65.0, 58.1, 56.5, 52.4, 51.7, 22.1, 21.2; **MS** (EI) *m/z*: 262 (M$^+$-$C_6H_{11}O_3S$, 16%), 261 (70), 191 (100), 160 (30); **HRMS** (ESI-TOF): m/z Calcd for $C_{20}H_{21}N_2O_2$ [M+ -C_4H_9OS] 321.1603, found 321.1594.

3af. Methyl (2S,3R,4S,5R)-4-((*E*)-(((*S*)-*tert*-butylsulfinyl)imino)methyl)-3-phenyl-5-(o-tolyl)pyrrolidine-2-carboxylate

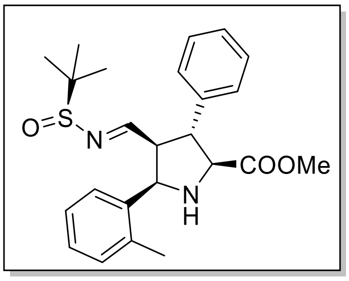

Incolour viscous solid; ***R***$_{\mathbf{F}}$ 0.60 (1:1 AcOEt/Hex); **[α]$^{20}_{D}$** +140.1 (c 0.50, CH_2Cl_2); **IR** (neat) ν: 2954, 1739, 1616, 1454, 1365, 1133, 1079, 1025, 914, 863 cm^{-1}. **^{1}H NMR** (300 MHz, $CDCl_3$) δ 7.77 – 7.72 (m, 1H), 7.39 (d, *J* = 6.6 Hz, 1H), 7.35 – 7.28 (m, 5H), 7.27 – 7.23 (m, 1H), 7.20 (td, *J* = 7.3, 1.5 Hz, 1H), 7.16 – 7.12 (m, 1H), 5.11 (d, *J* = 8.2 Hz, 1H), 4.14 (d, *J* = 9.1 Hz, 1H), 3.86–3.75 (m, 2H), 3.74 (s, 3H, s, 3H), 2.32 (s, 3H), 1.63 (s, 1H), 0.92 (s, 9H); **^{13}C NMR** (101 MHz, $CDCl_3$) δ 173.4, 168.4, 139.9, 137.4, 135.1, 129.3, 128.4, 128.1, 127.7, 127.0, 67.8, 61.5, 56.9, 56.8, 52.6, 51.9, 22.5, 20.1; **MS** (EI) *m/z*: 261 (M^+-$C_6H_{12}O_3S$, 66%), 253 (11), 191 (100), 160 (32); **HRMS** (ESI-TOF): m/z Calcd for $C_{20}H_{20}N_2O_2$ [M+ -$C_4H_{10}OS$] 320.1525, found 320.1525.

3ag. Methyl (2*S*,3*R*,4*S*,5*R*)-4-((*E*)-(((*S*)-*tert*-butylsulfinyl)imino)methyl)-5-(4-methoxyphenyl)-3-phenylpyrrolidine-2-carboxylate

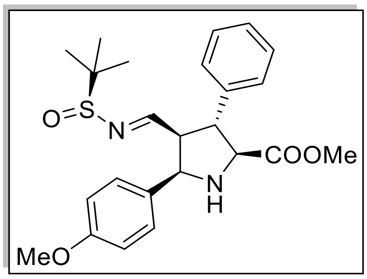

Yellow viscous oil; ***R*$_{\mathbf{F}}$** 0.50 (1:1 AcOEt/Hex); **[α]$^{20}_{D}$** +59.3 (c 0.50, CH_2Cl_2); **IR** (neat) ν: 2954, 1612, 1450, 1365, 825, 759 cm^{-1}; **^{1}H NMR** (400 MHz, $CDCl_3$) δ 7.41 (d, *J* = 6.3 Hz, 1H), 7.28 – 7.25 (m, 2H), 7.24 – 7.15 (m, 5H), 6.81 (d, *J* = 8.7 Hz, 2H), 4.82 (d, *J* = 8.0 Hz, 1H), 4.03 (d, *J* = 9.0 Hz, 1H), 3.75 – 3.72 (m, 1H), 3.71 (s, 3H), 3.66 – 3.59 (m, 4H), 0.80 (s, 9H); **^{13}C NMR** (101 MHz, $CDCl_3$) δ 173.8, 168.7, 159.6, 139.7, 131.9, 129.2, 128.6, 128.4, 127.7, 114.6, 68.1, 64.9, 58.5, 56.8, 55.6, 52.6, 52.0, 22.4; **MS** (EI) *m/z*: 385 (M^+- C_4H_9 68%), 277 (15), 207 (78), 175 (31), 147(100); **HRMS** (ESI-TOF): m/z Calcd for $C_{18}H_{17}N_2O$ [M+ -$C_6H_{13}O_3S$] 277.1341, found 277.1339.

3ah. Methyl (2*S*,3*R*,4*S*,5*R*)-4-((*E*)-(((*S)*-tert-butylsulfinyl)imino)methyl)-3-phenyl-5-(thiophen-2-yl)pyrrolidine-2-carboxylate

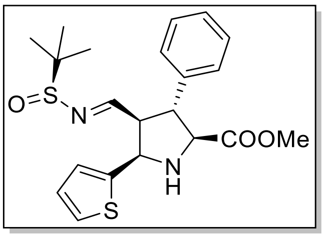

Yellow solid; **Mp**: 72-74°C; ***R*$_{\mathbf{F}}$** 0.65 (1:1 AcOEt/Hex); **[α]$^{20}_{D}$** +19.8 (c 1.42, CH_2Cl_2); **IR** (neat) ν: 3451, 2626, 1731, 1623, 1295, 1110, 956, 914 cm^{-1}; **^{1}H NMR** (300 MHz, $CDCl_3$) δ δ 7.58 (d, *J* = 6.3 Hz, 1H), 7.34 – 7.27 (m, 4H), 7.26 – 7.22 (m, 2H), 7.08 – 7.04 (m, 1H), 6.98 (dd, *J* = 5.1, 3.5 Hz, 1H), 5.16 (d, *J* = 7.8 Hz, 1H), 4.10 (d, *J* = 9.1 Hz, 1H), 3.89 – 3.72 (m, 2H), 3.70 (s, 3H), 0.86 (s, 9H); **^{13}C NMR** (101 MHz, $CDCl_3$) δ 173.0, 167.4, 144.0, 138.6, 128.7, 128.0, 127.4, 127.3, 125.2, 125.1, 67.3, 60.7, 57.8, 56.5, 52.3, 51.2, 21.9 ; **MS** (EI) *m/z*: 310 (M^+- $C_4H_{12}OS$ 1%), 254 (9), 253 (54), 183 (100); **HRMS** (ESI-TOF): m/z Calcd for $C_{17}H_{14}N_2O_2S$ [M+ -$C_4H_{12}OS$] 310.0776 found 310.0777.

3ai. *Tert*-butyl (2*S*,3*R*,4*S*,5*R*)-5-(4-bromophenyl)-4-((*E*)-(((*S*)-tert-butylsulfinyl)imino)methyl)-3-phenylpyrrolidine-2-carboxylate

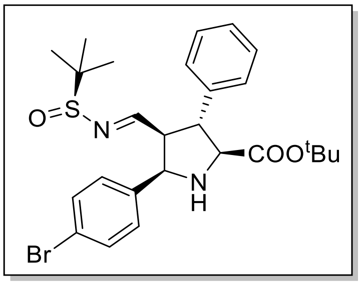

Pale yellow solid; **Mp**: 41-42 °C; ***R*$_{\mathbf{F}}$** 0.63 (1:1 AcOEt/Hex); **[α]$^{20}_{D}$** +71.2 (c 0.64, CH_2Cl_2); **IR** (neat) ν: 2969, 1724, 1619, 1481, 1365, 1076, 1006, 833, 744 cm^{-1}; **^{1}H NMR** (300 MHz, $CDCl_3$) δ 7.47 (d, *J* = 8.5 Hz, 2H), 7.42 (d, *J* = 6.5 Hz, 1H), 7.36 – 7.21 (m, 7H), 4.88 (d, *J* = 8.2 Hz, 1H), 3.99 (d, *J* = 9.4 Hz, 1H), 3.76 (m, 1H), 3.62 (t, *J* = 9.5 Hz, 1H), 1.35 (s, 9H), 0.83 (s, 9H); **^{13}C NMR** (101 MHz, $CDCl_3$) δ 172.4, 168.1, 139.5, 139.3, 132.3, 129.3, 129.0, 128.5, 127.7, 122.2, 82.2, 68.8, 64.7, 58.3, 56.8, 52.3, 28.3, 22.3; **MS** (EI) *m/z*: 475 (M^+-C_4H_9, 8%), 421 (12), 419 (12), 327 (96), 325 (100); **HRMS** (ESI-TOF): m/z calcd for $C_{22}H_{24}N_2O_3S$ [M^+ -C_4H_9] 396.1508, found 396.1501.

3ai'. *Tert*-butyl (2*S*,3*R*,4*S*,5*R*)-5-(4-bromophenyl)-3-((*E*)-(((*S*)-*tert*-butylsulfinyl)imino)methyl)-4-phenylpyrrolidine-2-carboxylate

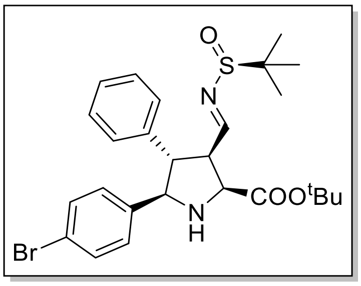

White solid; **Mp**: 76-77 °C; ***R*F** 0.75 (1:1 AcOEt/Hex); **[α]^{20}D** +147.3 (c 0.53, CH_2Cl_2); **IR** (neat) ν: 2969, 1727, 1616, 1484, 1365, 1079, 1010, 825, 732 cm^{-1}; **^{1}H NMR** (300 MHz, $CDCl_3$) δ 7.82 (d, *J* = 8.1 Hz, 1H), 7.41 – 7.35 (m, 2H), 7.25 – 7.12 (m, 5H), 7.00 (m, 2H), 4.23 (d, *J* = 9.6 Hz, 2H), 3.96 – 3.84 (m, 1H), 3.28 – 3.18 (m, 1H), 1.51 (s, 9H), 0.83 (s, 9H); **^{13}C NMR** (101 MHz, $CDCl_3$) δ 171.7, 166.7, 139.3, 136.7, 131.6, 128.8, 128.6, 127.9, 127.3, 121.6, 83.2, 70.8, 62.5, 57.4, 56.6, 56.2, 28.0, 21.9; **MS** (EI) *m/z*: 475 (M^+-C_4H_9, 3%), 422 (12), 420 (15), 377(11), 375(11), 327(41), 325(40), 243 (86), 241(87), 57 (100); **HRMS** (ESI-TOF): m/z calcd for $C_{22}H_{24}N_2O_3S$ [M^+ -C_4H_9] 396.1508, found 396.1501.

3aj. Benzyl (2*S*,3*R*,4*S*,5*R*)-5-(4-bromophenyl)-4-((*E*)-(((*S*)-*tert*-butylsulfinyl)imino)methyl)-3-phenylpyrrolidine-2-carboxylate

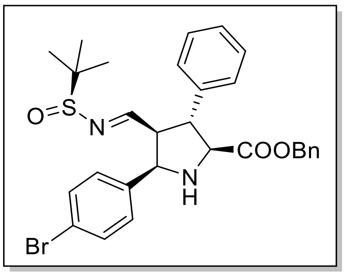

White solid**;** **Mp**: 88-90 °C; ***R*F** 0.70(1:1 AcOEt/Hex); **[α]^{20}D** +76.2 (c 1.08, CH_2Cl_2); **IR** (neat) ν: 1974, 1735, 1619, 1486, 1284, 1186, 1081, 1012, 842, 809, 732, 698 cm^{-1}. **^{1}H NMR** (300 MHz, $CDCl_3$) δ 7.40 – 7.33 (m, 3H), 7.25 – 7.21 (m, 5H), 7.17 (d, *J* = 2.5 Hz, 2H), 7.15 (t, *J* = 2.5 Hz, 3H), 7.08 – 7.03 (m, 2H), 5.15 (d, *J* = 12.4 Hz, 1H), 5.00 (d, *J* = 12.3 Hz, 1H), 4.88 (d, *J* = 7.6 Hz, 1H), 4.17 (d, *J* = 9.0 Hz, 1H), 3.76 – 3.62 (m, 2H), 0.74 (s, 9H). **^{13}C NMR** (101 MHz, $CDCl_3$) δ 172.1, 166.8, 137.9, 137.8, 135.1, 132.1, 128.9, 128.9, 128.6, 128.4, 128.1, 127.6, 122.1, 67.2, 67.1, 64.1, 57.4, 56.6, 51.1, 21.9; **MS** (EI) *m/z*: 511 (M^+-C_4H_7, 13%), 447 (5), 327 (89), 242 (27), 91 (100); **HRMS** (ESI-TOF): m/z calcd for $C_{25}H_{19}BrN_2O_2$ [M^+ -$C_4H_{12}OS$] 458.063, found 460.0586.

3ak. Methyl (2*S*,3*R*,4*S*,5*R*)-5-(4-bromophenyl)-4-((*E*)-(((*S*)-*tert*-butylsulfinyl)imino)methyl)-2-methyl-3-phenylpyrrolidine-2-carboxylate

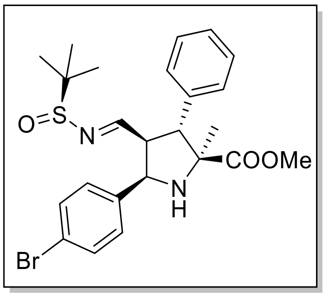

Yellow semi-solid; ***R*F** 0.67 (1:1 AcOEt/Hex); **[α]^{20}D** +93.2 (c 0.50, CH_2Cl_2); **IR** (neat) ν: ν 3343, 1727, 1619, 1484, 1450, 1365, 1257, 1222, 1153, 833 cm^{-1}; **^{1}H NMR** (300 MHz, $CDCl_3$) δ 7.50 – 7.45 (m, 2H), 7.35 – 7.30 (m, 3H), 7.26 (d, *J* = 1.2 Hz, 2H), 7.25 – 7.22 (m, 3H), 4.95 – 4.89 (m, 1H), 4.09 – 4.04 (m, 2H), 3.80 (s, 3H), 1.25 (s, 3H), 0.76 (s, 9H); **^{13}C NMR** (101 MHz, $CDCl_3$) δ 175.5, 168.2, 139.5, 135.7, 131.8, 129.3, 129.0, 128.2, 127.5, 121.7, 68.7, 61.9, 56.5, 53.3, 53.0, 52.5, 22.1, 21.7; **MS** (EI) *m/z*: 325 (M^+-$C_7H_{15}O_3S$, 99%), 281 (18), 257 (97), 255 (100), 195 (53); **HRMS** (ESI-TOF): m/z Calcd for $C_{18}H_{16}BrN_2$ [M+ -$C_6H_{14}O_3S$] 339.0497, found 339.0507.

3ba. Methyl (2*S*,3*R*,4*S*,5*R*)-5-(4-bromophenyl)-4-((*E*)-(((*S*)-*tert*-butylsulfinyl)imino)methyl)-3-(4-chlorophenyl)pyrrolidine-2-carboxylate.

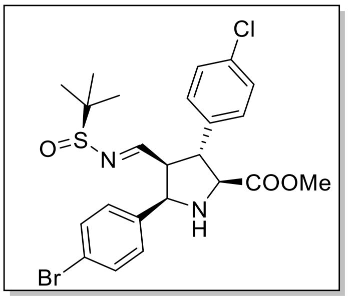

White solid; **Mp**: 116-117°C; ***R*F** 0.66 (1:1 AcOEt/Hex); **[α]^{20}D** +65.1 (c 0.50, CH_2Cl_2); **IR** (neat) ν: 2950, 1743, 1623, 1488, 1203, 1076, 1010, 817, 732 cm^{-1}; **^{1}H NMR** (300 MHz, $CDCl_3$) δ 7.46 (d, *J* = 1.9 Hz, 1H), 7.45 (m, 2H), 7.31 – 7.25 (m, 4H), 7.19 (d, *J* = 8.5 Hz, 2H), 4.88 (d, *J* = 8.1 Hz, 1H), 4.06 (d, *J* = 9.1 Hz, 1H), 3.75– 3.63 (m, 5H), 0.87 (s, 9H); **^{13}C NMR** (101 MHz, $CDCl_3$) δ 173.0, 167.3, 138.8, 137.7, 133.3, 132.0, 129.4, 129.1, 128.9, 122.0, 67.4, 64.1, 57.5, 56.6, 52.5, 50.4, 22.1; **MS** (EI) *m/z*: 419 (M^+-$C_4H_{10}OS$, 4%), 363 (12), 361 (45), 359 (36), 257 (97), 255 (100); **HRMS** (ESI-TOF): m/z Calcd for $C_{17}H_{11}BrN_2$ [M+ -$C_6H_{15}O_3S$], 322.0106 found 322.0089.

3ca. Methyl (2*S*,3*R*,4*S*,5*R*)-5-(4-bromophenyl)-4-((*E*)-(((*S*)-*tert*-butylsulfinyl)imino)methyl)-3-(p-tolyl)pyrrolidine-2-carboxylate

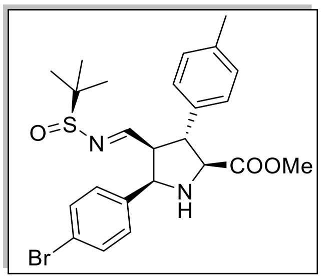

Yellow oil; ***R*F** 0.57 (1:1 AcOEt/Hex); **[α]$^{20}_{D}$** +80.0 (c 0.50, CH_2Cl_2); **IR** (neat) ν: 2954, 1739, 1616, 1515, 1481, 1268, 1180, 914, 690 cm^{-1}. **^{1}H NMR** (300 MHz, $CDCl_3$) δ 7.49 – 7.44 (m, 3H), 7.34 – 7.29 (m, 2H), 7.16 – 7.09 (m, 4H), 4.88 (d, *J* = 7.5 Hz, 1H), 4.10 (d, *J* = 8.7 Hz, 1H), 3.78 – 3.67 (m, 5H), 2.31 (s, 3H), 0.87 (s, 9H); **^{13}C NMR** (101 MHz, $CDCl_3$) δ 173.3, 167.7, 138.7, 137.0, 135.9, 131.9, 129.5, 128.8, 127.7, 121.7, 67.4, 64.1, 57.5, 56.5, 52.3, 50.8, 21.9, 21.06; **MS** (EI) *m/z*: 398 (M^+- $C_4H_{10}OS$ 1%), 341 (50), 339 (53), 257 (96), 255 (100), 197 (49), 195 (51); **HRMS** (ESI-TOF): m/z Calcd for $C_{20}H_{17}BrN_2O_2$ [M+ -$C_4H_{12}OS$] 396.0473, found 396.0467.

3da. Methyl (2*S*,3*R*,4*S*,5*R*)-5-(4-bromophenyl)-4-((*E*)-(((*S*)-*tert*-butylsulfinyl)imino)methyl)-3-(4-methoxyphenyl)pyrrolidine-2-carboxylate

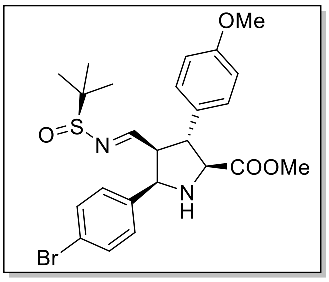

Yellow solid; **Mp**: 83-84 °C; ***R*F** 0.50 (1:1 AcOEt/Hex); **[α]$^{20}_{D}$** +68.0 (c 0.86, CH_2Cl_2); **IR** (neat) ν: 2923, 1731, 1616, 1249, 1176, 1076,825, 725, 686 cm^{-1}; **^{1}H NMR** (400 MHz, $CDCl_3$) δ 7.48 – 7.42 (m, 3H), 7.33 – 7.29 (m, 2H), 7.16 (d, *J* = 8.7 Hz, 2H), 6.83 (d, *J* = 8.7 Hz, 2H), 4.87 (d, *J* = 7.6 Hz, 1H), 4.06 (d, *J* = 8.7 Hz, 1H), 3.77 (s, 3H), 3.72 – 3.66 (m, 5H), 0.86 (s, 9H); **^{13}C NMR** (101 MHz, $CDCl_3$) δ 173.3 , 167.7 , 158.8, 138.9 , 131.9, 130.7, 128.9,128.8, 121.7, 114.2, 67.5, 64.0, 57.6, 56.5, 55.3, 52.3 , 50.51 , 21.9; **MS** (EI) *m/z*: (EI) m/z 357 (M^+-$C_6H_{11}O_3S$, 15%), 355 (18), 281 (10), 257 (95), 255 (100), 226 (15), 224(15); **HRMS** (ESI-TOF): m/z calcd for $C_{24}H_{29}BrN_2O_4S$ [M^+], 520.1031found 520.1097.

3dc. Methyl (2*S*,3*R*,4*S*,5*R*)-5-(2-bromophenyl)-4-((*E*)-(((*S*)-*tert*-butylsulfinyl)imino)methyl)-3-(4-methoxyphenyl)pyrrolidine-2-carboxylate

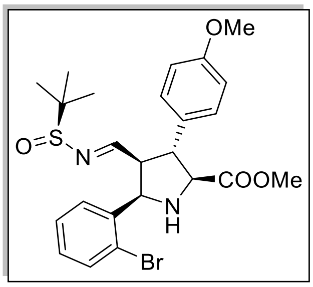

White solid; **Mp**: 48-50 °C; ***R*F** 0.61 (1:1 AcOEt/Hex); **[α]$^{20}_{D}$** +133.9 (c 0.91, CH_2Cl_2); **IR** (neat) ν: 2950, 1739, 1616, 1442, 1365, 1079, 1029, 829, 759 cm^{-1}; **^{1}H NMR** (300 MHz, $CDCl_3$) δ 7.85 (dd, *J* = 7.8, 1.7 Hz, 1H), 7.52 (dd, *J* = 7.9, 1.2 Hz, 1H), 7.39 (d, *J* = 5.8 Hz, 1H), 7.35 (dd, *J* = 7.6, 1.3 Hz, 1H), 7.25 – 7.19 (m, 2H), 7.14 (td, *J* = 7.7, 1.7 Hz, 1H), 6.89 – 6.82 (m, 2H), 5.23 (d, *J* = 7.6 Hz, 1H), 4.05 (d, *J* = 8.5 Hz, 1H), 3.89 – 3.81 (m, 2H), 3.78 (s, 3H), 3.72 (s, 3H), 2.63 (s, 1H), 0.91 (s, 9H); **^{13}C NMR** (101 MHz, $CDCl_3$) δ 173.5 , 167.8, 159.2, 138.8, 133.3, 131.8, 129.8, 129.5, 129.4, 128.3, 123.4, 114.6 , 67.8, 63.6, 56.8 , 55.8, 55.7 , 52.6, 50.2 , 22.5 ; **MS** (EI) *m/z*: 415 (M^+- C_4H_9OS 4%), 355 (12), 341 (25), 281 (42), 207 (100); **HRMS** (ESI-TOF): m/z Calcd for $C_{19}H_{14}BrN_2O_2$ [M+ -$C_5H_{15}O_2S$] 381,0239 found 381.0226.

3bi. *Tert*-butyl (2*S*,3*R*,4*S*,5*R*)-5-(4-bromophenyl)-4-((*E*)-(((*S*)-*tert*-butylsulfinyl)imino)methyl)-3-(4-chlorophenyl)pyrrolidine-2-carboxylate

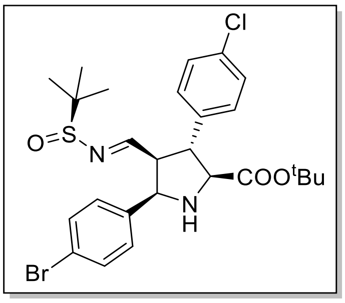

White solid; **Mp**: 62-64 °C; ***R*F** 0.63 (1:1 AcOEt/Hex); **[α]$^{20}_{D}$** +29.2 (c 0.73, CH_2Cl_2); **IR** (neat) ν: 2964, 1729, 1257, 1155, 1076, 1008, 786 cm^{-1}. **^{1}H NMR** (400 MHz, $CDCl_3$) δ 7.48 (d, *J* = 8.5 Hz, 2H), 7.42 (d, *J* = 6.2 Hz, 1H), 7.33 – 7.30 (m, 2H), 7.28 (d, *J* = 8.5 Hz, 2H), 7.19 (d, *J* = 8.5 Hz, 2H), 4.88 (d, *J* = 8.3 Hz, 1H), 3.94 (d, *J* = 9.5 Hz, 1H), 3.71 (ddd, *J* = 9.7, 8.3, 6.2 Hz, 1H), 3.61 (t, *J* = 9.6 Hz, 1H), 1.37 (s, 9H), 0.85 (s, 9H). **^{13}C NMR** (101 MHz, $CDCl_3$) δ 170.5, 166.0, 137.1, 136.6, 133.4, 132.2, 129.5, 128.9, 128.8, 122.5, 83.2, 67.3, 64.0, 57.2, 56.5, 50.6, 27.9, 21.8. **MS** (EI) *m/z*: 511 (M^+-C_4H_7, 17%), 455 (24), 361 (100), 243 (28); **HRMS** (ESI-TOF): m/z calcd for $C_{18}H_{13}BrClN_2O_2$ [M^+ -$C_8H_{19}OS$] 402.9849, found 402.9845.

3di. *Tert*-butyl (2*S*,3*R*,4*S*,5*R*)-5-(4-bromophenyl)-4-((*E*)-(((*S*)-*tert*-butylsulfinyl)imino)methyl)-3-(4-methoxyphenyl)pyrrolidine-2-carboxylate

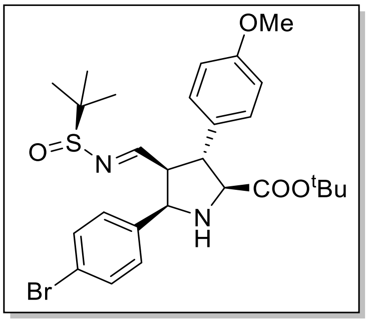

Yellow Solid; **Mp**: 80-82 °C; ***R*F** 0.61 (1:1 AcOEt/Hex); **[α]$^{20}_{D}$** +50.0 (c 1.00 , CH_2Cl_2); **IR** (neat) ν: 2975, 1731, 1616, 1513, 1457, 1390, 1365, 1247, 1155, 1078, 827 cm^{-1}. **^{1}H NMR** (300 MHz, $CDCl_3$) δ 7.42 (d, *J* = 8.5 Hz, 2H), 7.33 (d, *J* = 6.4 Hz, 1H), 7.24 (d, *J* = 8.4 Hz, 2H), 7.10 (d, *J* = 8.7 Hz, 2H), 6.77 (d, *J* = 8.7 Hz, 2H), 4.89 (d, *J* = 8.1 Hz, 1H), 3.99 (d, *J* = 9.6 Hz, 1H), 3.73 – 3.66 (m, 4H), 3.54 (t, *J* = 9.7 Hz, 1H), 1.30 (s, 9H), 0.76 (s, 9H). **^{13}C NMR** (101 MHz, $CDCl_3$) δ 170.3, 165.9, 159.2, 136.2, 132.3, 131.6, 129.1, 128.8, 122.6, 114.2, 83.4, 63.9, 57.01, 56.6, 55.3, 50.7, 27.9, 21.8.**MS** (EI) *m/z*: 507 (M^+-C_4H_7, 23%), 451 (19), 355 (100), 243 (49); **HRMS** (ESI-TOF): m/z calcd for $C_{19}H_{16}BrN_2O_3$ [M^+ -$C_8H_{19}OS$] 399.0344, found 402.0361.

3ea. Methyl (2*S*,3*S*,4*S*,5*R*)-5-(4-bromophenyl)-4-((*E*)-(((*S*)-*tert*-butylsulfinyl)imino)methyl)-3-methylpyrrolidine-2-carboxylate.

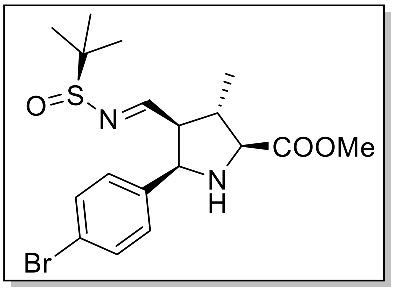

Yellow oil; ***R*F** 0.55 (1:1 AcOEt/Hex); **[α]$^{20}_{D}$** + 173.8 (c 1.00, CH_2Cl_2); **IR** (neat) ν: 2958, 1735, 1617, 1486, 1363, 1199, 1076, 835, 730 cm^{-1}; **^{1}H NMR (400 MHz, $CDCl_3$) δ** 7.49 (d, *J* = 5.7 Hz, 1H), 7.35 (d, *J* = 8.5 Hz, 2H), 7.19 – 7.16 (m, 2H), 4.61 (d, *J* = 8.1 Hz, 1H), 3.75 (s, 3H), 3.54 (d, *J* = 8.7 Hz, 1H), 3.05 (td, *J* = 8.1, 5.7 Hz, 1H), 2.55 (td, *J* = 8.3, 6.6 Hz, 1H), 1.18 (d, *J* = 6.8 Hz, 3H), 0.95 (s, 9H); **^{13}C NMR (101 MHz, $CDCl_3$) δ** 173.7, 168.2, 139.1, 131.7, 128.8, 121.6, 66.8, 63.5, 57.1, 56.5, 52.3, 40.2, 22.1, 17.5; **MS** (EI) *m/z*: 373 (M^+-C_4H_9, 100%), 371(99), 311(63), 309(65), 257(28), 255(29); **HRMS** (ESI-TOF): m/z Calcd for $C_{12}H_{12}BrN_2$ [M+ -$C_6H_{13}O_3S$] 263.0184 found 263.0173.

3fa. Methyl (2*S*,3*R*,4*S*,5*R*)-5-(4-bromophenyl)-4-((*E*)-(((*S*)-*tert*-butylsulfinyl)imino)methyl)-3-ethylpyrrolidine-2-carboxylate

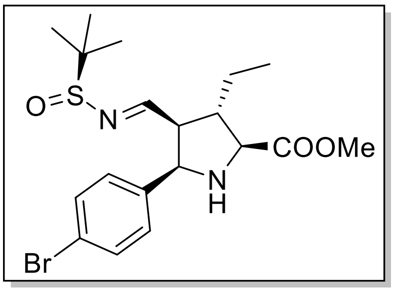

Yellow viscous solid; ***R*F** 0.66 (1:1 AcOEt/Hex); **[α]$^{20}_{D}$** +188.9 (c 1.00, CH_2Cl_2); **IR** (neat) ν: 2958, 1737, 1616, 1486, 1363, 1201, 1076, 1010, 730 cm^{-1}; **^{1}H NMR** (400 MHz, $CDCl_3$) δ 7.60 (d, *J* = 6.1 Hz, 1H), 7.37 (d, *J* = 8.5 Hz, 2H), 7.19 – 7.15 (m, 2H), 4.51 (d, *J* = 7.2 Hz, 1H), 3.73 (s, 3H), 3.60 (d, *J* = 7.2 Hz, 1H), 3.11 (m, 1H), 2.55 – 2.46 (m, 1H), 1.74 – 1.63 (m, 1H), 1.62 – 1.52 (m, 1H), 0.99 (s, 9H), 0.93 (t, *J* = 7.4 Hz, 3H); **^{13}C NMR** (101 MHz, $CDCl_3$) δ 173.5, 169.4, 137. 6, 131.9, 128.8, 121.6, 65.1, 64.0, 56.9, 54.4, 52.4, 47.9, 26.7, 22.4, 12.1. **MS** (EI) *m/z*: (M^+- C_4H_9, 100%), 385(91), 325 (60), 323 (61), 277(25), 195(41); **HRMS** (ESI-TOF): m/z Calcd for $C_{14}H_{15}BrN_2O_2$ [M+ -$C_5H_{12}OS$] 322.0317 found 322.0304.

3ga. Methyl (2*S*,4*S*,5*R*)-5-(2-bromophenyl)-4-((*E*)-(((*S*)-*tert*-butylsulfinyl)imino)methyl)pyrrolidine-2-carboxylate

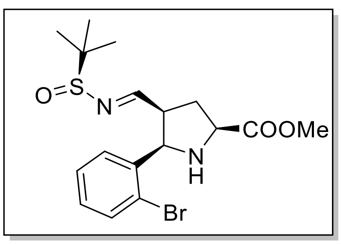

White viscous solid ***R*F** 0.71 (1:1 AcOEt/Hex); **[α]$^{20}_{D}$** +137.2 (c 0.82, CH_2Cl_2); **IR** (neat) ν: 2954, 1735, 1617, 1436, 1211, 1079, 730 cm^{-1}; **^{1}H NMR** (300 MHz, $CDCl_3$) δ 7.70 – 7.65 (m, 1H), 7.57 (d, *J* = 4.8 Hz, 1H), 7.52 (d, *J* = 1.3 Hz, 1H), 7.32 – 7.28 (m, 1H), 7.12 (td, *J* = 7.6, 1.8 Hz, 1H), 4.92 (d, *J* = 7.3 Hz, 1H), 4.04 (t, *J* = 8.3 Hz, 1H), 3.79 (s, 3H), 3.75 – 3.68 (m, 1H), 2.53 – 2.44 (m, 2H), 1.06 (s, 9H); **^{13}C NMR** (101 MHz, $CDCl_3$) δ 174.1, 168.9, 138.4, 133.3, 129.7, 129.1, 128.1, 123.5, 64.0, 58.5, 56.9,

52.6, 46.7, 31.4, 22.7; **MS** (EI) *m/z*: 359 (M^+-C_4H_7, 100%), 357 (89), 309(13), 295 (50), 255(30); **HRMS** (ESI-TOF): m/z Calcd for $C_{13}H_{13}BrN_2O_2$ [M+ -$C_4H_{10}OS$] 308.016, found 308.0135

3ha. Methyl (2*S*,3*R*,4*S*,5*R*)-5-(4-bromophenyl)-4-((*E*)-1-(((*S*)-*tert*-butylsulfinyl)imino)ethyl)-3-phenylpyrrolidine-2-carboxylate

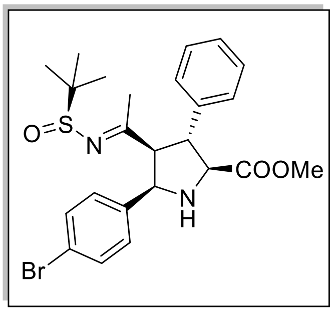

Yellow viscous solid; ***R*$_F$** 0.37 (1:1 AcOEt/Hex)**;** **$[\alpha]^{20}_D$** +4.8 (c 0.50, CH_2Cl_2); **IR** (neat) ν: 2954, 1735, 1612, 1484, 1365, 1068, 1010, 825, 732 cm^{-1}; **^{1}H NMR** (300 MHz, $CDCl_3$) δ 7.48 (d, *J* = 8.5 Hz, 2H), 7.34 – 7.29 (m, 3H), 7.28 – 7.24 (m, 4H), 4.86 (d, *J* = 8.3 Hz, 1H, NHC*H*Ar), 4.02 (d, *J* = 9.1 Hz, 1H), 3.96 – 3.86 (m, 1H), 3.69 (s, 3H), 3.61 (t, *J* = 8.1 Hz, 1H), 1.90 (s, 3H), 1.05 (s, 9H); **^{13}C NMR** (101 MHz, $CDCl_3$) δ 182.3, 173.1 , 140.4 , 138.4, 131.7, 129.2, 128.8, 127.7, 127.7, 127.2, 121.9, 67.8, 65.2, 64.7, 56.5, 52.2, 51.7, 24.4, 22.1; **MS** (EI) *m/z*: 447 (M^+-C_4H_9, 59%), 387 (21), 385 (23), 300 (15), 298 (14), 258 (45), 256 (71), 146 (100). **HRMS** (ESI-TOF): m/z Calcd for $C_{20}H_{20}BrN_2O_2$ [M+ -C_4H_9OS] 399.0708, found 399.07-

3ia. Methyl (2*S*,3*R*,4*S*,5*R*)-5-(4-bromophenyl)-4-((Z)-(((*S*)-*tert*-butylsulfinyl)imino)(phenyl)methyl)-3-phenylpyrrolidine-2-carboxylate

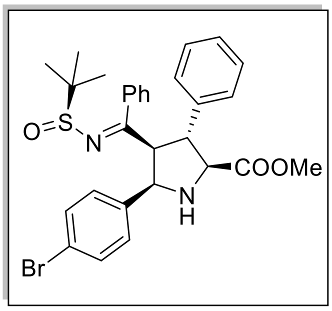

Yellow solid; **Mp**: 49-50 °C; ***R*$_F$** 0.50 (1:1 AcOEt/Hex); **$[\alpha]^{20}_D$** -4.4 (c 0.53, CH_2Cl_2); **IR** (neat) ν: 1739, 1592, 1211, 1072, 701, 663 cm^{-1}; **^{1}H NMR** (400 MHz, $CDCl_3$) δ 7.44 – 7.37 (m, 6H), 7.35 – 7.29 (m, 2H), 7.20 (t, *J* = 7.6 Hz, 2H), 7.05 (d, *J* = 8.3 Hz, 2H), 6.70 – 6.63 (m, 2H), 4.69 (d, *J* = 7.6 Hz, 1H), 4.23 (t, *J* = 6.9 Hz, 2H), 4.03 (d, *J* = 8.6 Hz, 1H), 3.74 (s, 3H), 1.00 (s, 9H); **^{13}C NMR** (101 MHz, $CDCl_3$) δ 184.5 , 172.6, 140.6 , 137.7, 131.5, 129.8, 129.7, 128.9, 127.8, 127.6, 127.4, 126.9, 121.8, 68.5, 65.9, 63.6, 56.0, 54.3, 52.2, 21.7; **MS** (EI) *m/z*: 461 (M^+-$C_4H_{10}OS$, 32%), 459 (40), 448(11), 446(11), 427(33), 425 (31), 208 (100); **HRMS** (ESI-TOF): m/z calcd for $C_{25}H_{22}N_2O_2$ [M+ -C_4H_9BrOS] 382.1681, found 382.1729

5 Transformation reactions

5.1 General procedure for the allylation reaction

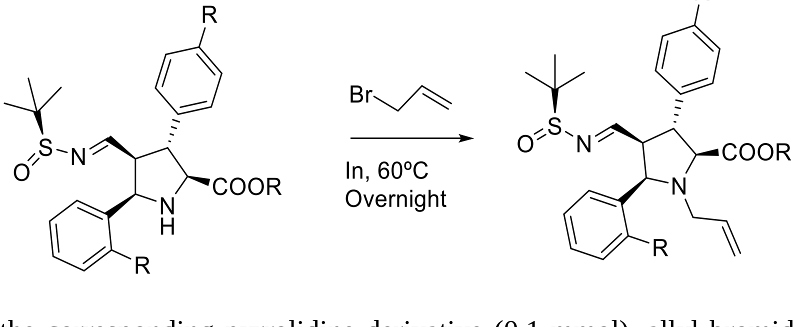

A mixture of the corresponding pyrrolidine derivative (0.1 mmol), allyl bromide (0.15 mmol, 0.182 g, 0.132 mL) and indium metal (0.15 mmol, 0.172 g) in THF (0.5 M, 0.2 mL) was stirred in a high-pressure flask at 60 ºC for 6 h. Then the resulting mixture was hydrolyzed with brine (10 mL) and extracted with EtOAc (3 × 15 mL). The residue was purified by column chromatography (silica gel, hexane/EtOAc). The yields and the physical, spectroscopic, and analytical data follow.

4ac. Methyl (2*S*,3*R*,4*S*,5*R*)-1-allyl-5-(2-bromophenyl)-4-((*E*)-(((*S*)-*tert*-butylsulfinyl)imino)methyl)-3-phenylpyrrolidine-2-carboxylate

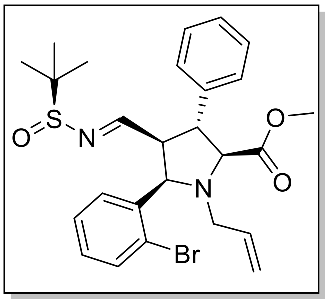

Yield: 26.5 mg, 50 %; Yellow semi-solid; ***R*F** 0.73 (1:1 AcOEt/Hex); **[α]^{20}D** +205.4 (c 0.50, CH_2Cl_2); **IR** (neat) ν: ν 2954, 1619, 1361, 1272, 921, 860, 698 cm^{-1}; **^{1}H NMR** (400 MHz, $CDCl_3$) **δ** 8.06 (dd, *J* = 7.8, 1.8 Hz, 1H), 7.50 (dd, *J* = 7.9, 1.2 Hz, 1H), 7.40 (td, *J* = 7.5, 1.2 Hz, 1H), 7.33 – 7.20 (m, 6H), 7.14 (td, *J* = 7.6, 1.8 Hz, 1H), 5.88 – 5.74 (m, 1H), 5.18 – 5.06 (m, 2H), 4.87 (d, *J* = 8.8 Hz, 1H), 3.86 – 3.79 (m, 2H), 3.76 – 3.66 (m, 1H), 3.65 (s, 3H), 3.37 (dd, *J* = 14.1, 6.4 Hz, 1H), 3.23 (dd, *J* = 14.1, 7.3 Hz, 1H), 0.82 (s, 9H); **^{13}C NMR** (101 MHz, $CDCl_3$) δ 173.5, 167.3, 138.6, 138.1, 133.6, 133.2, 131.0, 129.8, 129.1, 128.4, 128.3, 127.9, 123.8, 119.2, 72.9, 67.9, 56.7, 55.9, 54.7, 52.4, 51.3, 22.42; **MS** (EI) *m/z*: (EI) m/z 367 (M^+-$C_6H_{13}O_3S$ 100%), 365 (99), 245 (7), 217(7), 169(7); **HRMS** (ESI-TOF): m/z Calcd for $C_{20}H_{18}BrN_2$ [M^+ -$C_6H_{15}O_3S$] 365.0653, found 365.0673.

4dc. methyl (2*S*,3*R*,4*S*,5*R*)-1-allyl-5-(2-bromophenyl)-4-((*E*)-(((*S*)-*tert*-butylsulfinyl)imino)methyl)-3-(4-methoxyphenyl)pyrrolidine-2-carboxylate

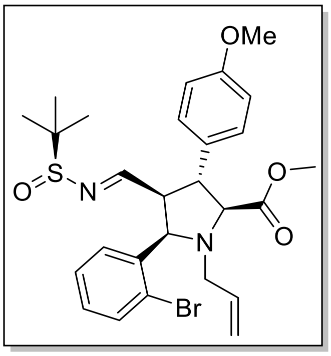

Yield: 24.1 mg, 43 %; Yellow semi-solid**; *R*F 0.70** (1:1 AcOEt/Hex); **[α]^{20}D** +127.7 (c 1.50, CH_2Cl_2); **IR** (neat) ν: 1739, 1616, 1513, 1081, 757 cm^{-1}; **^{1}H NMR** (300 MHz, $CDCl_3$) δ [8.07 (dd, *J* = 7.8, 1.8 Hz, 1H), 7.50 (dd, *J* = 7.9, 1.2 Hz, 1H), 7.41 (dd, *J* = 7.6, 1.3 Hz, 1H), 7.28 (d, *J* = 6.5 Hz, 1H), 7.19 – 7.11 (m, 3H) 6.81 (d, *J* = 8.7 Hz, 2H), ArH and N=CH], 5.88 – 5.72 (m, 1H, CH_2=C*H*), 5.20 – 5.05 (m, 2H, C*H*$_2$=CH), 4.84 (d, *J* = 8.8 Hz, 1H, NHC*H*Ar), 3.80 – 3.74 (m, 5H, OCH_3, NHC*H*CO and NCHC*H*CH), 3.69 – 3.61 (m, 4H, OCH_3, NCHCHC*H*), [3.36 (dd, *J* = 14.0, 6.4, 1.2 Hz, 1H), 3.22 (dd, *J* = 14.1, 7.3 Hz, 1H) NHC*H*$_2$], 0.82 (s, 9H, 3xCH_3); **^{13}C NMR** (101 MHz, $CDCl_3$) δ 173.2(C, *C*=O), 167.0(CH, N=*C*H), 158.93(C, Ar*C*) 138.3(C, Ar*C*), 133.3(CH, Ar*C*), 132.7(CH, Ar*C*), 130.6(CH, Ar*C*), 129.5(C, Ar*C*), 129.3(CH, Ar*C*), 129.0(CH, Ar, Ar*C*), 127.9(CH, Ar*C*), 123.4(C, *C*Br), 118.8(CH_2), 114.1(CH), 72.7(CH, O*C*H$_3$), 67.4(CH, Ar*C*HNH), 56.3(CH_2), 55.6(C, OS*C*(CH_3)$_3$), 55.3(CH, O*C*H$_3$), 54.4(CH, NH*C*HCO), 50.3(CH, *C*HPh), 21.9(CH_3, OSC(*C*H$_3$)$_3$); **MS** (EI) *m/z*: 506 (M^+- C_4H_7 97%), 504 (96), 457 (25), 455 (25), 397 (100); **HRMS** (ESI-TOF): m/z Calcd for $C_{23}H_{23}BrN_2O_2$ [M^+ -$C_4H_{10}OS$] 454.0892, found 454.0867.

5.2 General procedure for the reduction of the imine

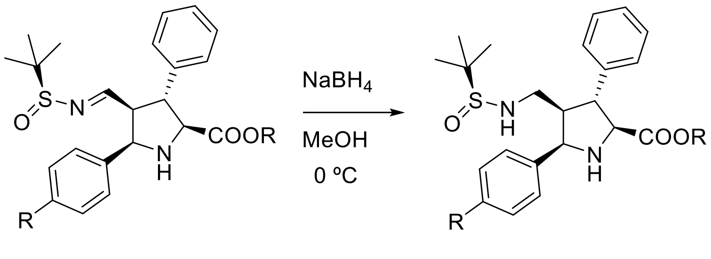

The corresponding pyrrolidine derivative (0.1 mmol) was dissolved in MeOH (0.1 M, 1 mL) and stirred in a vial at 0ºC. After 5 minutes at 0ºC a solution of $NaBH_4$ (0.15 mmol, 5.7 mg) dissolved in 1 mL of MeOH was added dropwise. The reaction was followed by TLC and quenched with NH_4Cl when no starting material was observed and extracted with EtOAc (3 × 15 mL). The yields and the physical, spectroscopic, and analytical data follow.

5aa. Methyl (2*S*,3*R*,4*R*,5*R*)-5-(4-bromophenyl)-4-((((*S*)-*tert*-butylsulfinyl)amino)methyl)-3-phenylpyrrolidine-2-carboxylate

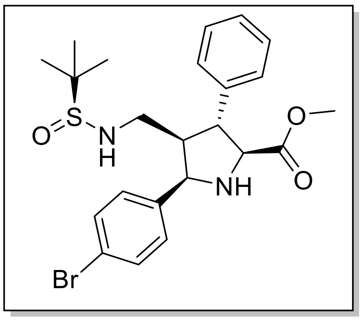

Yield: 49.0 mg, >95 %; White solid; **Mp**: 44-46 °C; $\boldsymbol{R_F}$ 0.50 (2:1 AcOEt/Hex); $[\alpha]^{20}_D$ +6.32 (c 0.81, CH_2Cl_2); **IR** (neat) ν: 2950, 1486, 1444, 1211, 1186, 1054, 1010, 700 cm^{-1}. ^{1}**H NMR** (400 MHz, $CDCl_3$) δ 7.50 (d, *J* = 8.4 Hz, 2H), 7.42 – 7.31 (m, 4H), 7.31 – 7.23 (m, 3H), 4.71 (d, *J* = 7.8 Hz, 1H), 4.03 (d, *J* = 9.5 Hz, 1H), 3.66 (s, 3H), 3.29 (t, *J* = 9.3 Hz, 1H), 2.92 – 2.79 (m, 2H), 2.79 – 2.66 (m, 2H), 0.93 (s, 9H); ^{13}**C NMR** (101 MHz, $CDCl_3$) δ 173.7, 140.6, 139.8, 131.8, 129.3, 128.9, 127.9, 127.4, 121.6, 67.3, 63.5, 55.5, 52.2, 52.1, 52.1, 46.1, 22.3; **MS** (EI) *m/z*: 437 (M^+- C_4H_7 99%), 358 (58), 300 (100), 255(41), 195(48); **HRMS** (ESI-TOF): m/z Calcd for $C_{19}H_{16}BrNO_2$ [M^+ -$C_4H_{13}OS$] 369.0364, found 369.0352.

5dc. Methyl (2*S*,3*R*,4*R*,5*R*)-5-(2-bromophenyl)-4-((((*S*)-*tert*-butylsulfinyl)amino)methyl)-3-(4-methoxyphenyl)pyrrolidine-2-carboxylate

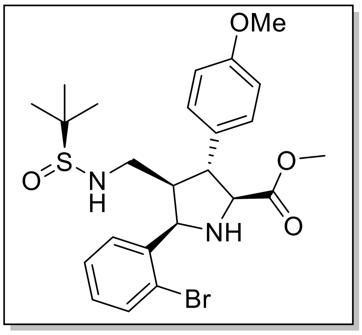

Yield: 52.0 mg, >95 %; White solid; **Mp:** 42-44 °C; $\boldsymbol{R_F}$ 0.4 (2:1 AcOEt/Hex); $[\alpha]^{20}_D$ +43.4 (c 0.78, CH_2Cl_2); **IR** (neat) ν: 2954, 1737, 1612, 1513, 1463, 1249, 1178, 1029, 829, 757 cm^{-1}. ^{1}**H NMR** (400 MHz, $CDCl_3$) δ 7.72 (dd, *J* = 7.8, 1.7 Hz, 1H), 7.50 (dd, *J* = 8.0, 1.3 Hz, 1H), 7.32 (d, *J* = 1.4 Hz, 1H), 7.24 – 7.20 (m, 2H), 7.13 (dd, *J* = 7.7, 1.7 Hz, 1H), 6.85 – 6.80 (m, 2H), 5.14 (d, *J* = 8.5 Hz, 1H), 4.04 (d, *J* = 9.6 Hz, 1H), 3.73 (s, 3H), 3.63 (s, 3H), 3.42 – 3.37 (m, 1H), 3.03 – 2.95 (m, 1H), 2.91 – 2.83 (m, 2H), 2.82 – 2.75 (m, 1H) , 0.87 (s, 9H). ^{13}**C NMR** (101 MHz, $CDCl_3$) δ 173.1, 158.7, 139.6, 133.1, 132.9, 129.1, 129.0, 127.7, 123.4, 114.3, 67.1, 62.7, 55.5, 55.3, 52.1, 51.1, 49.7, 46.3, 22.4; **MS** (EI) *m/z*: 467 (M^+- C_4H_7 100%), 388 (48), 330 (96), 301 (18), 255 (70), 192 (74), 147 (89); **HRMS** (ESI-TOF): m/z Calcd for $C_{20}H_{20}BrN_2O_3$ [M^+ -$C_4H_{11}OS$] 415.0657, found 415.0631.

5ac. Methyl (2*S*,3*R*,4*R*,5*R*)-5-(2-bromophenyl)-4-((((*S*)-*tert*-butylsulfinyl)amino)methyl)-3-phenylpyrrolidine-2-carboxylate

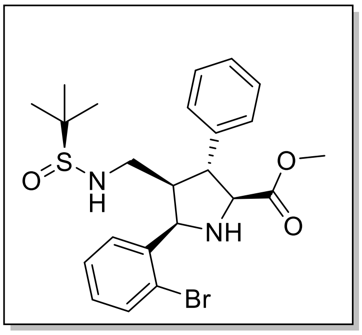

Yield: 49.0 mg, >95 %; White solid; **Mp:** 41-42°C; $\boldsymbol{R_F}$ 0.60 (2:1 AcOEt/Hex); $[\alpha]^{20}_D$ +50.5 (c 1.07, CH_2Cl_2); **IR** (neat) ν: 1737, 1457, 1436, 1207, 1051, 1024, 754, 700 cm^{-1}. ^{1}**H NMR** (400 MHz, $CDCl_3$) δ 7.76 (dd, *J* = 7.8, 1.8 Hz, 1H), 7.48 (dd, *J* = 7.9, 1.3 Hz, 1H), 7.31 – 7.26 (m, 5H), 7.21 – 7.17 (m, 1H), 7.09 (td, *J* = 7.6, 1.7 Hz, 1H), 4.98 (d, *J* = 8.3 Hz, 1H), 3.93 (d, *J* = 9.0 Hz, 1H), 3.62 (s, 3H), 3.39 (dd, *J* = 9.1, 7.3 Hz, 1H), 3.01 – 2.92 (m, 2H), 2.88 – 2.80 (m, 1H), 2.79 – 2.70 (m, 1H) , 0.89 (s, 9H 3).^{13}**C NMR** (101 MHz, $CDCl_3$) δ 173.0 , 141.4 , 139.5, 132.9, 129.1, 129.0, 128.9, 128.1, 127.7, 127.2, 123.4, 67.1, 62.9, 55.5, 52.0, 51.8, 49.7, 46.4, 22.5 ; **MS** (EI) *m/z*: 493 (M^+ >5%), 437 (100), 435 (89), 300(53), 298(51); **HRMS** (ESI-TOF): m/z Calcd for [M^+ -$C_4H_{13}NOS$] 369.0364, found 369.0367.

5.3 General procedure for intramolecular cyclization

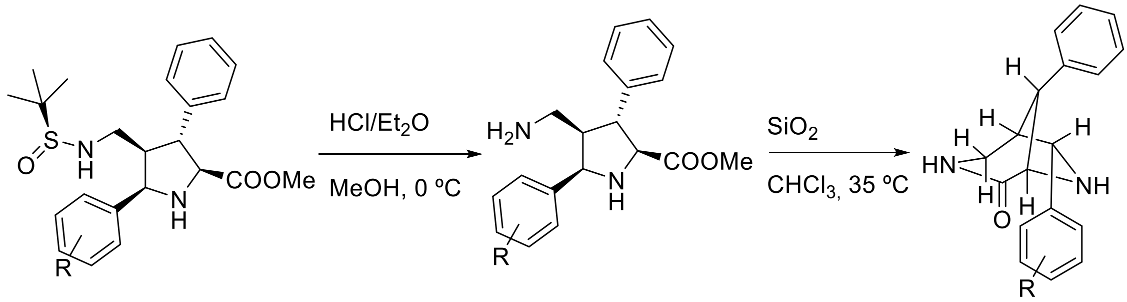

The corresponding pyrrolidine reducted derivative (0.1 mmol) was dissolved in MeOH (0.1 M, 1 mL) and stirred in a vial at 0ºC. After 5 minutes at 0ºC a solution of HCl/Et_2O 2M (0.5 mmol, 0.25 mL) was slowly added. The reaction was stirred for 30 min. After, 5 mL of AcOEt were added at the same temperature and finally the reaction was quenched with NaOH 2M (0.5 mmol, 0.25 mL) at 0ºC. After few seconds of stirring, the reaction was transfer to a decantation funnel and the organic layer was isolated and dried with $MgSO_4$. Solvents were evaporated. The intermediated obtained reacted for 24 h with a spoon of SiO_2 in $CHCl_3$ (0.1 M, 1 mL) for 24 h at 35 ºC in a sand bath. The product was filter using 5 mL of AcOEt and solvents were evaporated.

6ac. (5*R*,7*S*,8*R*)-7-(2-bromophenyl)-8-phenyl-3,6-diazabicyclo[3.2.1]octan-4-one

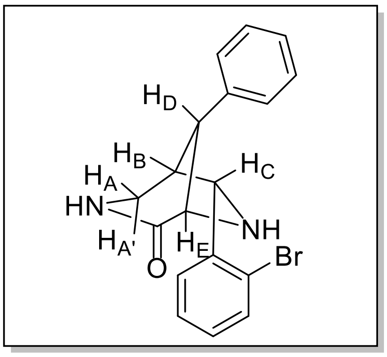

Yield: 35.0 mg >95 %; Beige solid; ***R*F** 0.40 (2:1 AcOEt/Hex); **$[\alpha]^{20}_D$** +8.14 (c 1.50, CH_2Cl_2)**; IR** (neat) ν: 1677, 1494, 1332, 1022, 748, 719, 696 cm^{-1}. **^1H NMR** (400 MHz, $CDCl_3$) δ [7.96 (dd, *J* = 7.8, 1.7 Hz, 1H), 7.54 – 7.44 (m, 5H), 7.37 – 7.31 (m, 2H), 7.19 – 7.07 (m, 1H) Ar*H*], 5.38 (s, 1H, N*H*), 4.95 (d, *J* = 5.4 Hz, $1H_C$), 4.13 (d, *J* = 2.9 Hz, $1H_E$), 3.71 (s, $1H_D$), 3.65 – 3.59 (m, $1H_B$), 3.33 (dd, *J* = 11.6, 4.2 Hz, $1H_A$), 2.65 (d, *J* = 11.7 Hz, $1H_A$). **^{13}C NMR** (101 MHz, $CDCl_3$) δ 132.6 (CH, Ar*C*), 132.2(C, Ar*C*), 132.0(C, Ar*C*), 129.4(CH, Ar*C*), 129.1(CH, Ar*C*), 129.0(CH, Ar*C*), 127.7(CH, Ar*C*), 127.4(CH, Ar*C*), 126.5(CH, Ar*C*), 122.9 (C, *C*Br), 63.7(CH, CO*C*HNH), 62.5(CH, ArCHNH), 48.6(CH_2, $NHCH_2$*C*H), 43.3(CH_2, NH*C*H_2), 40.4 (CH, *C*HPh). **MS** (EI) *m/z*: 358 (M^+ 8%), 356 (9), 313 (78), 311 (77), 277 (100); **HRMS** (ESI-TOF): m/z Calcd for $C_{18}H_{17}BrN_2O$ [M^+] 356.0524, found 356.053.

6aa. (5*R*,7*S*,8*R*)-7-(4-bromophenyl)-8-phenyl-3,6-diazabicyclo[3.2.1]octan-4-one

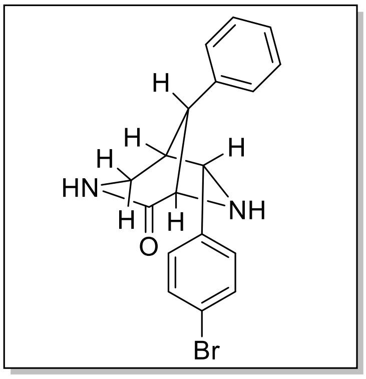

Yield: 35.1 mg >95 %; Beige solid; ***R*F** 0.35 (2:1 AcOEt/Hex); **$[\alpha]^{20}_D$** +31.9 (c 1.20, CH_2Cl_2);**IR** (neat) ν: 1675, 1490, 1332, 1263, 1076, 796, 698 cm^{-1}. **^1H NMR** (400 MHz, $CDCl_3$) δ 8.10 (d, *J* = 1.5 Hz, 1H), 7.46 – 7.43 (m, 6H), 7.32 (d, *J* = 8.3 Hz, 2H), 5.91 – 5.78 (m, 1H), 4.70 (s, 1H), 4.61 (d, *J* = 5.3 Hz, 1H), 4.29 – 4.15 (m, 1H), 3.45 – 3.32 (m, 1H), 2.98 – 2.93 (m, 1H), 2.89 – 2.82 (m, 1H). **^{13}C NMR** (101 MHz, $CDCl_3$) δ 131.6, 129.2, 128.8, 127.5, 126.39, 62.4 , 61.7 , 48.6, 44.8, 43.0; **MS** (EI) *m/z*: 358 (M^+ 17%), 356 (20), 313 (88), 311 (89), 300 (98), 298(100); **HRMS** (ESI-TOF): m/z Calcd for $C_{18}H_{17}BrN_2O$ [M^+] 356.0522, found 356.0524.

6 Isomerization experiments

A mixture of the *N*-*tert*-butanesulfinyl imine **1a** (70.6 mg, 0.3 mmol), α-imino ester **2d** (127 mg, 0.6 mmol) and Silver Carbonate (10 mol %, 8.3 mg) in toluene (0.4 M, 0.75 mL) was stirred in a round bottom vial at room temperature and monitorized by H-RMN at different reaction times.

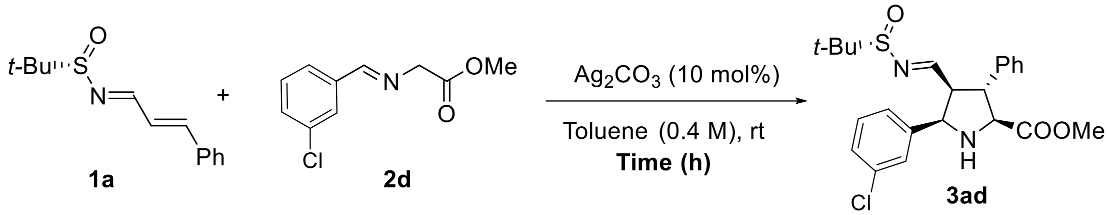

Table S4. Relation between time, conversion and diastereomerical ratio for product **3ad**.

Entry	Time (h)	Conv. (%)	dr
1	8	40	80:20
2	48	>95	71:29
3	96	>95	59:41

Remarkably, when the reaction was carried out for 4 days, the diastereomeric ratio changed to 59:41. This allowed us to confirm that epimerisation of a centre occurred. For this reason, it was studied whether adding a stronger base would produce this epimerization.

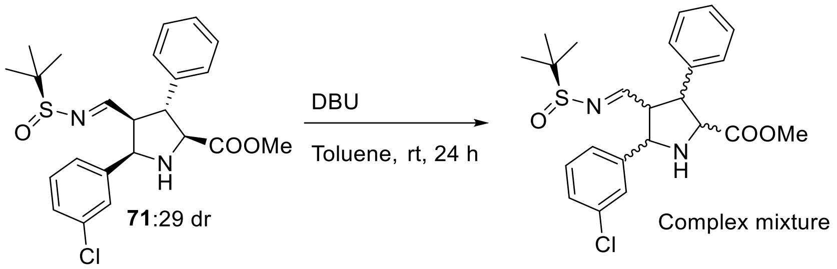

A mixture of the corresponding crude product **3ad** (0.05 mmol), DBU (0.1 mmol) and toluene (0.25 mL) was stirred in a round bottom vial at room temperature and monitorized by ^{1}H-NMR. After 24 hour of reaction a huge mixture of products was observe, proving the epimerization of the reaction products.

7 Aldol reaction

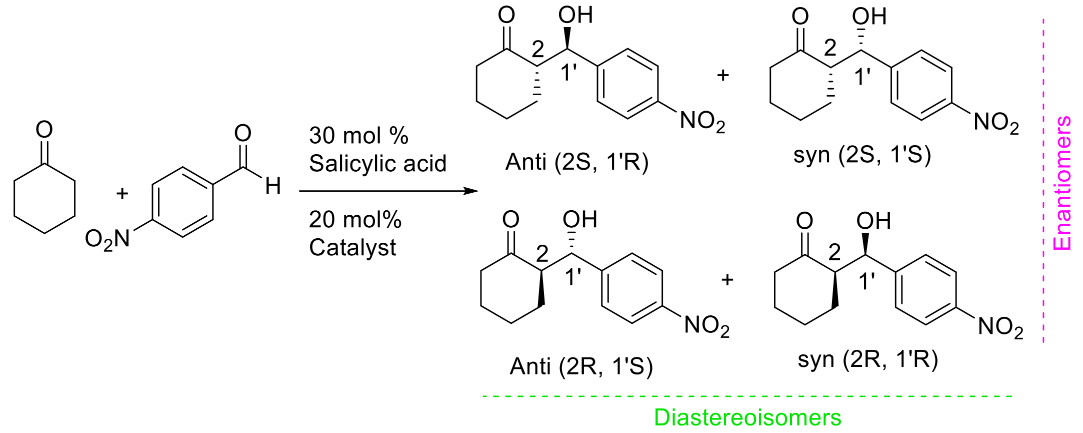

A mixture of 4-nitrobenzaldehyde **8** (0.125 mmol, 18.9 mg), salicylic acid (0.0375 mmol, 5.2 mg), the product **5aa** (0.025 mmol, 12.2 mg) and cyclohexanone **7** (7.25 mmol, 0.75 mL) as solvent and reagent, were stirred in a round bottom vial at room temperature for 24 hours. The diastereomeric relation was determined by ^{1}H-RMN 78:22. The enantiomerical excess was determined by HPLC using a chiralpak AD-H column (hexane/iPrOH = 80/20, 1 mL/min). For the major diastereoisomer (anti), the enantiomeric relation was (68:22) tR (13.987 min: 18.040 min). For the minor diastereoisomer (syn) the enantiomeric relation was (75:25) tR (13.160 min: 11.227 min).[8,9,10]

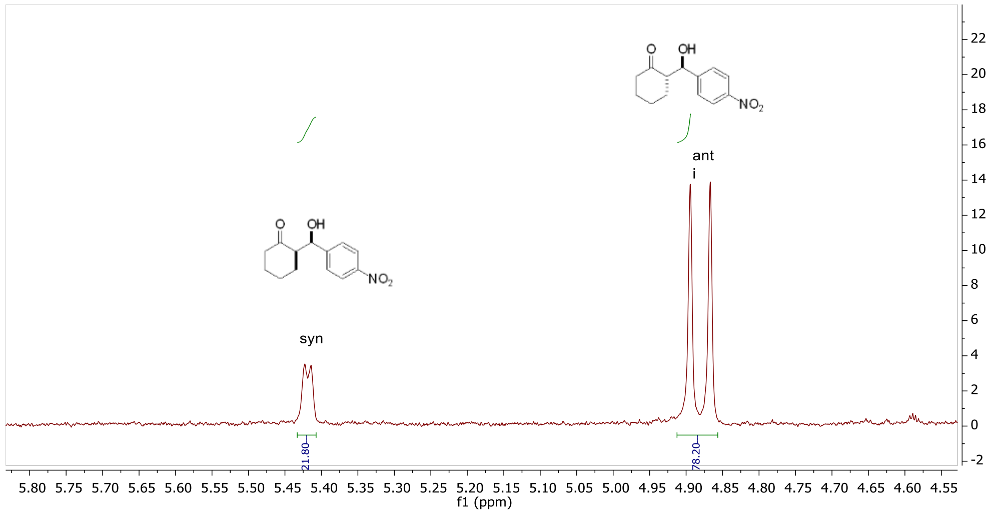

[8] Lombardo, M.; Easwar, S.; Pasi, F. and Trombini, C. The ion tag strategy as a route to highly efficient organocatalysts for the direct asymmetric aldol reaction. *Adv. Synth. Catal.* **2009**, *351,* 276 – 282

[9] Zhang, N. and Wu, C. Tailoring Protein–Polymer Conjugates as Efficient Artificial Enzymes for Aqueous Asymmetric Aldol Reactions. *ACS Synth. Biol.* **2022**, *11,* 3797–3804

[10] Zhou, P.; Lou, S. and Cheng, J-P. Highly enantioselective synthesis of syn-aldols of cyclohexanones via chiral primary amine catalyzed asymmetric transfer aldol reactions in ionic liquid. *Org. Biomol. Chem.* **2011,** *9,* 1784

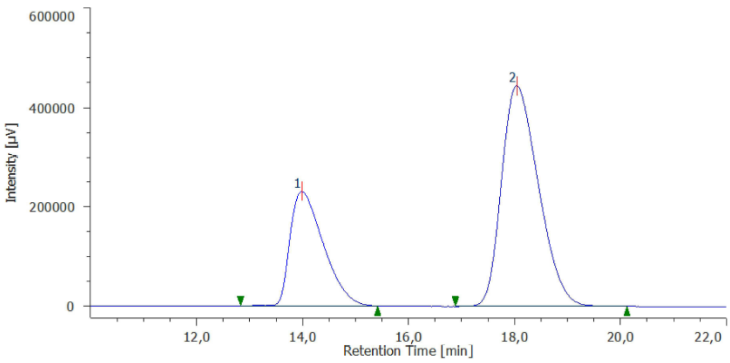

#	Peak Name	CH	tR [min]	Area [μV·sec]	Height [μV]	Area%	Height%
1	Unknown	9	13,987	10082798	230263	31,879	34,175
2	Unknown	9	18,040	21545543	443505	68,121	65,825

Diastereoisomer *anti*(2*R*, 1'*S*)

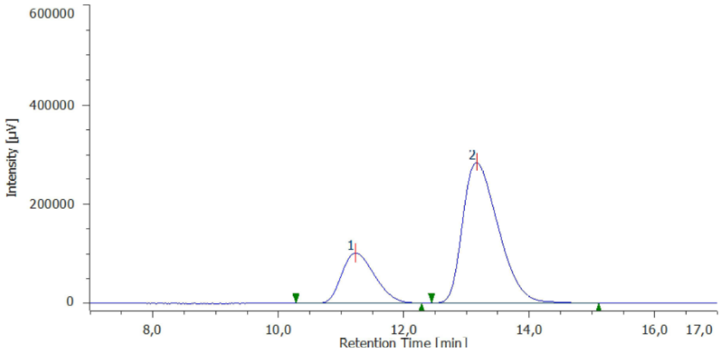

#	Peak Name	CH	tR [min]	Area [μV·sec]	Height [μV]	Area%	Height%
1	Unknown	9	11,227	3692702	100994	24,848	26,259
2	Unknown	9	13,160	11168347	283610	75,152	73,741

Diastereoisomer syn(2*S*, 1'*S*)

8 NMR spectra

Methyl (2*S*,3*R*,4*S*,5*R*)-5-(4-bromophenyl)-4-((*E*)-(((*S*)-*tert*-butylsulfinyl)imino)methyl)-3-phenylpyrrolidine-2-carboxylate. ^{1}H NMR (400 MHz, $CDCl_3$) of 3aa

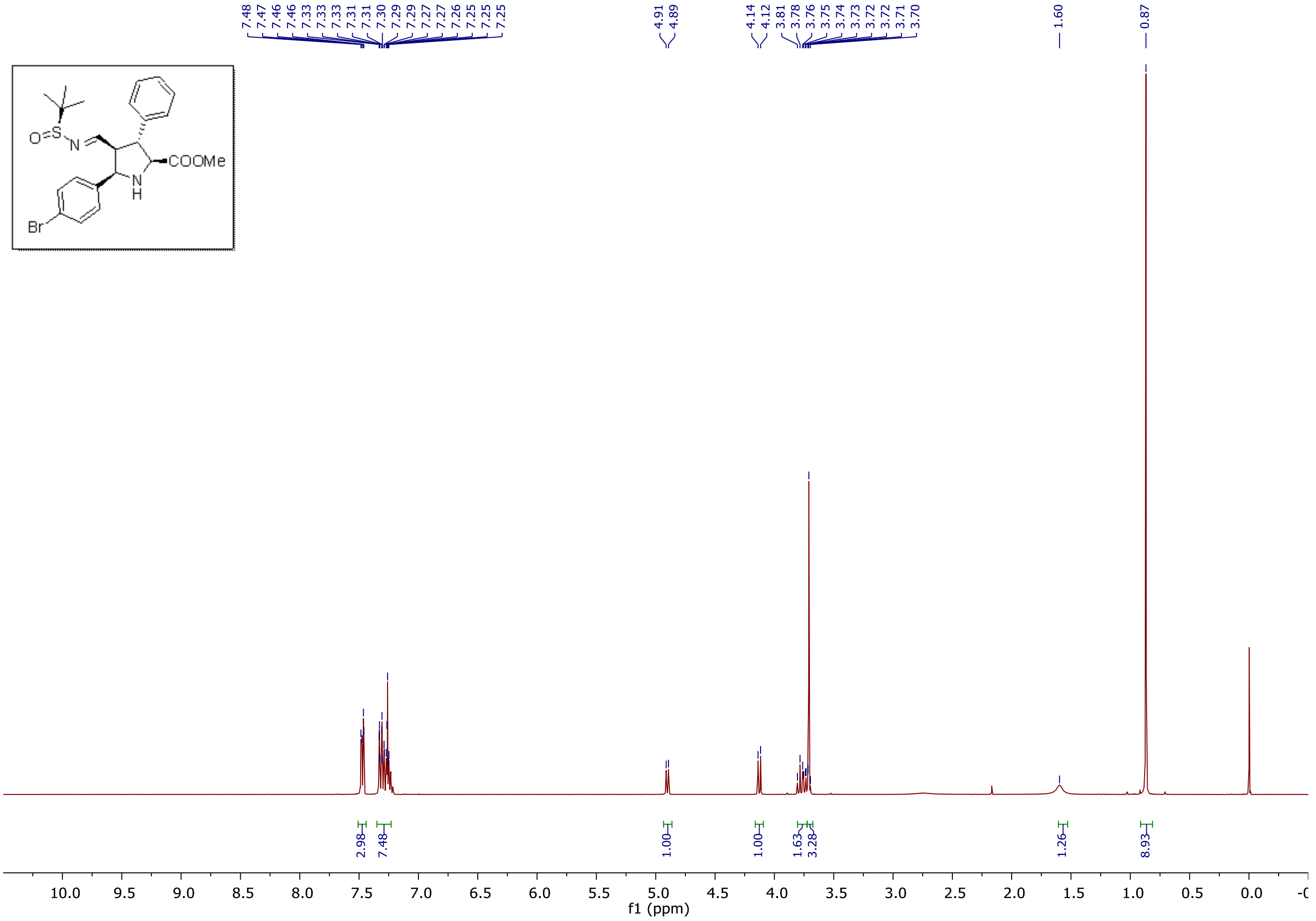

^{13}C{H} NMR (101 MHz, $CDCl_3$) of 3aa

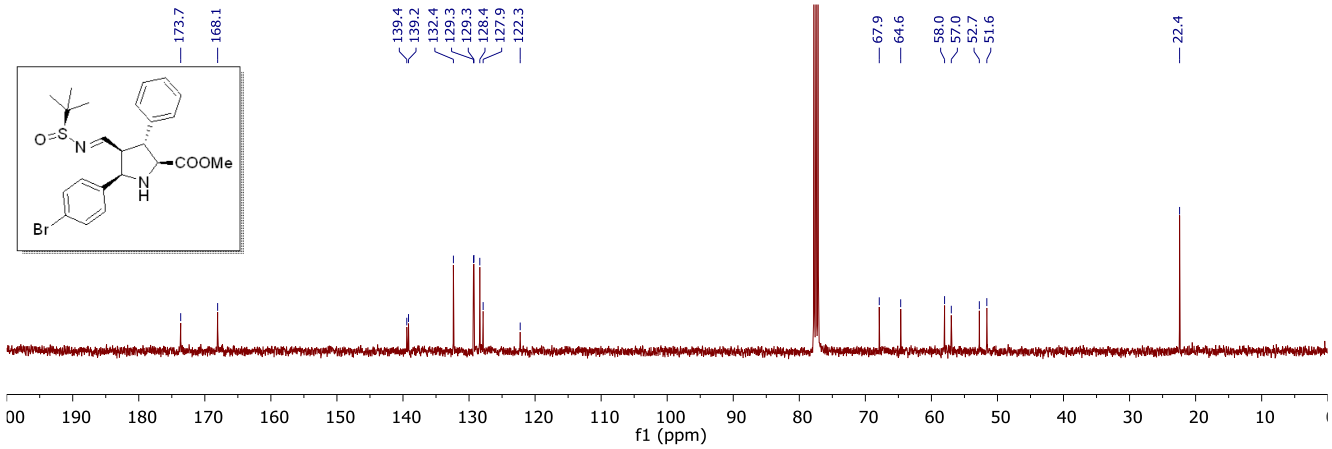

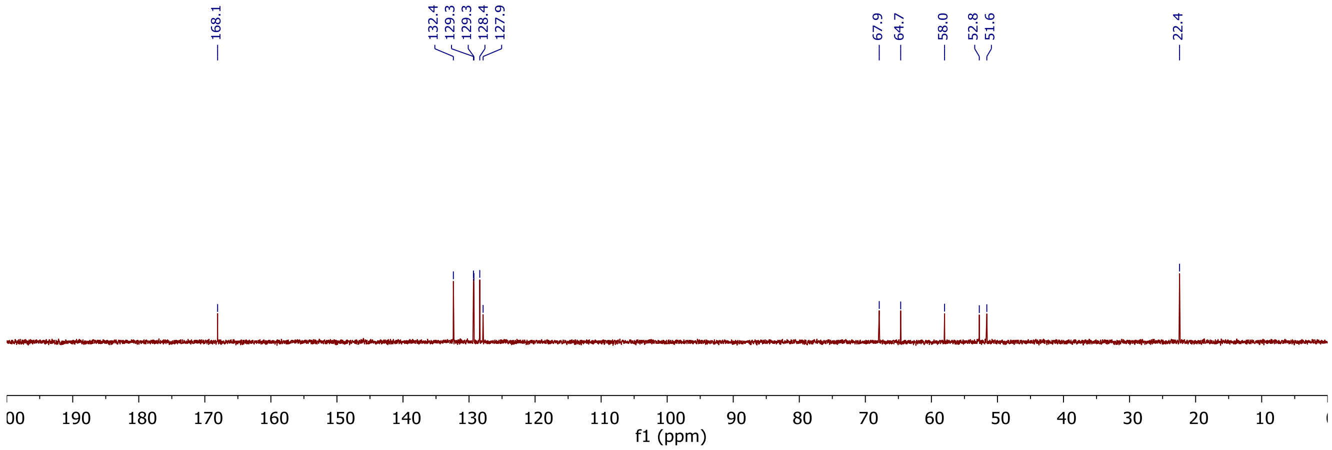

COSY ($CDCl_3$) of 3aa

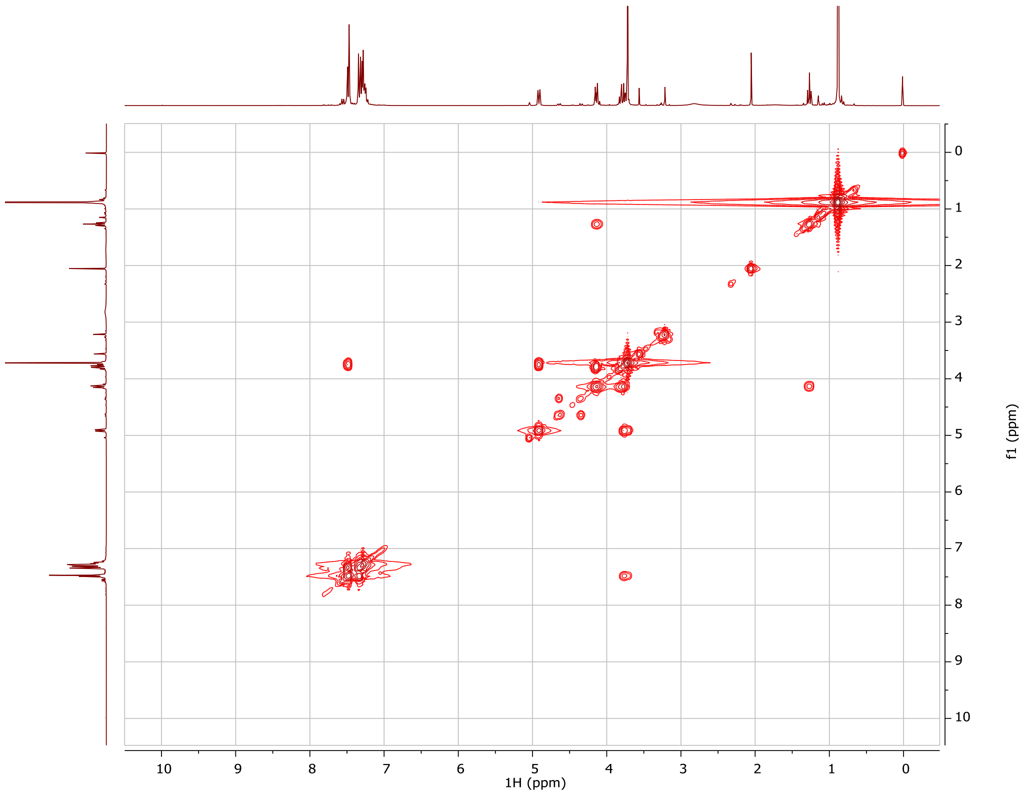

HSQC ($CDCl_3$) of 3aa

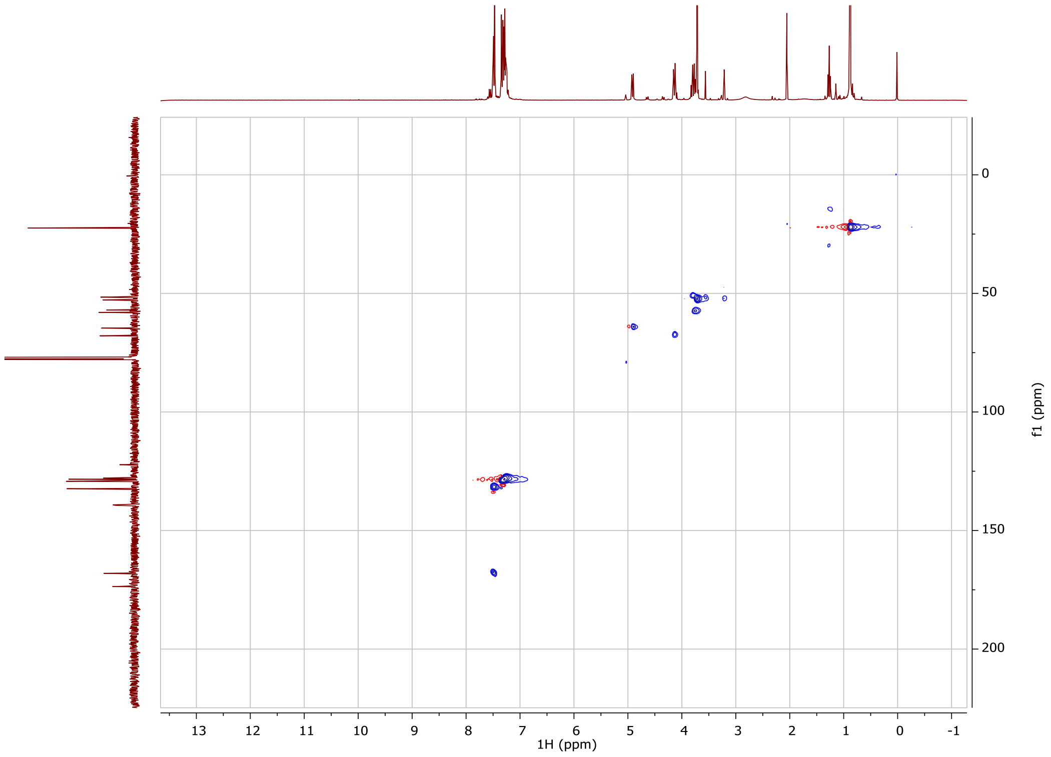

Methyl (2*S*,3*R*,4*S*,5*R*)-5-(4-bromophenyl)-4-((*E*)-(((*R*)-*tert*-butylsulfinyl)imino)methyl)-3-phenylpyrrolidine-2-carboxylate. ^{1}**H NMR (500 MHz, CDCl$_3$) of ent3aa**

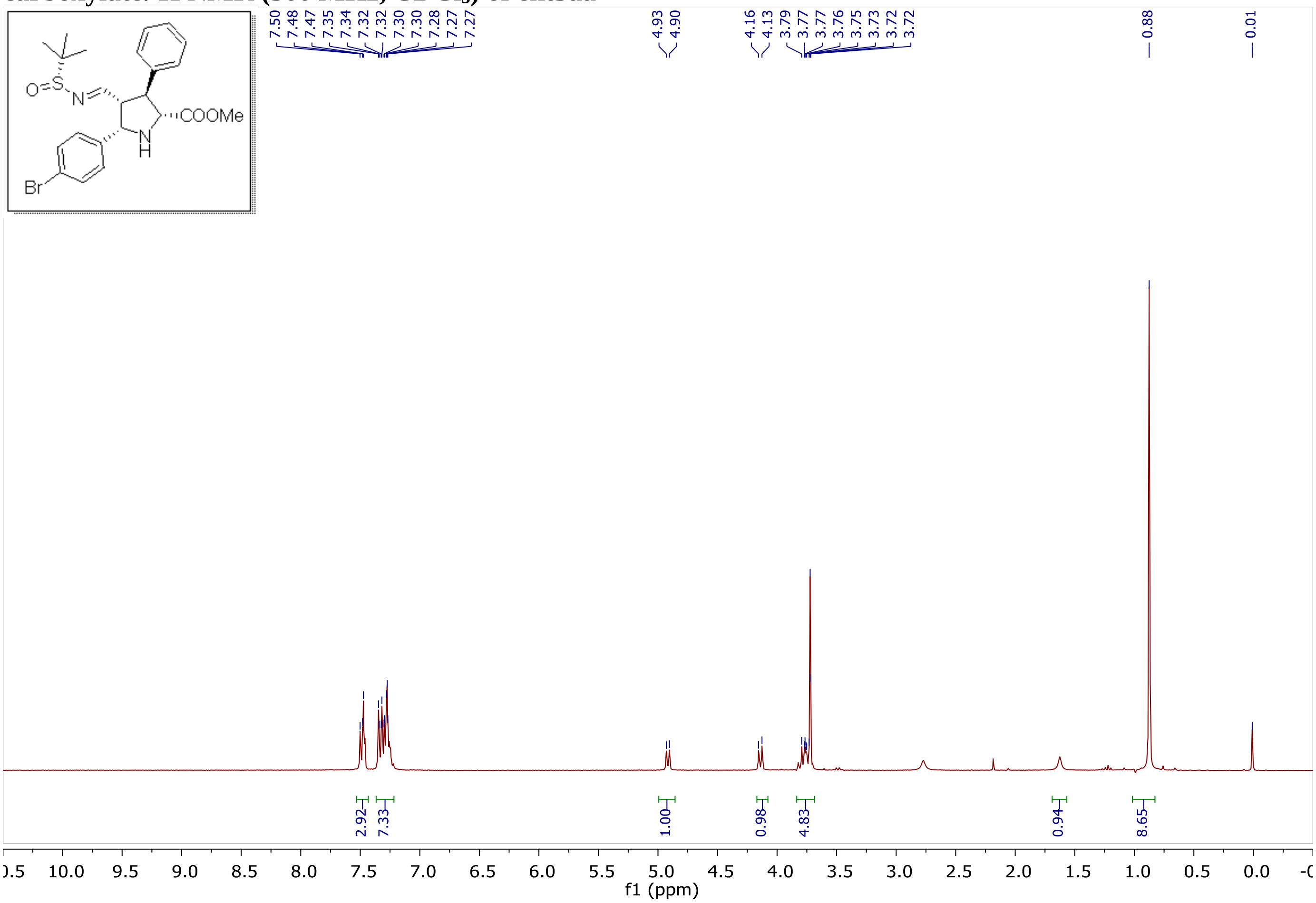

^{13}**C{H} NMR (101 MHz, CDCl$_3$) of 3aa'**

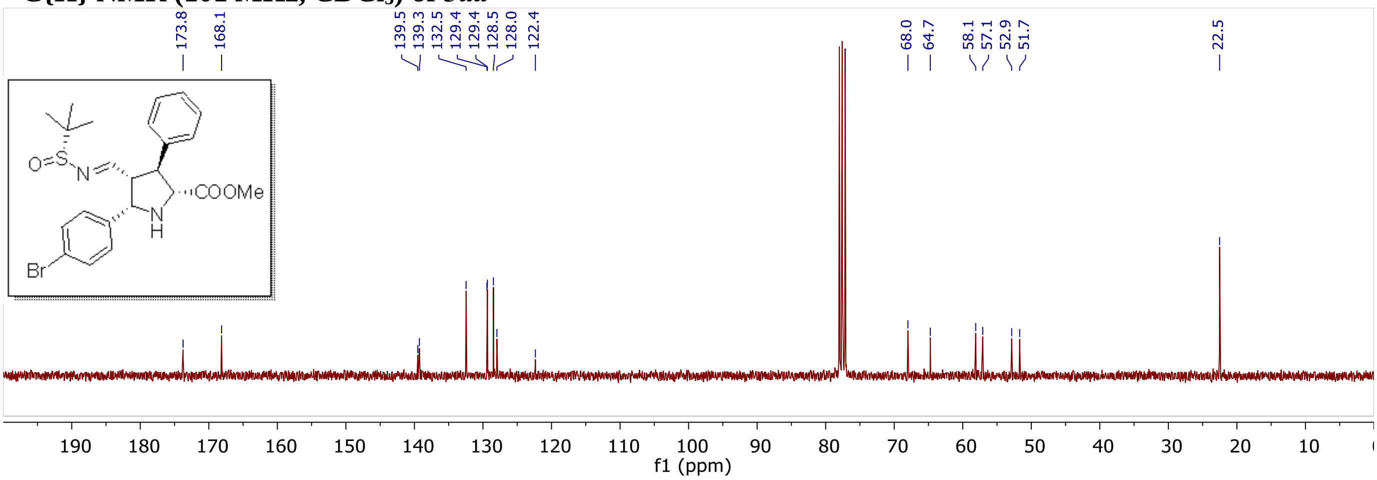

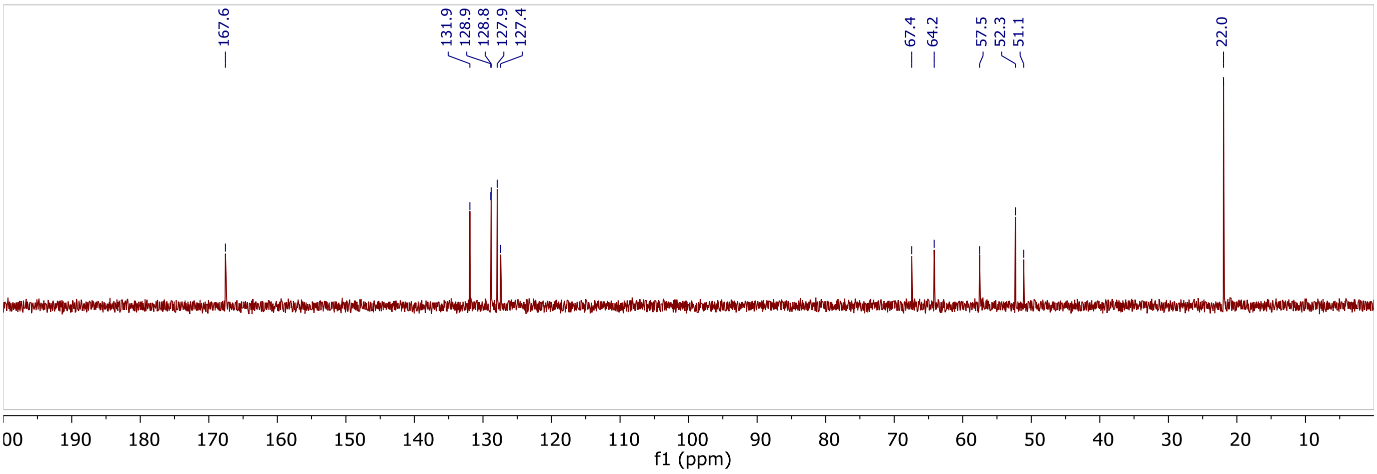

Methyl (2*S*,3*R*,4*S*,5*R*)-4-((*E*)-(((*S*)-*tert*-butylsulfinyl)imino)methyl)-3,5-diphenylpyrrolidine-2-carboxylate. ^{1}H NMR (300 MHz, $CDCl_3$) of 3ab

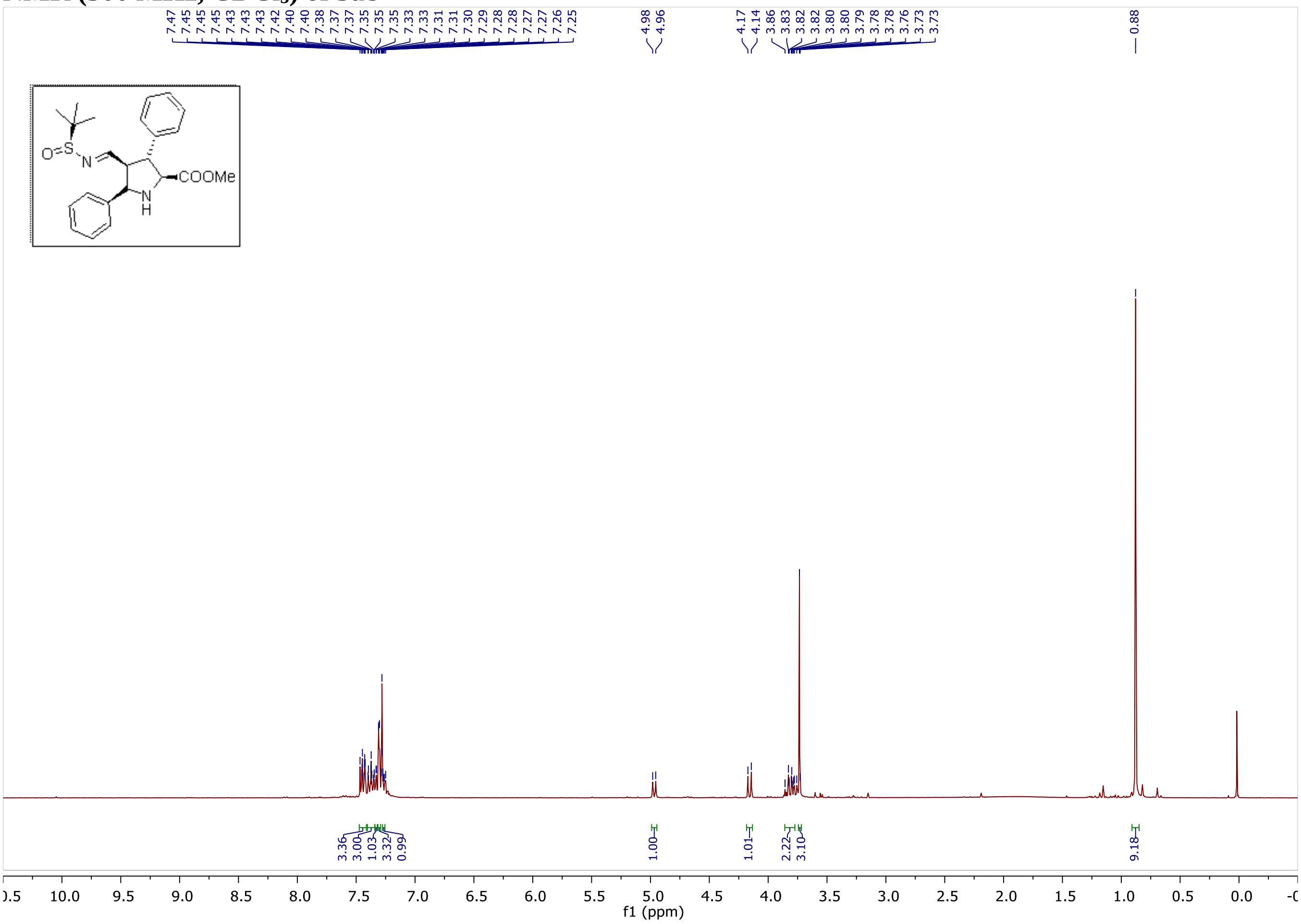

^{13}C{H} NMR (101 MHz, $CDCl_3$) of 3ab

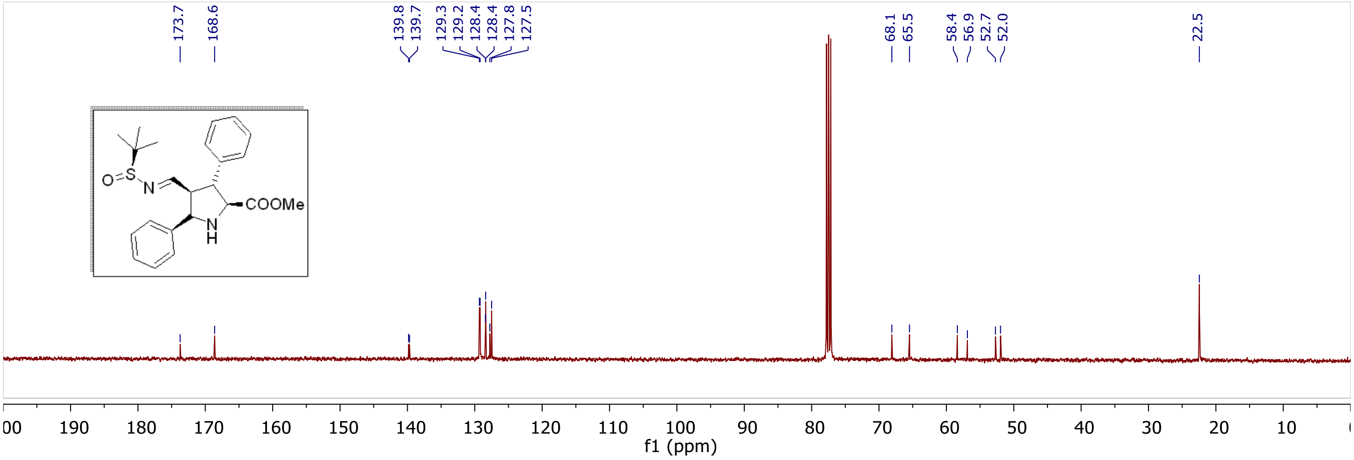

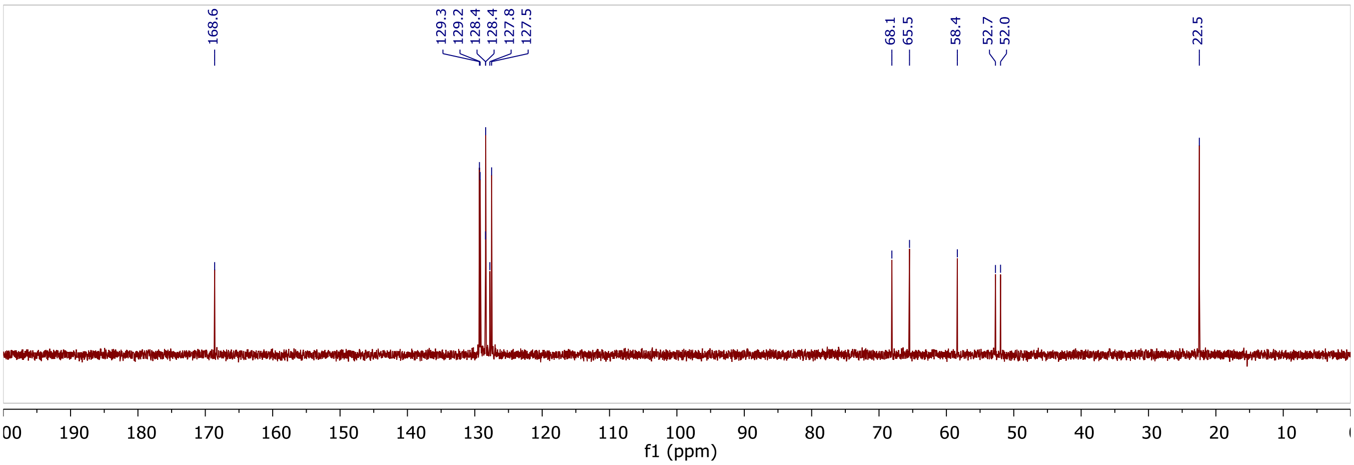

Methyl (2*S*,3*R*,4*S*,5*R*)-5-(2-bromophenyl)-4-((*E*)-(((*S*)-*tert*-butylsulfinyl)imino)methyl)-3-phenylpyrrolidine-2-carboxylate. ^{1}H NMR (300 MHz, $CDCl_3$) of 3ac

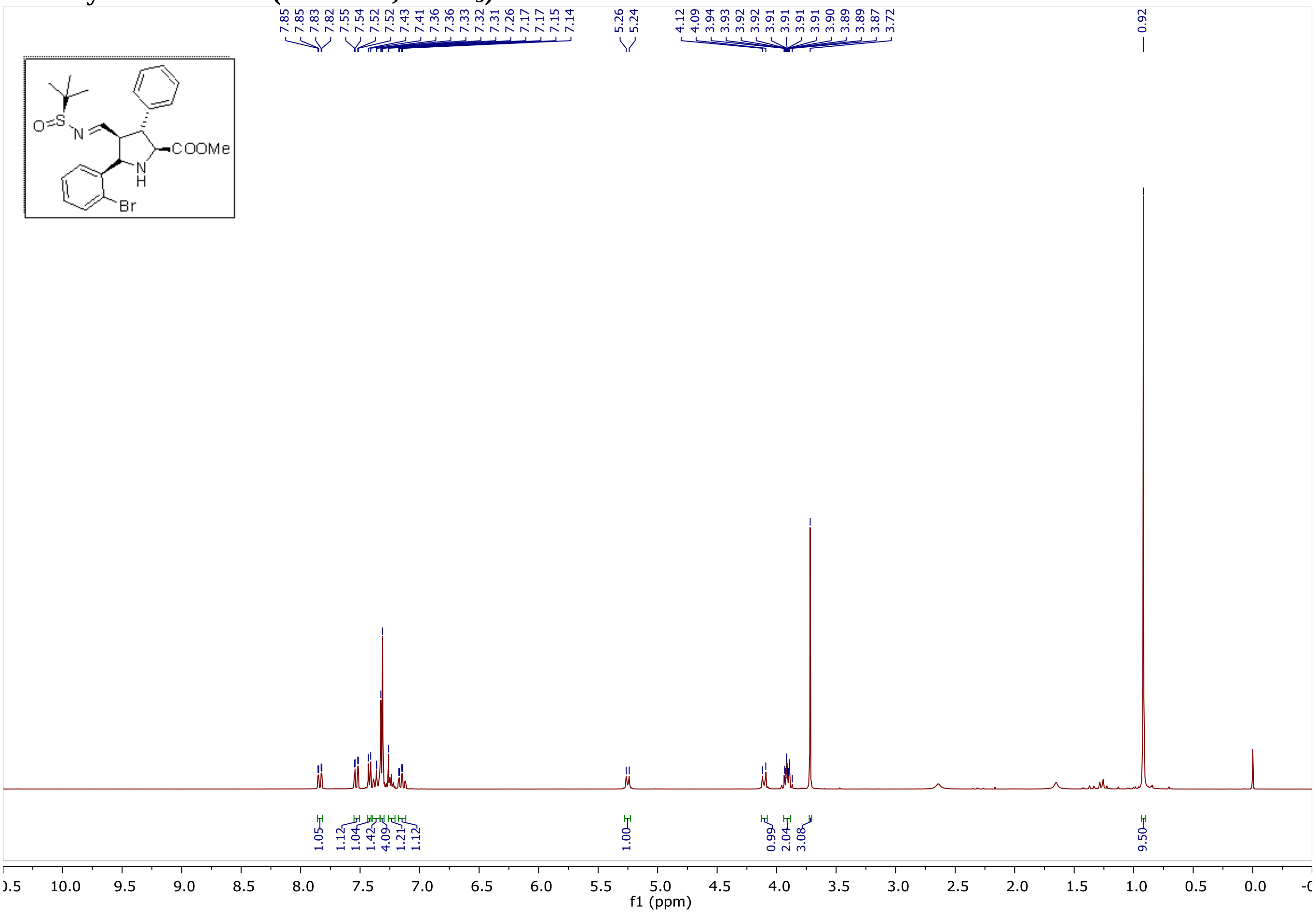

$^{13}C\{H\}$ NMR (101 MHz, $CDCl_3$) of 3ac

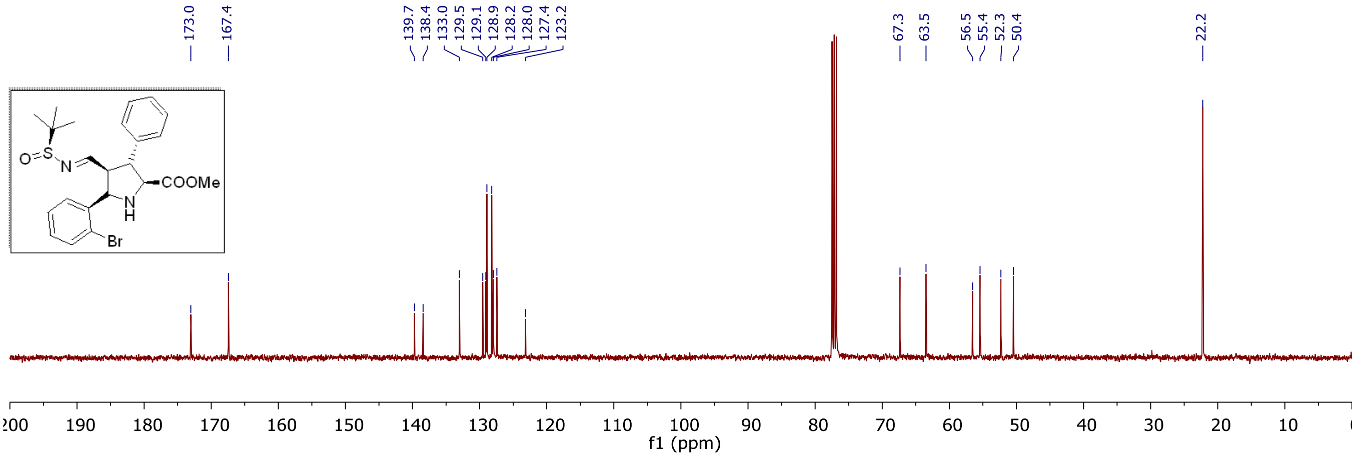

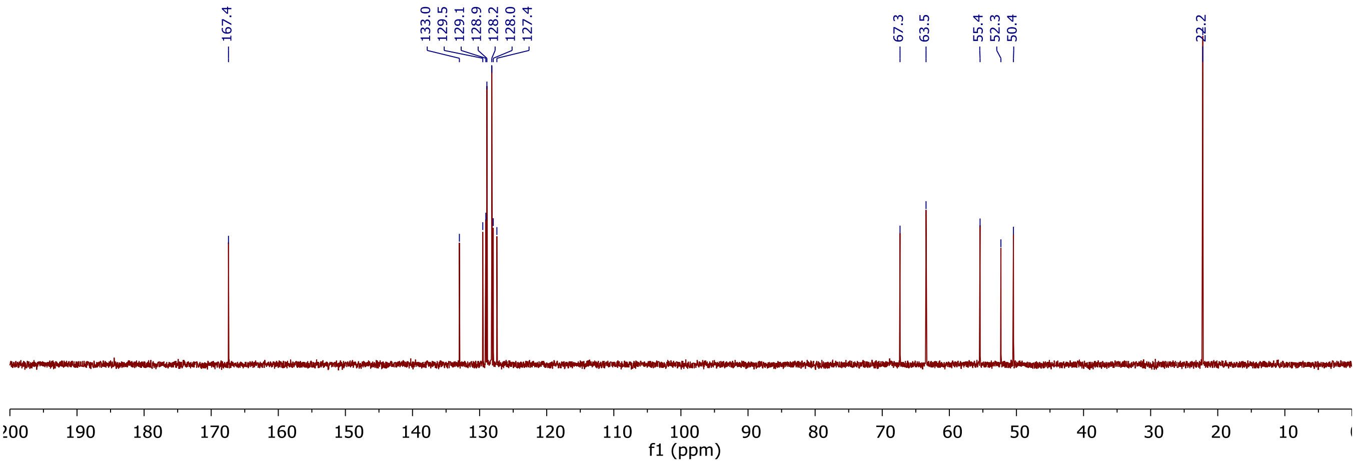

Methyl (2*S*,3*R*,4*S*,5*R*)-4-((*E*)-(((*S*)-*tert*-butylsulfinyl)imino)methyl)-5-(3-chlorophenyl)-3-phenylpyrrolidine-2-carboxylate. ^{1}**H NMR (400 MHz, CDCl$_3$) of 3ad**

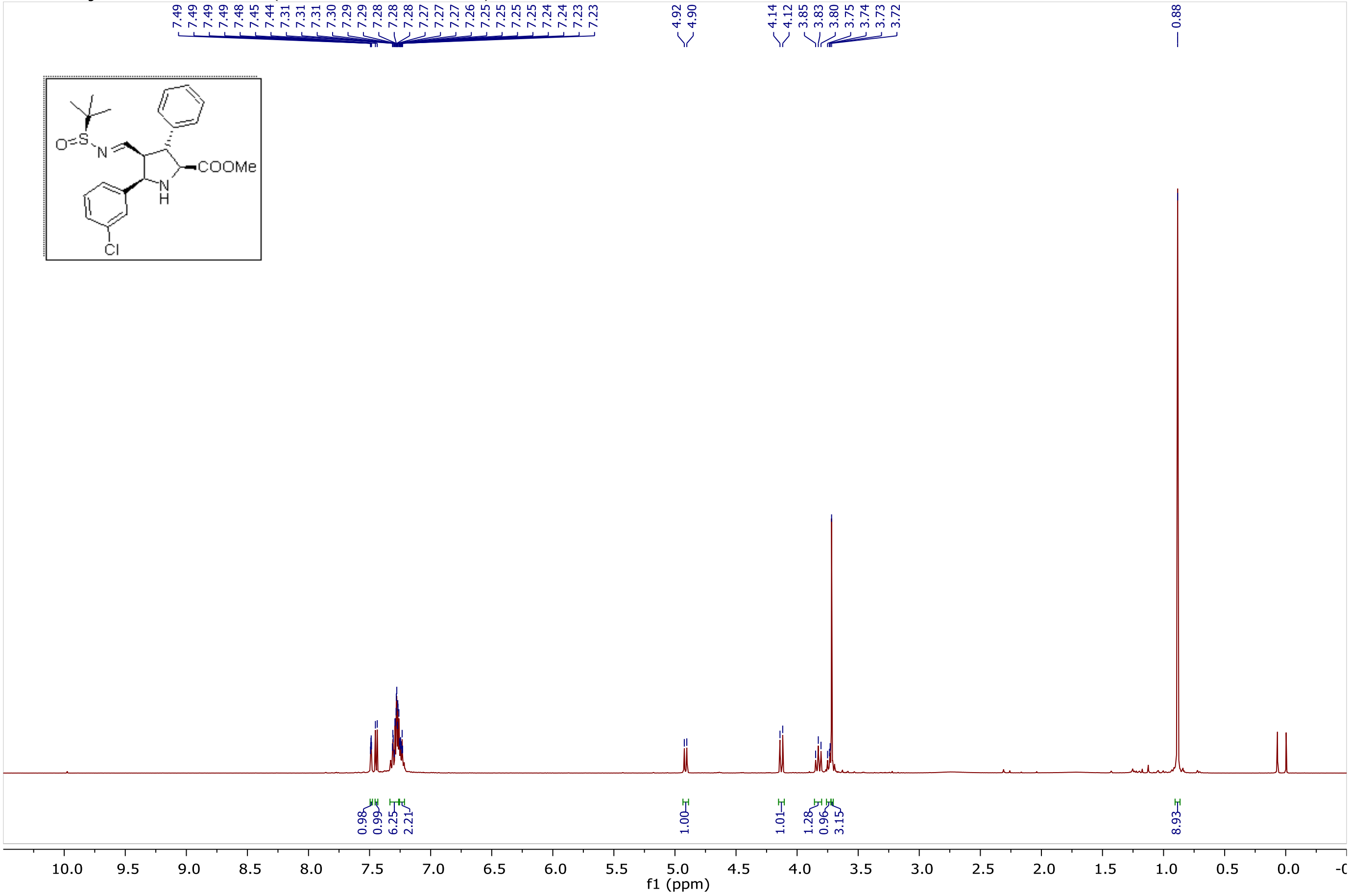

^{13}**C{H} NMR (101 MHz, CDCl$_3$) of 3ad**

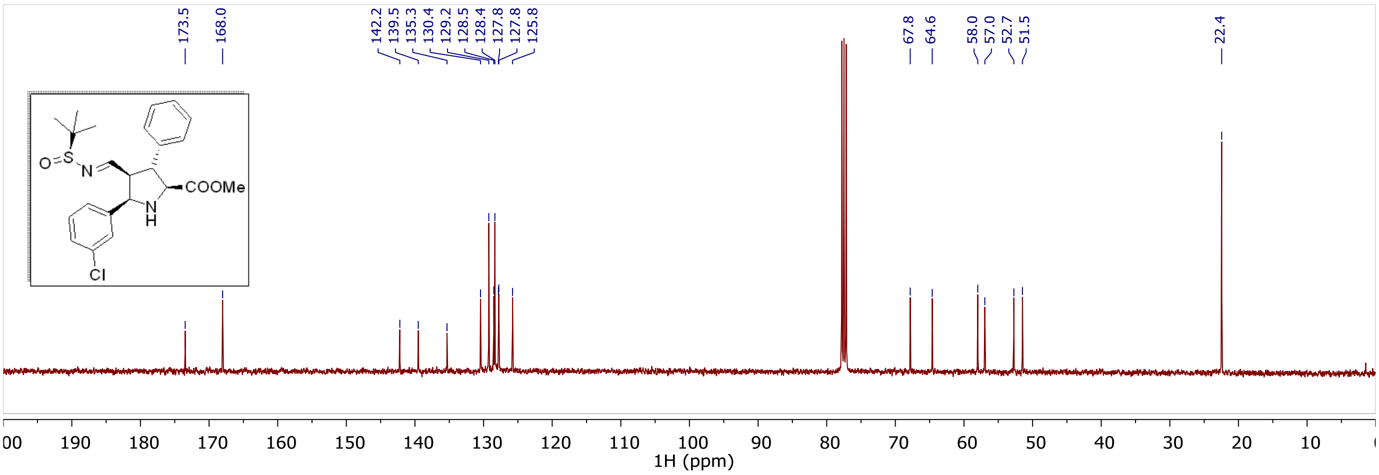

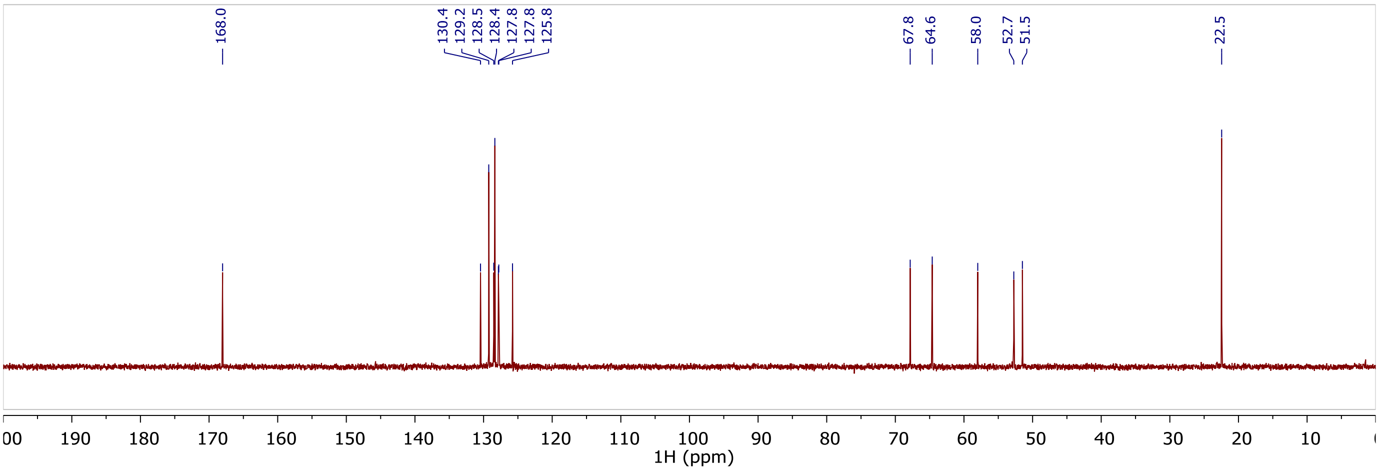

Methyl (2*S*,3*R*,4*S*,5*R*)-4-((*E*)-(((*S*)-*tert*-butylsulfinyl)imino)methyl)-3-phenyl-5-(p-tolyl)pyrrolidine-2-carboxylate. ^{1}H NMR (300 MHz, $CDCl_3$) of 3ae

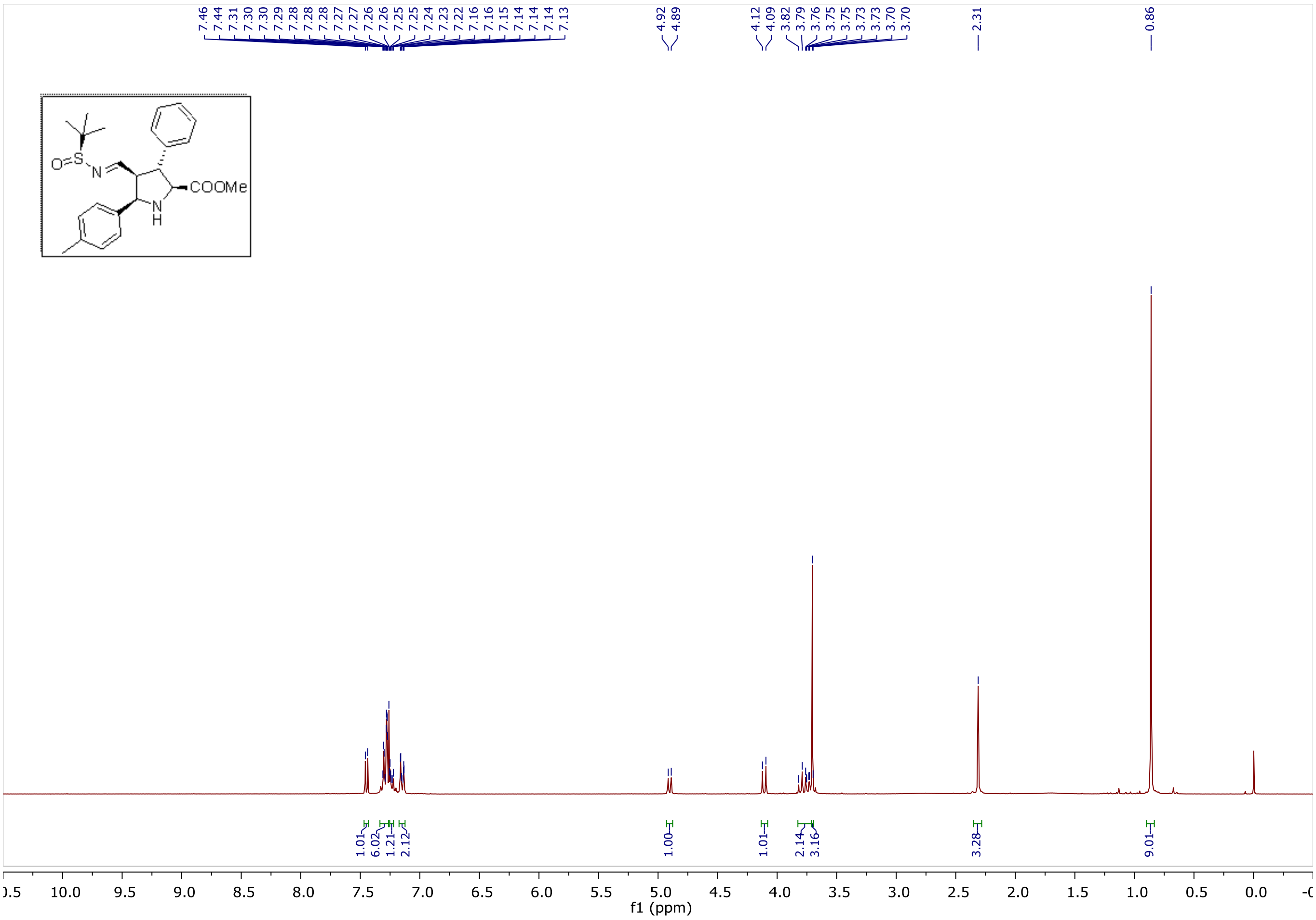

^{13}C{H} NMR (101 MHz, $CDCl_3$) of 3ae

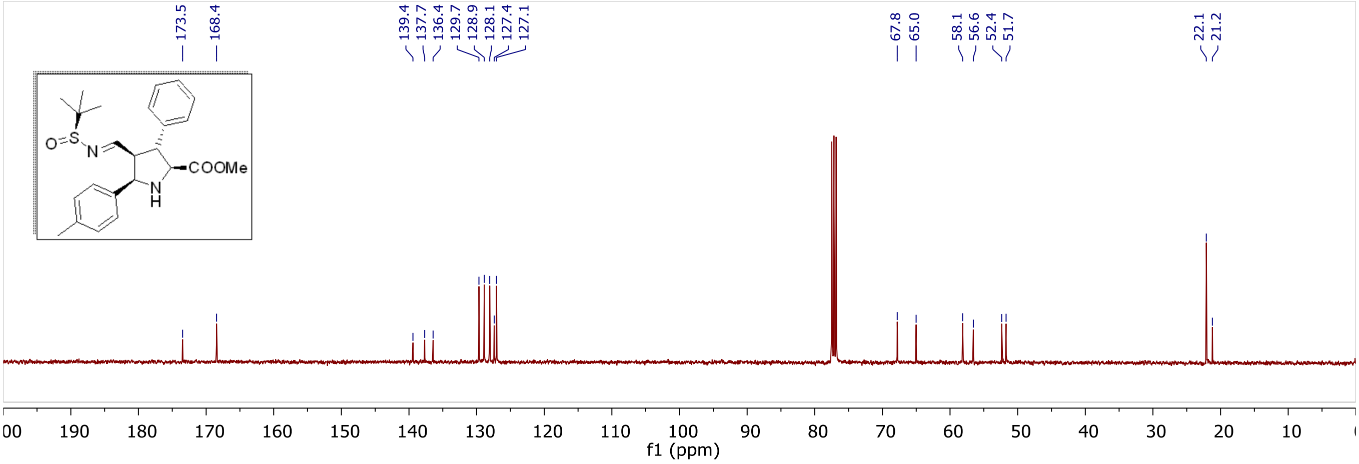

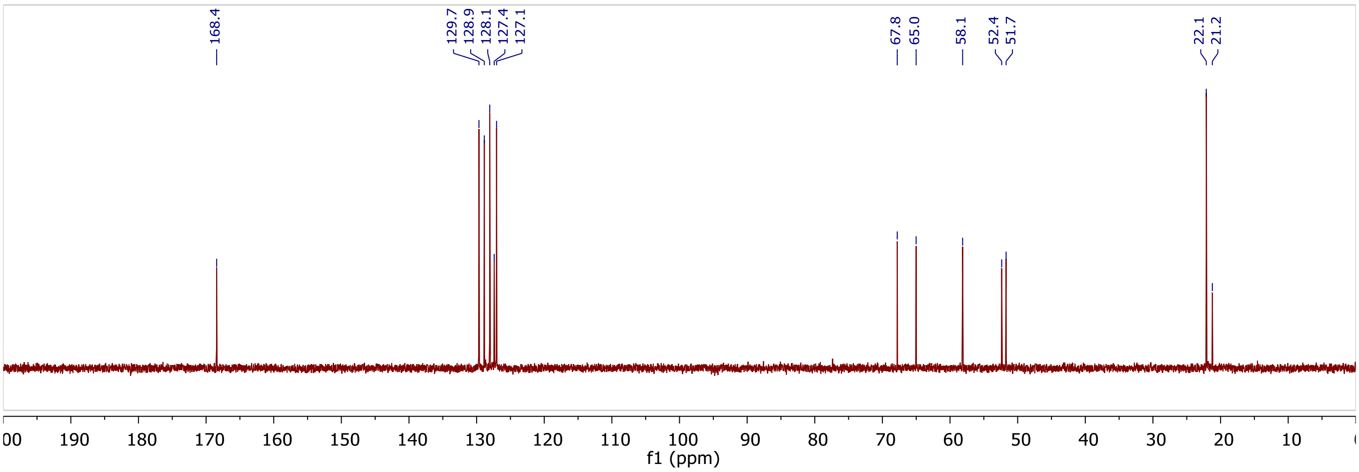

Methyl (2*S*,3*R*,4*S*,5*R*)-4-((*E*)-(((*S*)-*tert*-butylsulfinyl)imino)methyl)-3-phenyl-5-(o-tolyl)pyrrolidine-2-carboxylate. ^{1}H NMR (300 MHz, $CDCl_3$) of 3af

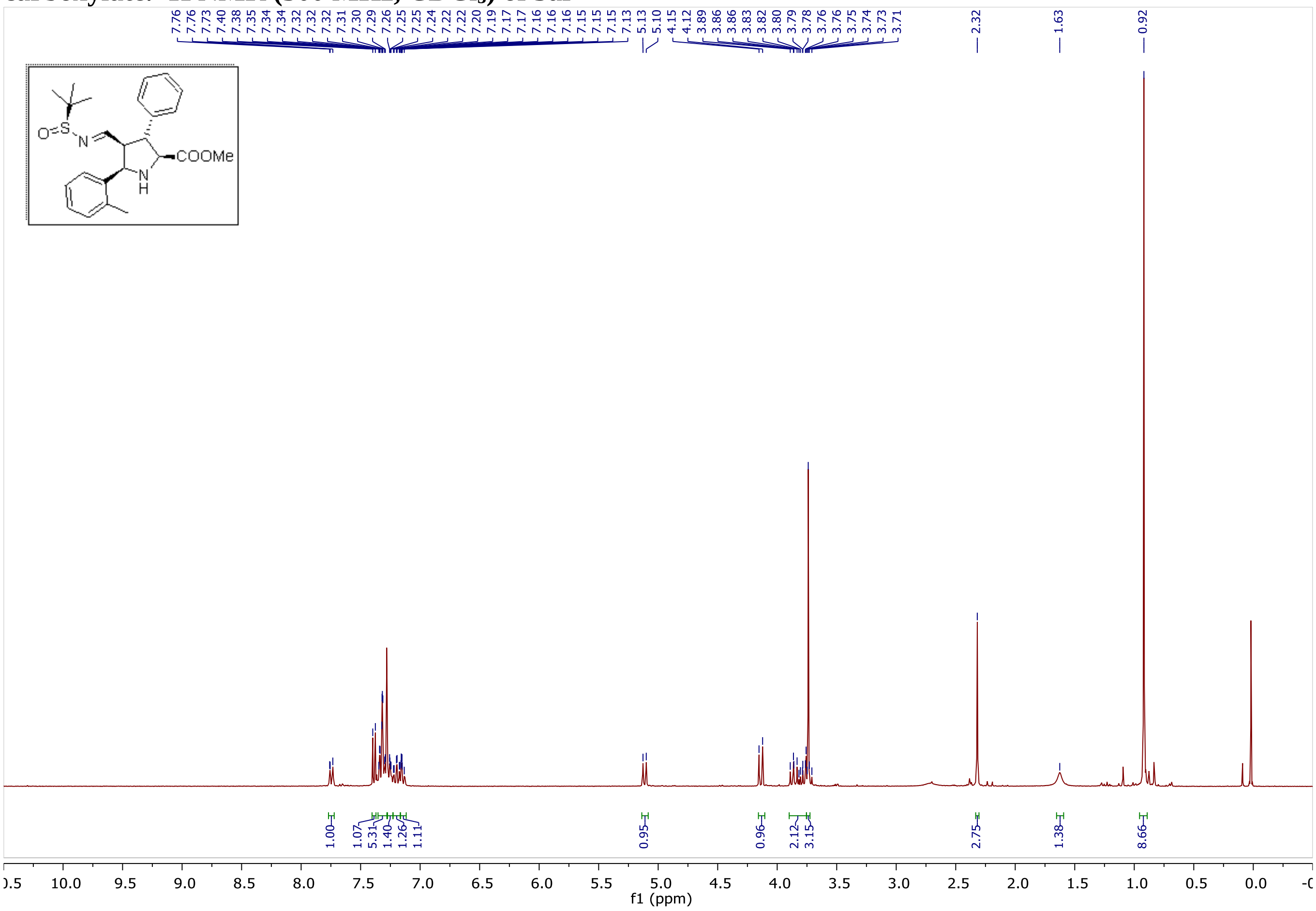

^{13}C{H} NMR (101 MHz, $CDCl_3$) of 3af

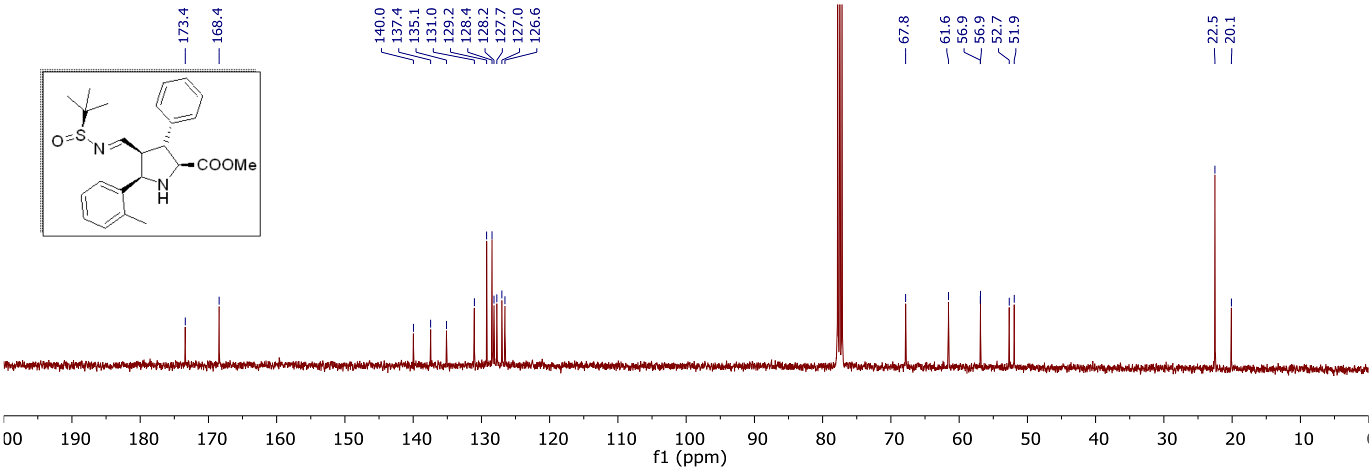

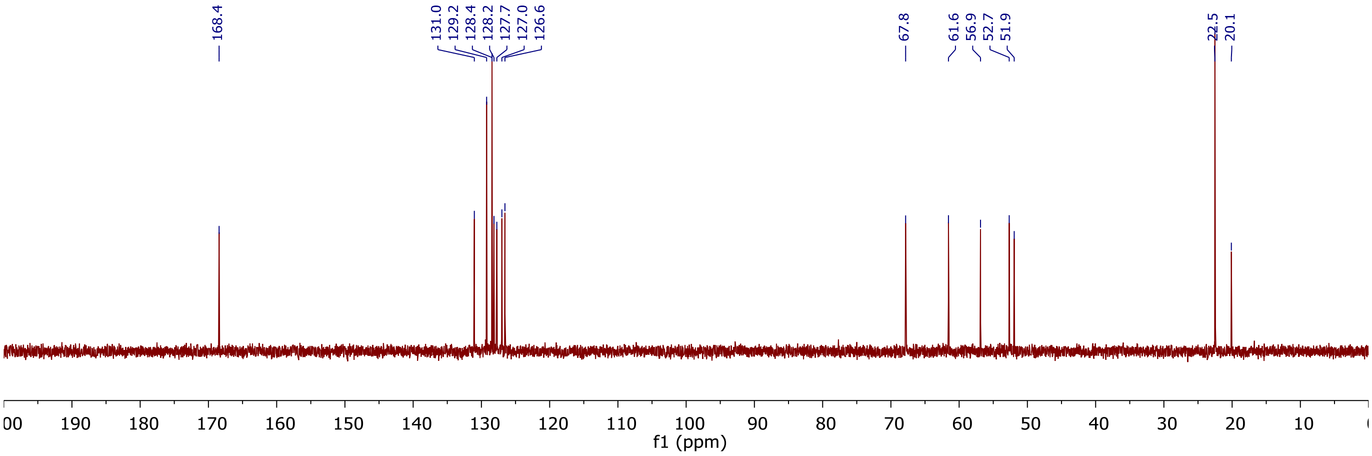

Methyl (2*S*,3*R*,4*S*,5*R*)-4-((*E*)-(((*S*)-*tert*-butylsulfinyl)imino)methyl)-5-(4-methoxyphenyl)-3-phenylpyrrolidine-2-carboxylate. **^{1}H NMR (400 MHz, $CDCl_3$) of 3ag**

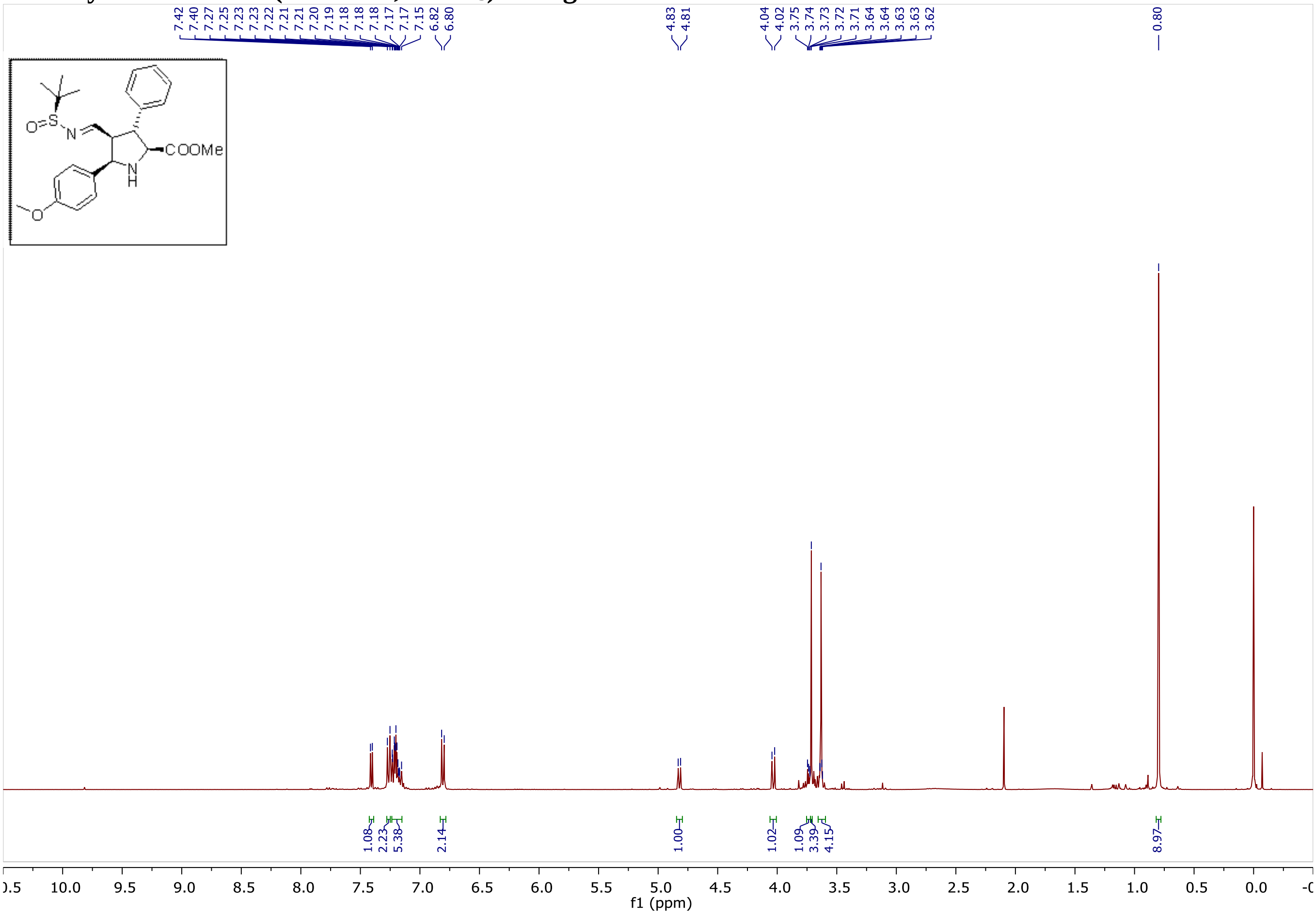

^{13}C{H} NMR (101 MHz, $CDCl_3$) of 3ag

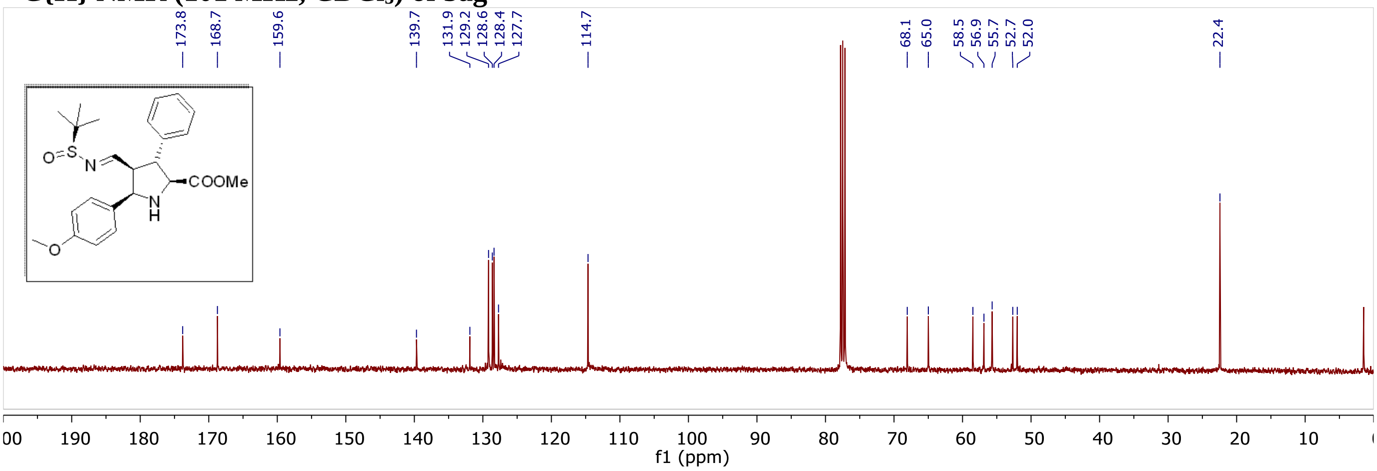

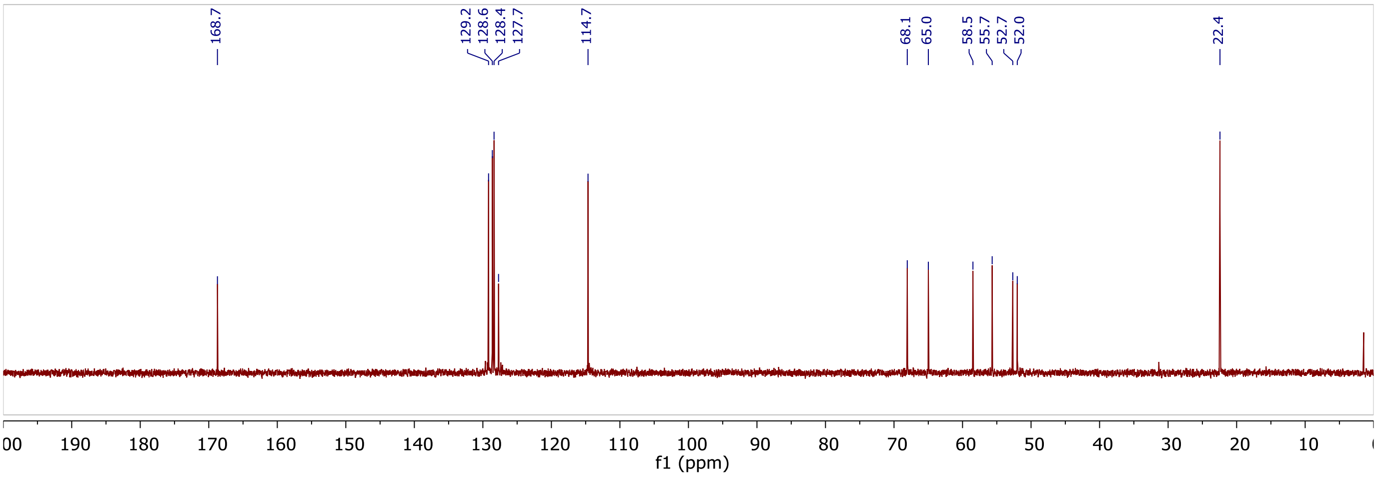

Methyl (2*S*,3*R*,4*S*,5*R*)-4-((*E*)-(((*S*)-*tert*-butylsulfinyl)imino)methyl)-3-phenyl-5-(thiophen-2-yl)pyrrolidine-2-carboxylate. **^{1}H NMR (300 MHz, $CDCl_3$) of 3ah**

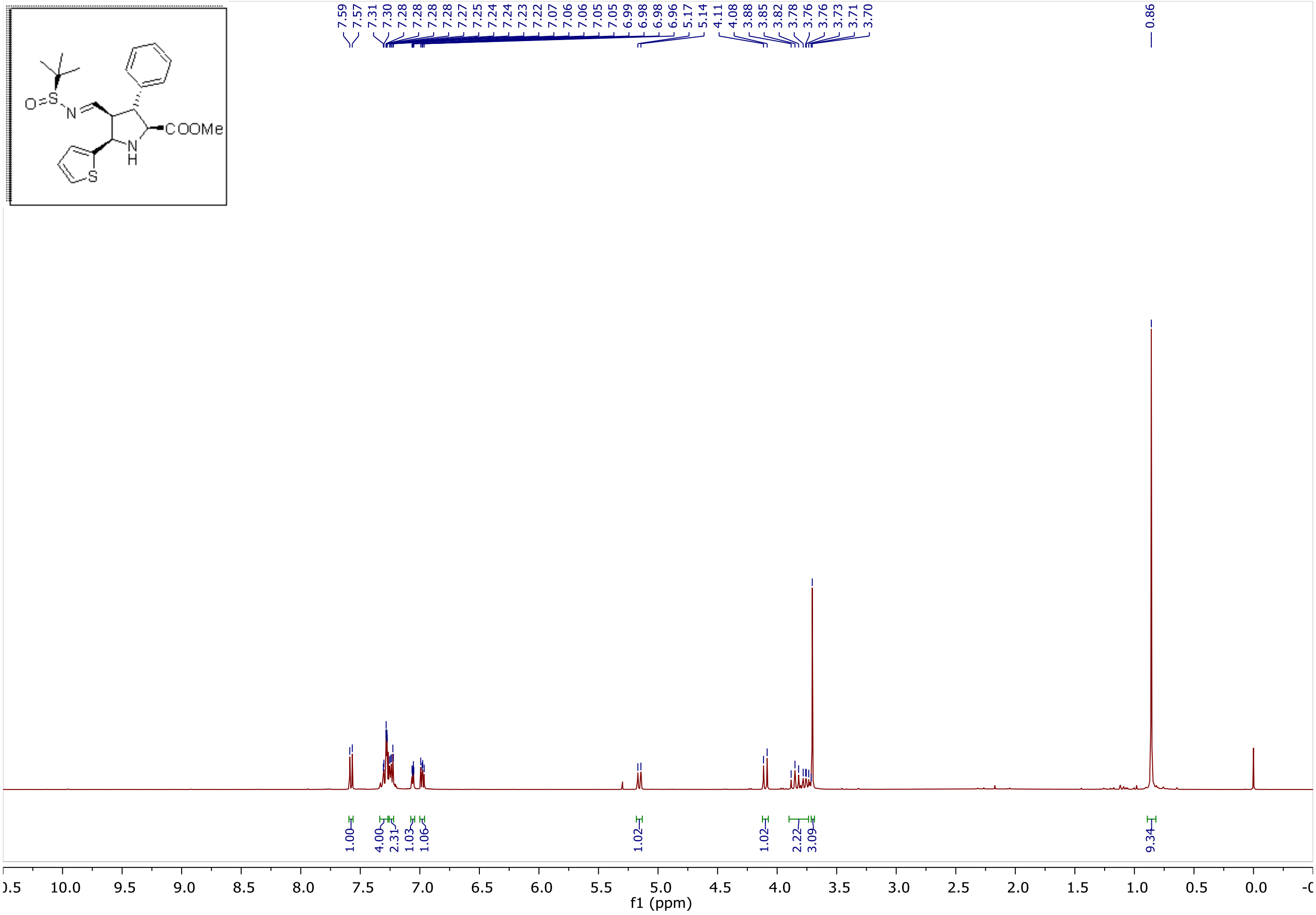

^{13}C{H} NMR (101 MHz, $CDCl_3$) of 3ah

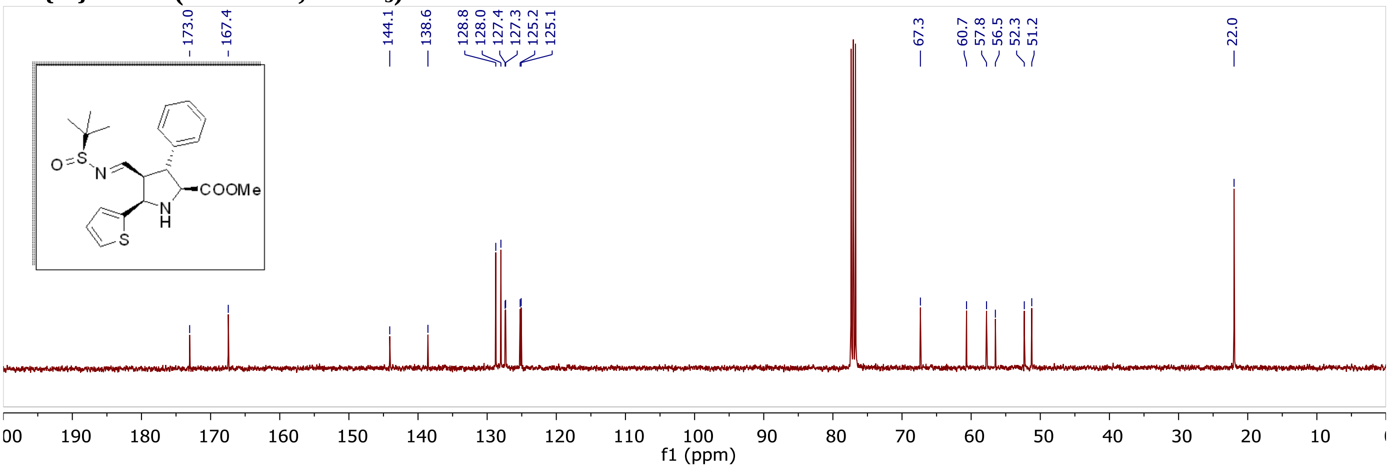

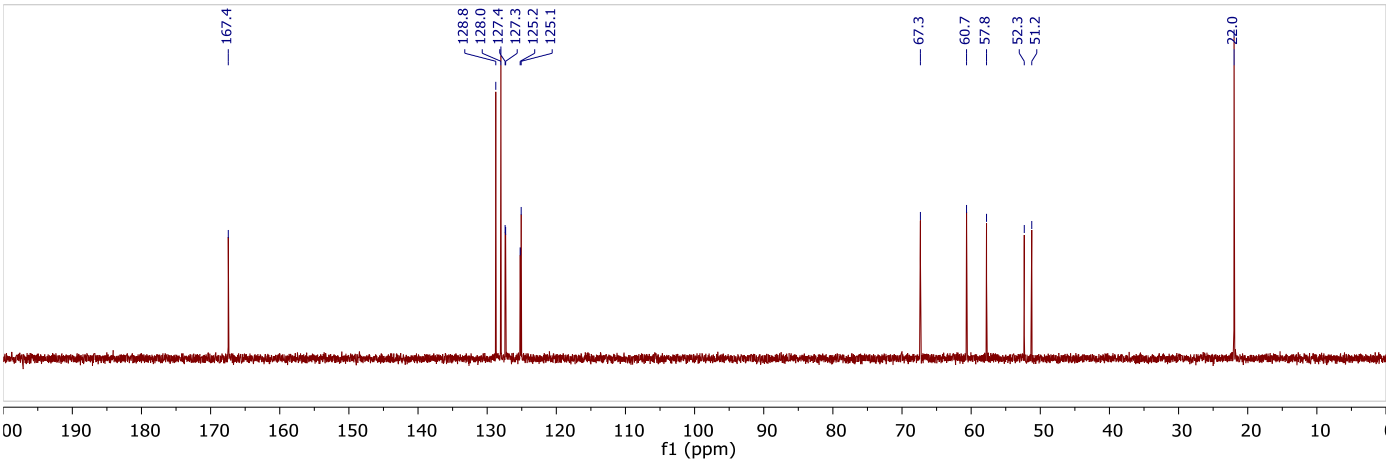

Tert-butyl (2_S_,3_R_,4_S_,5_R_)-5-(4-bromophenyl)-4-((_E_)-(((_S_)-_tert_-butylsulfinyl)imino)methyl)-3-phenylpyrrolidine-2-carboxylate ^{1}H NMR (300 MHz, $CDCl_3$) of 3ai

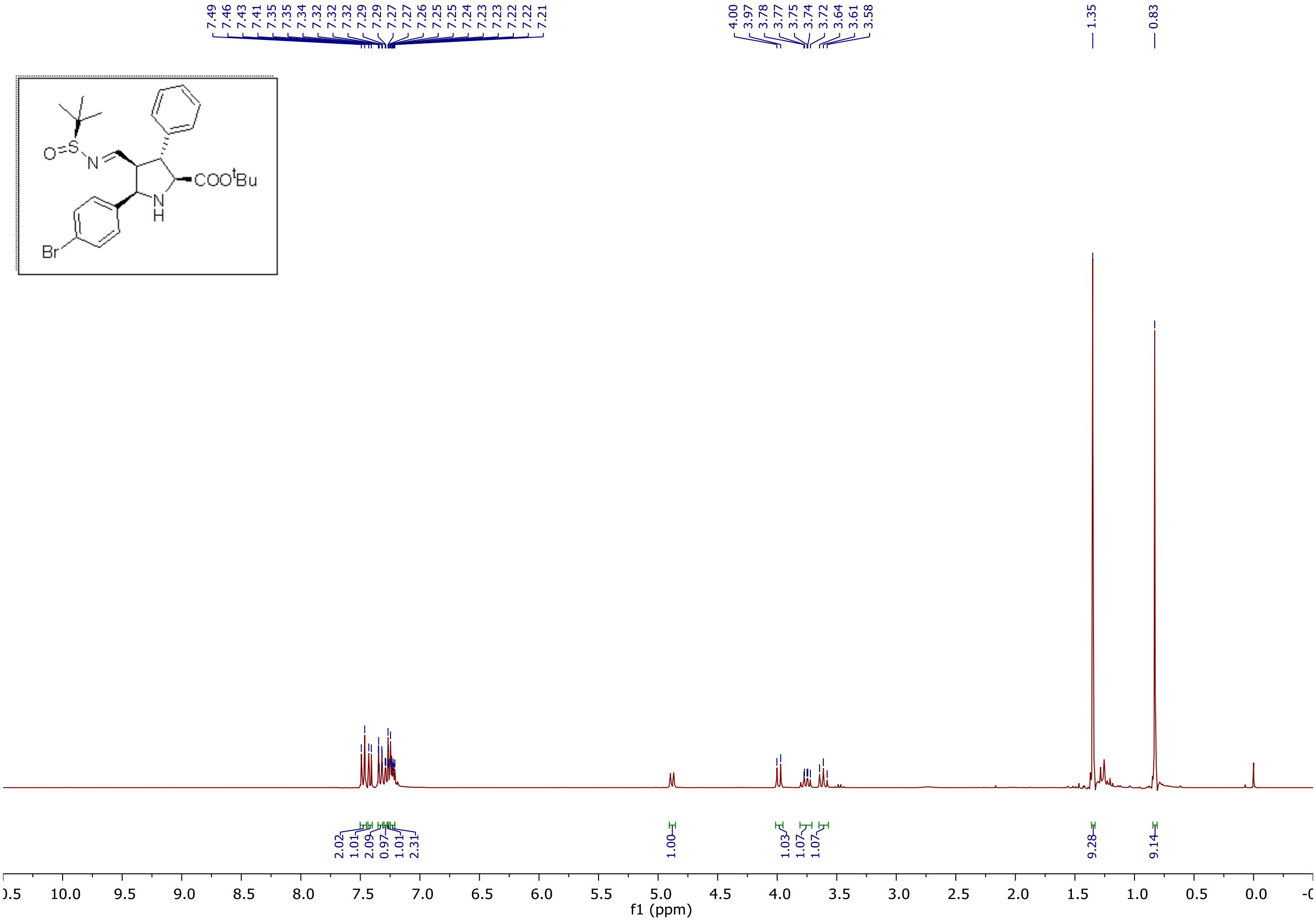

^{13}C{H} NMR (101 MHz, $CDCl_3$) of 3ai

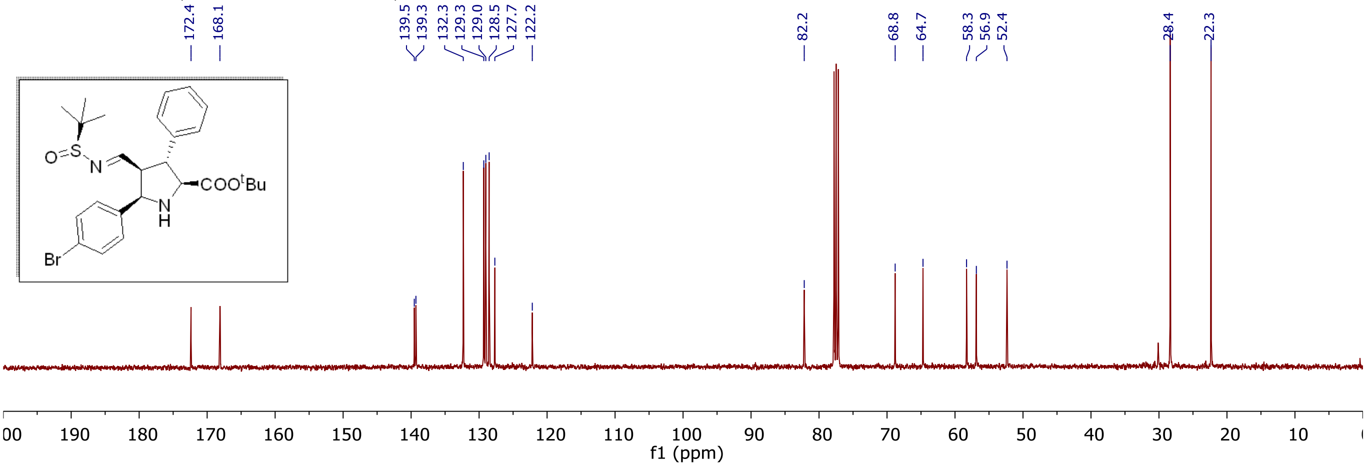

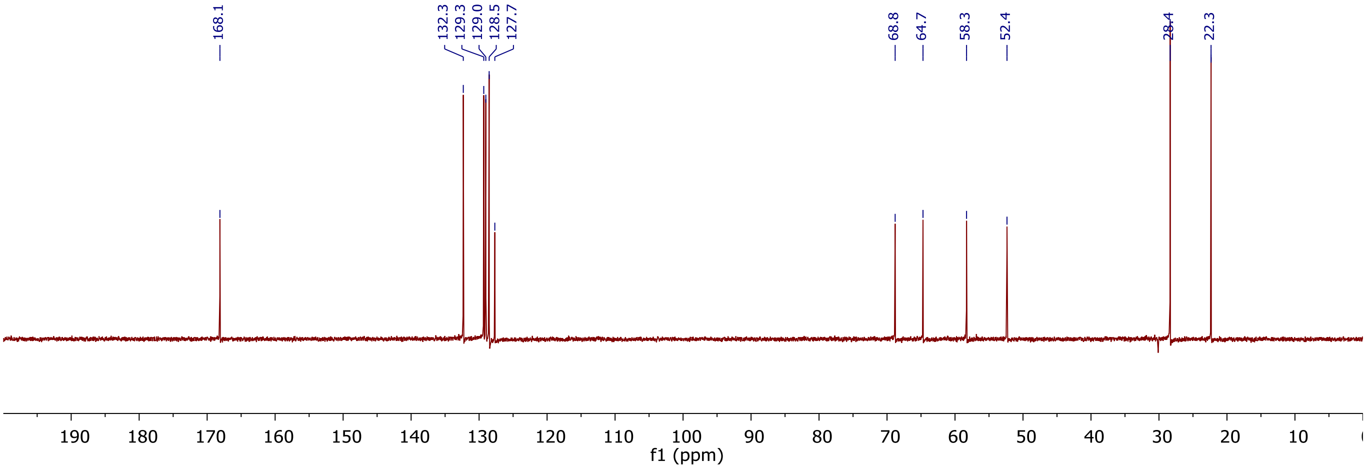

Tert-butyl (2_S_,3_R_,4_S_,5_R_)-5-(4-bromophenyl)-3-((_E_)-(((_S_)-_tert_-butylsulfinyl)imino)methyl)-4-phenylpyrrolidine-2-carboxylate ^{1}H NMR (300 MHz, $CDCl_3$) of 3ai'

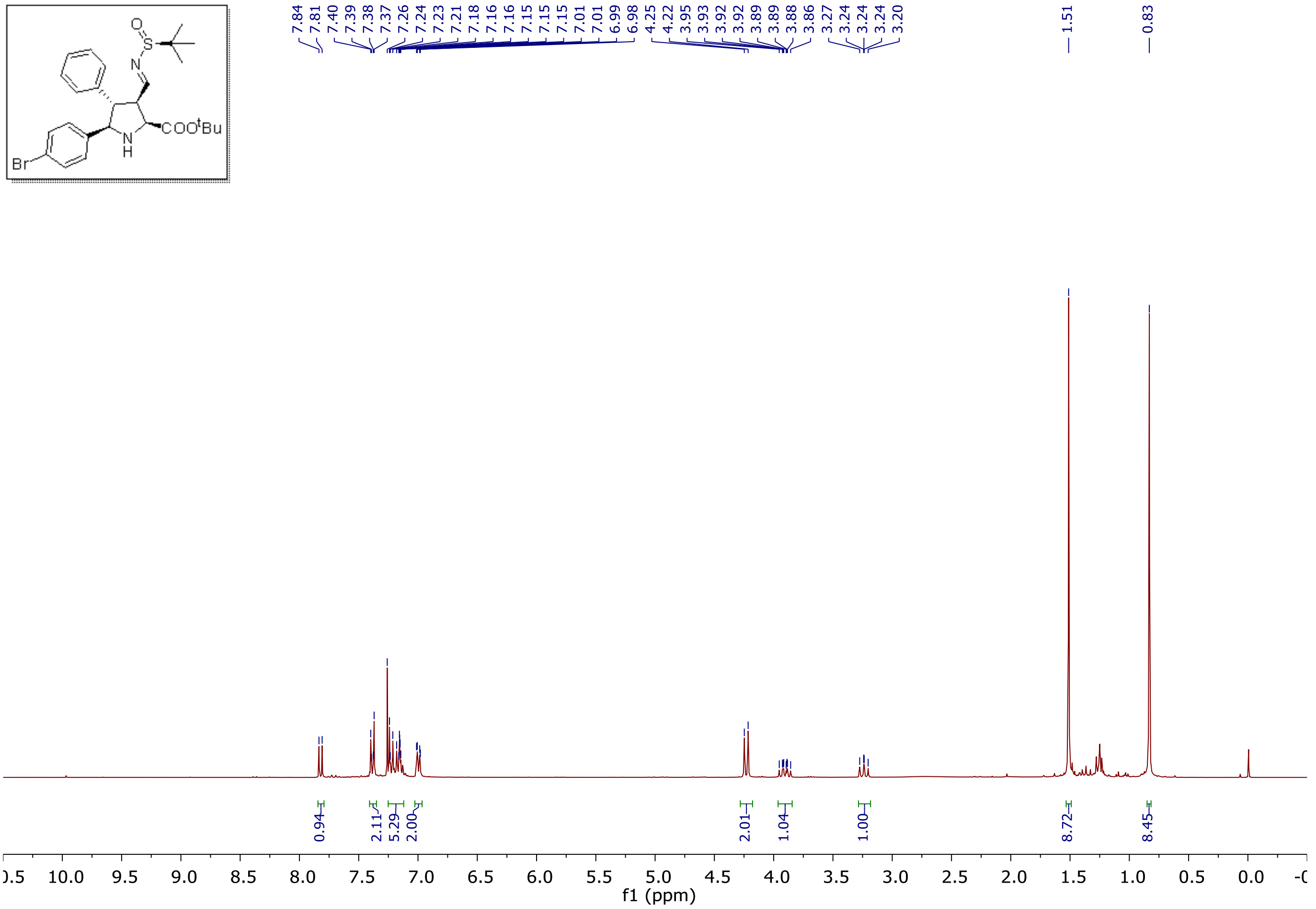

^{13}C{H} NMR (101 MHz, $CDCl_3$) of 3ai'

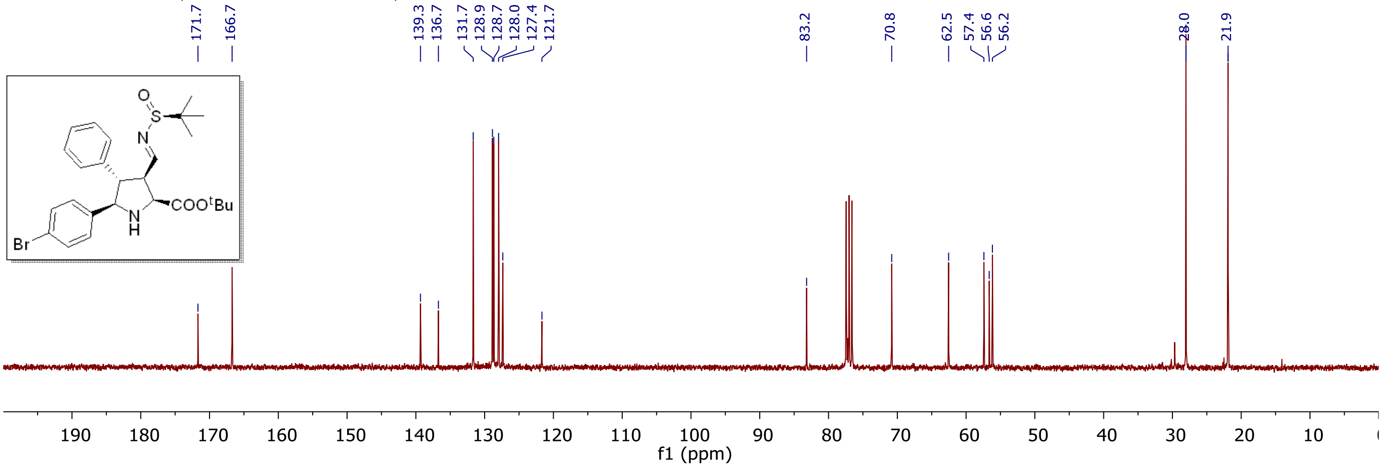

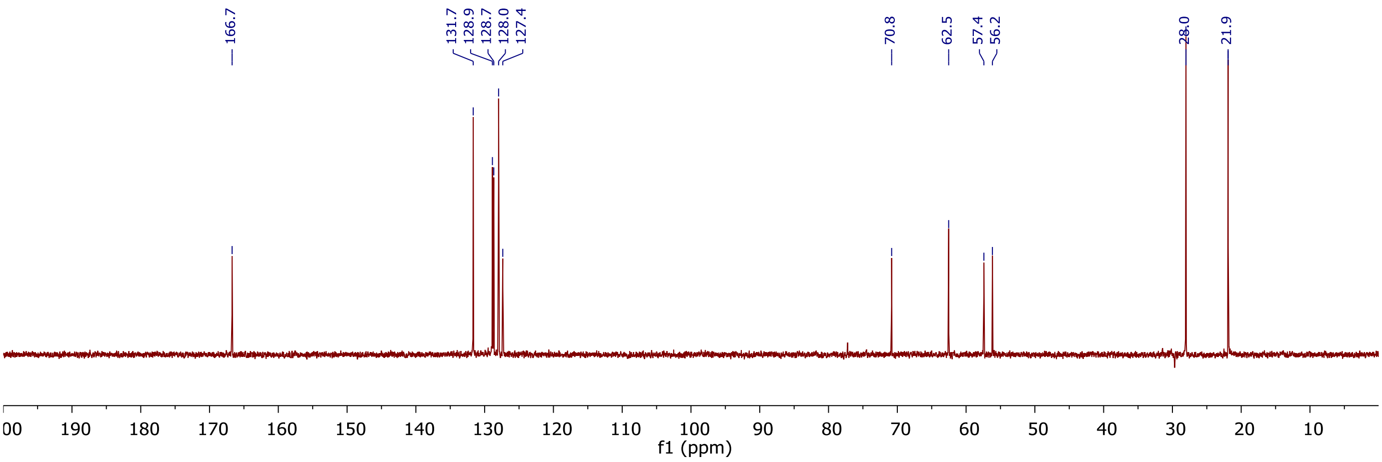

Benzyl (2*S*,3*R*,4*S*,5*R*)-5-(4-bromophenyl)-4-((*E*)-(((*S*)-*tert*-butylsulfinyl)imino)methyl)-3-phenylpyrrolidine-2-carboxylate ^{1}H NMR (300 MHz, $CDCl_3$) of 3aj

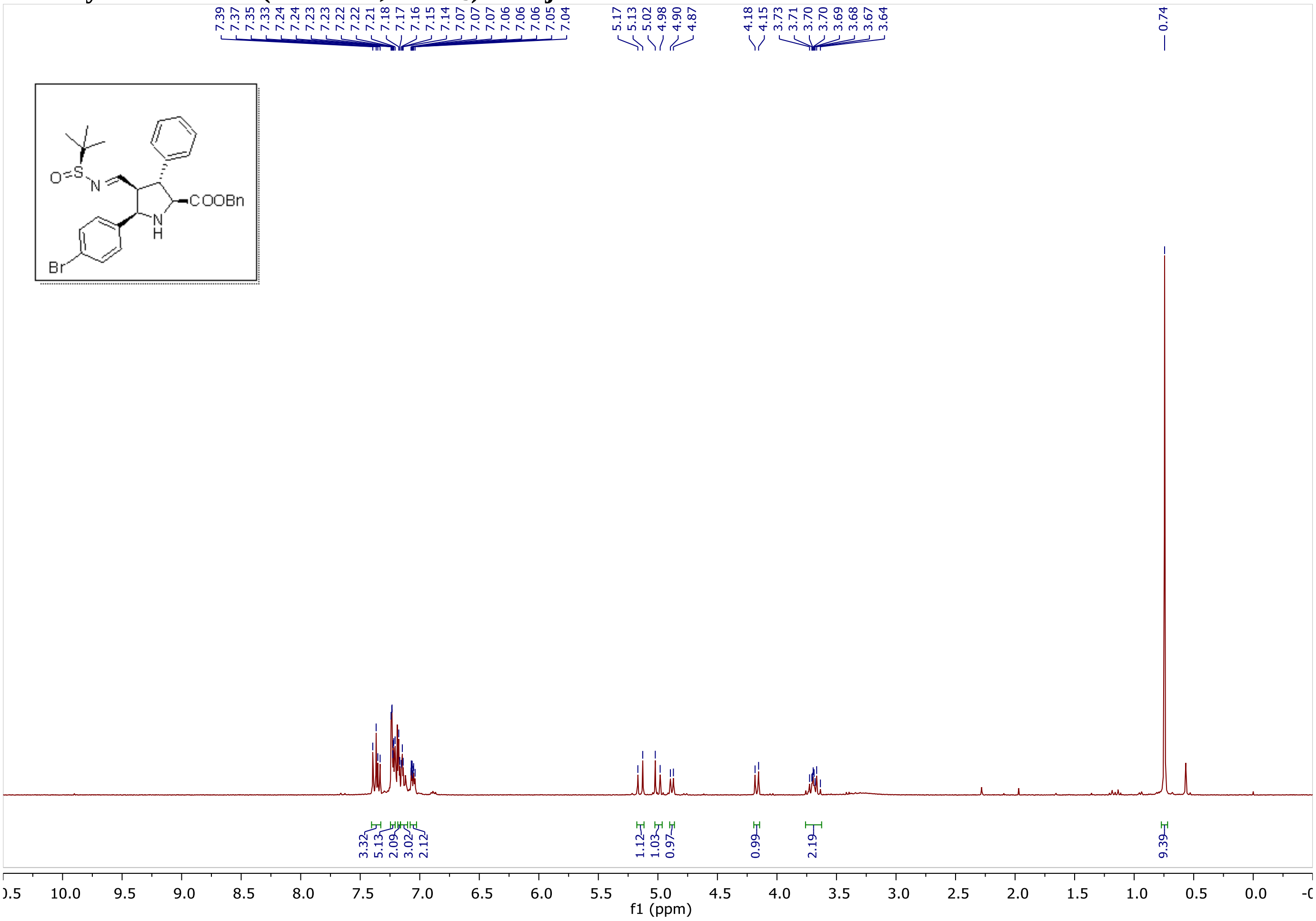

^{13}C{H} NMR (101 MHz, $CDCl_3$) of 3aj

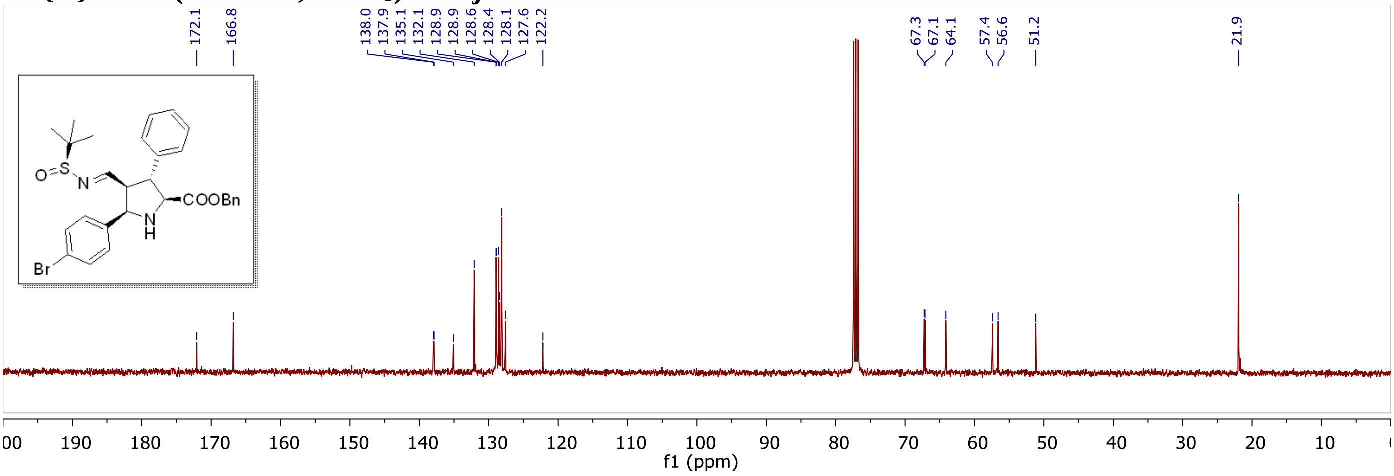

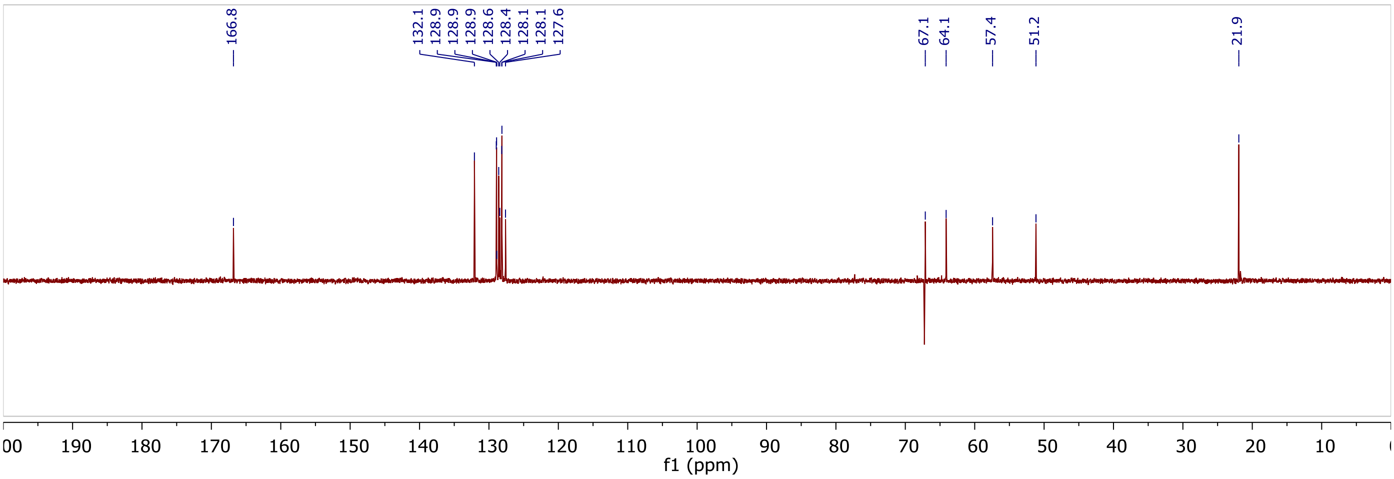

Methyl (2*S*,3*R*,4*S*,5*R*)-5-(4-bromophenyl)-4-((*E*)-(((*S*)-*tert*-butylsulfinyl)imino)methyl)-2-methyl-3-phenylpyrrolidine-2-carboxylate. ^{1}H NMR (300 MHz, $CDCl_3$) of 3ak

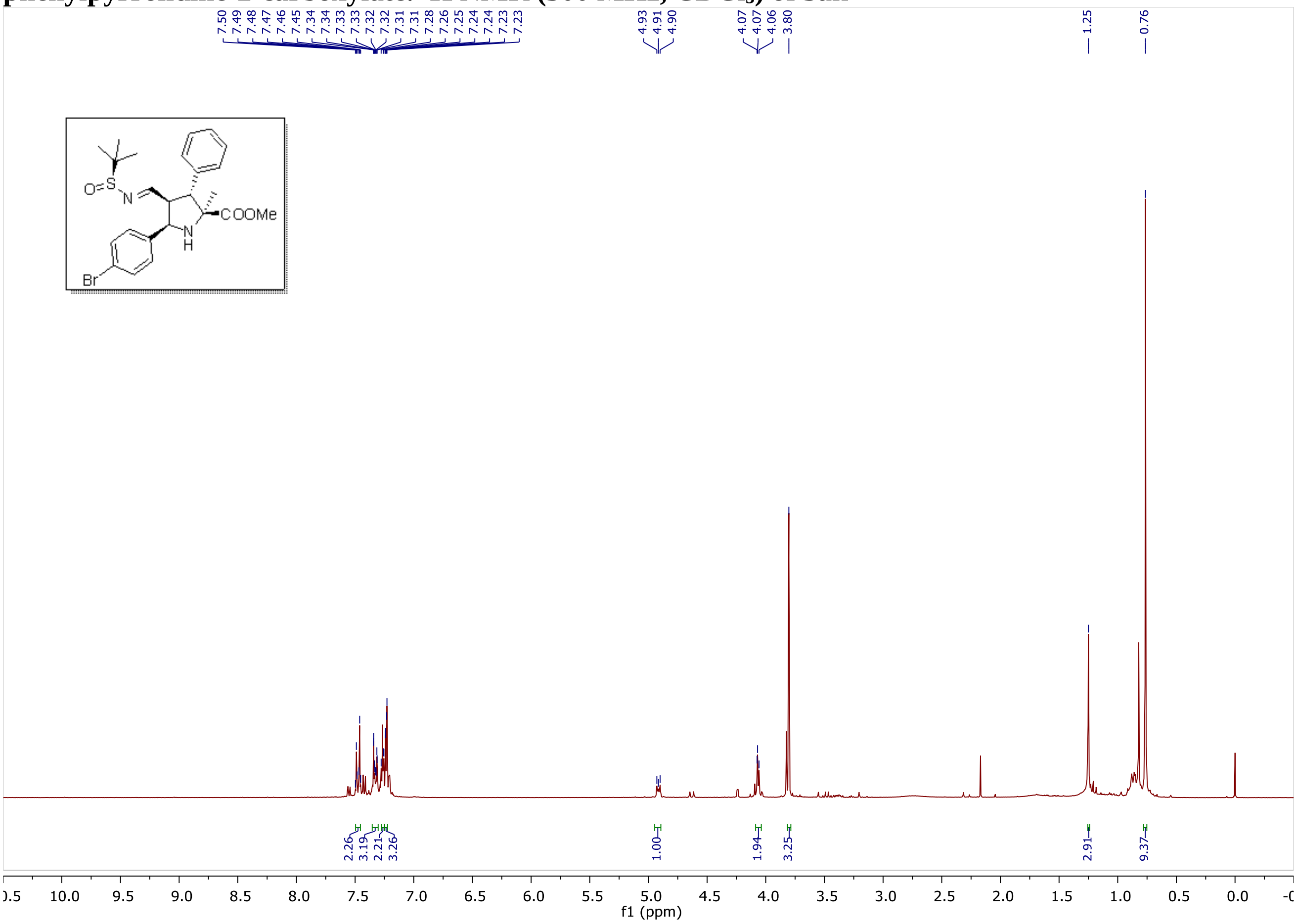

^{13}C{H} NMR (101 MHz, $CDCl_3$) of 3ak

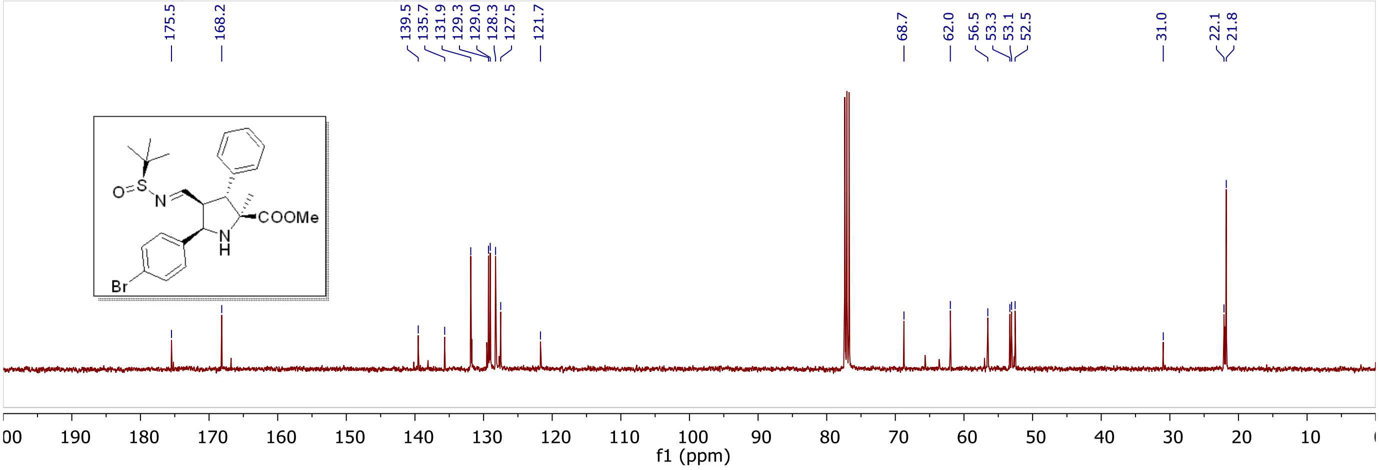

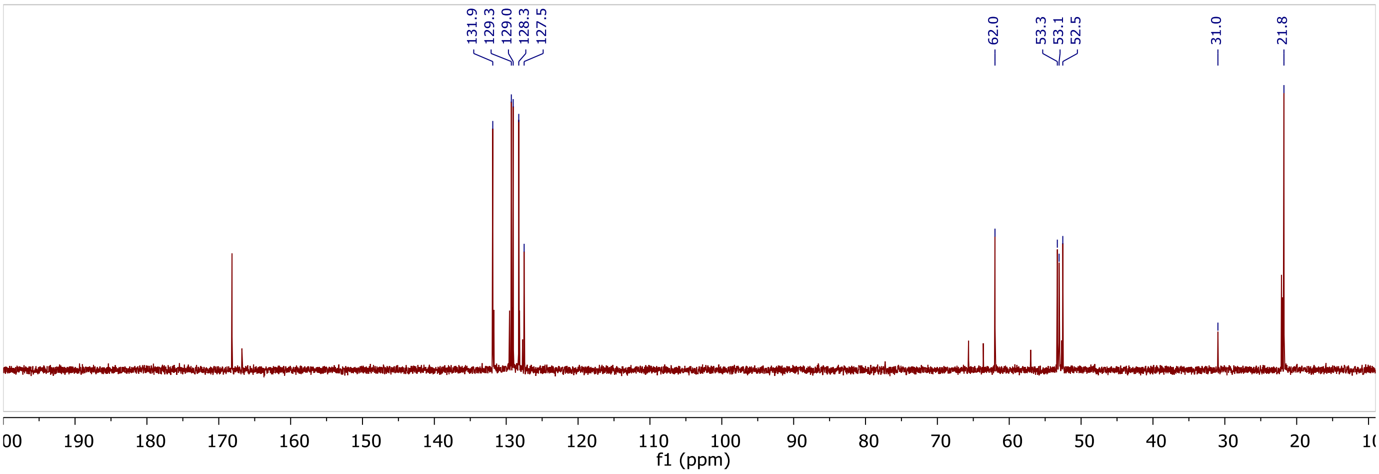

Methyl (2*S*,3*R*,4*S*,5*R*)-5-(4-bromophenyl)-4-((*E*)-(((*S*)-*tert*-butylsulfinyl)imino)methyl)-3-(4-chlorophenyl)pyrrolidine-2-carboxylate.^{1}H NMR (300 MHz, $CDCl_3$) of 3ba

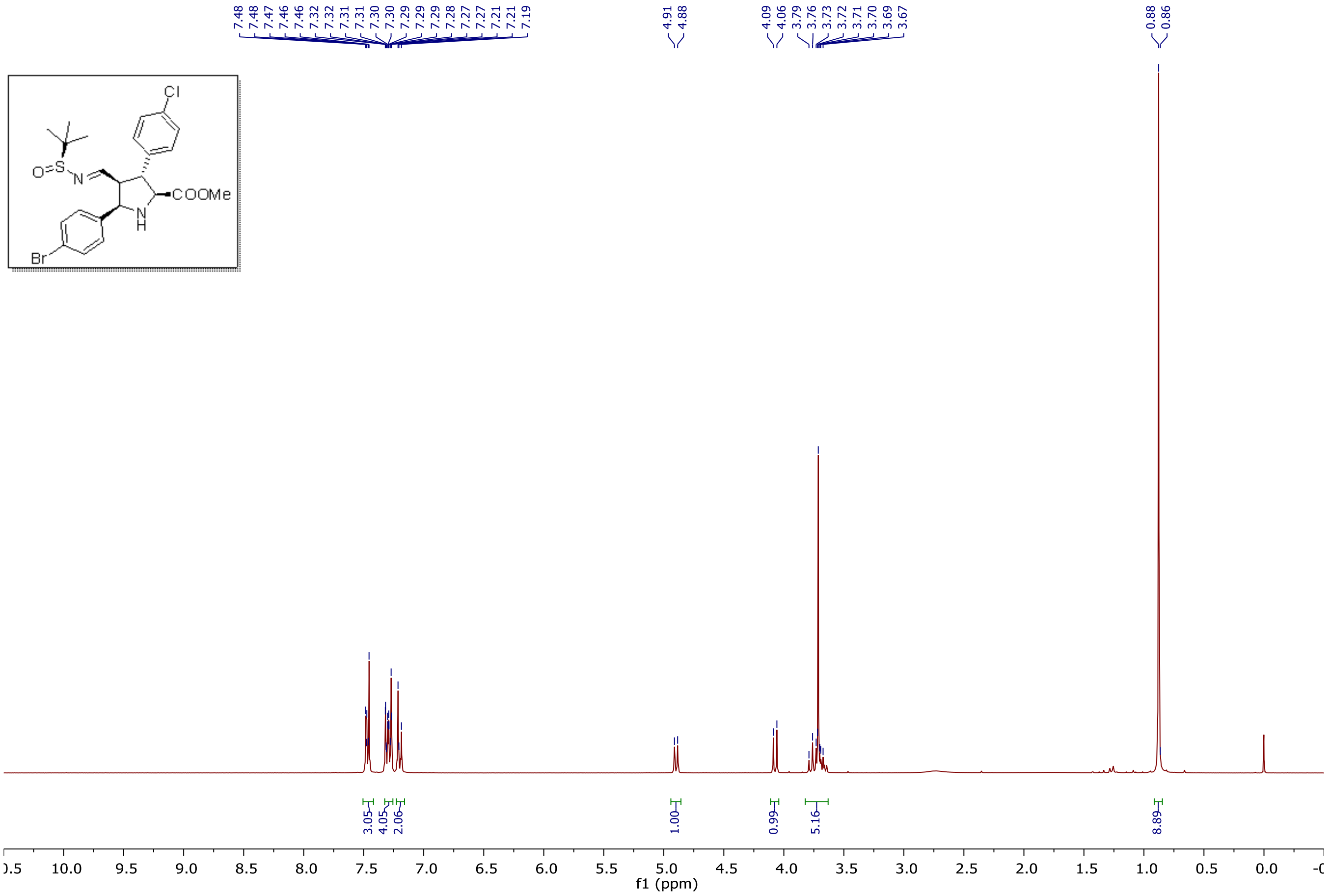

^{13}C{H} NMR (101 MHz, $CDCl_3$) of 3ba

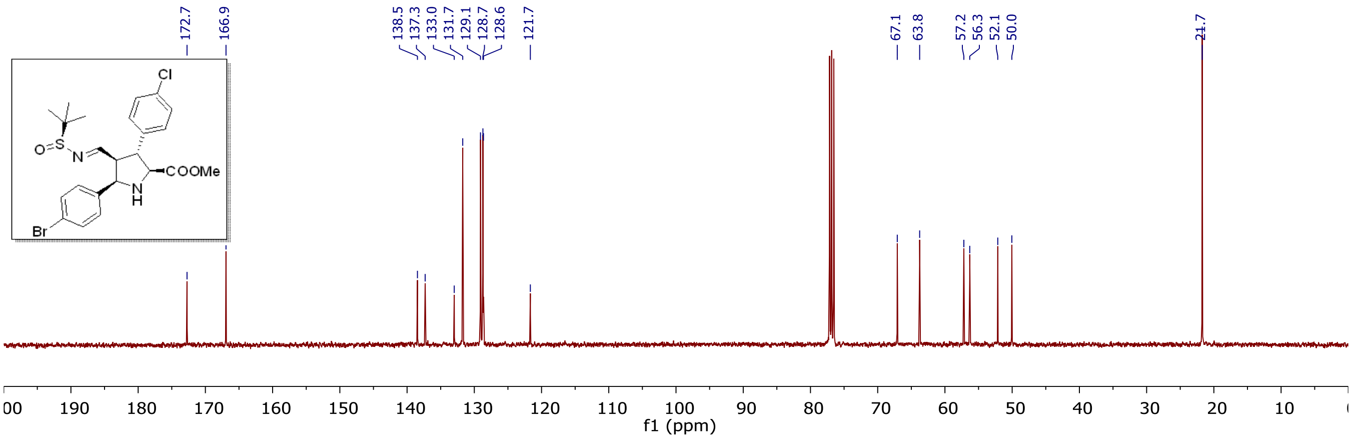

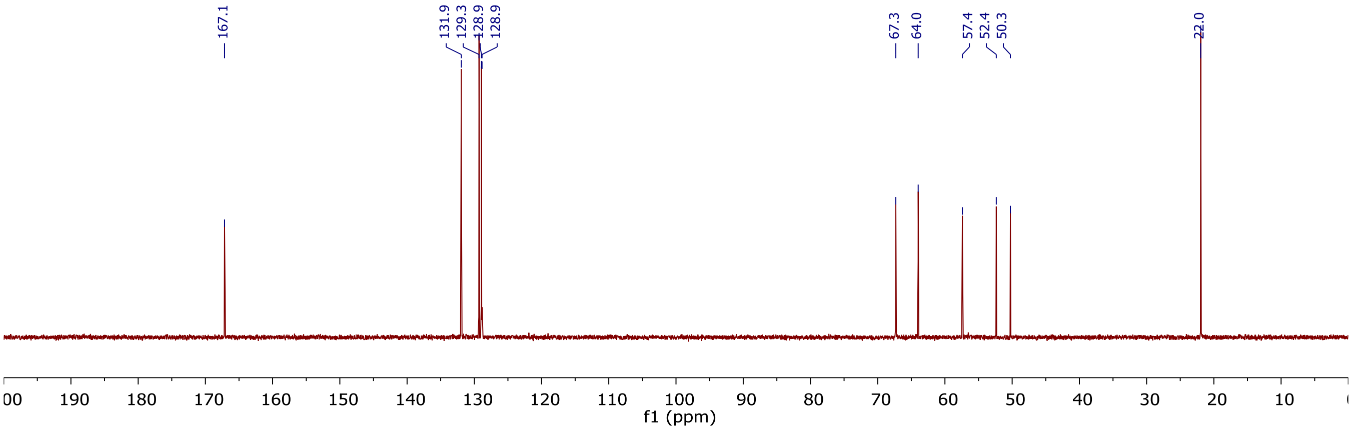

Methyl (2*S*,3*R*,4*S*,5*R*)-5-(4-bromophenyl)-4-((*E*)-(((*S*)-*tert*-butylsulfinyl)imino)methyl)-3-(p-tolyl)pyrrolidine-2-carboxylate ^{1}H NMR (300 MHz, CDCl$_3$) of 3ca

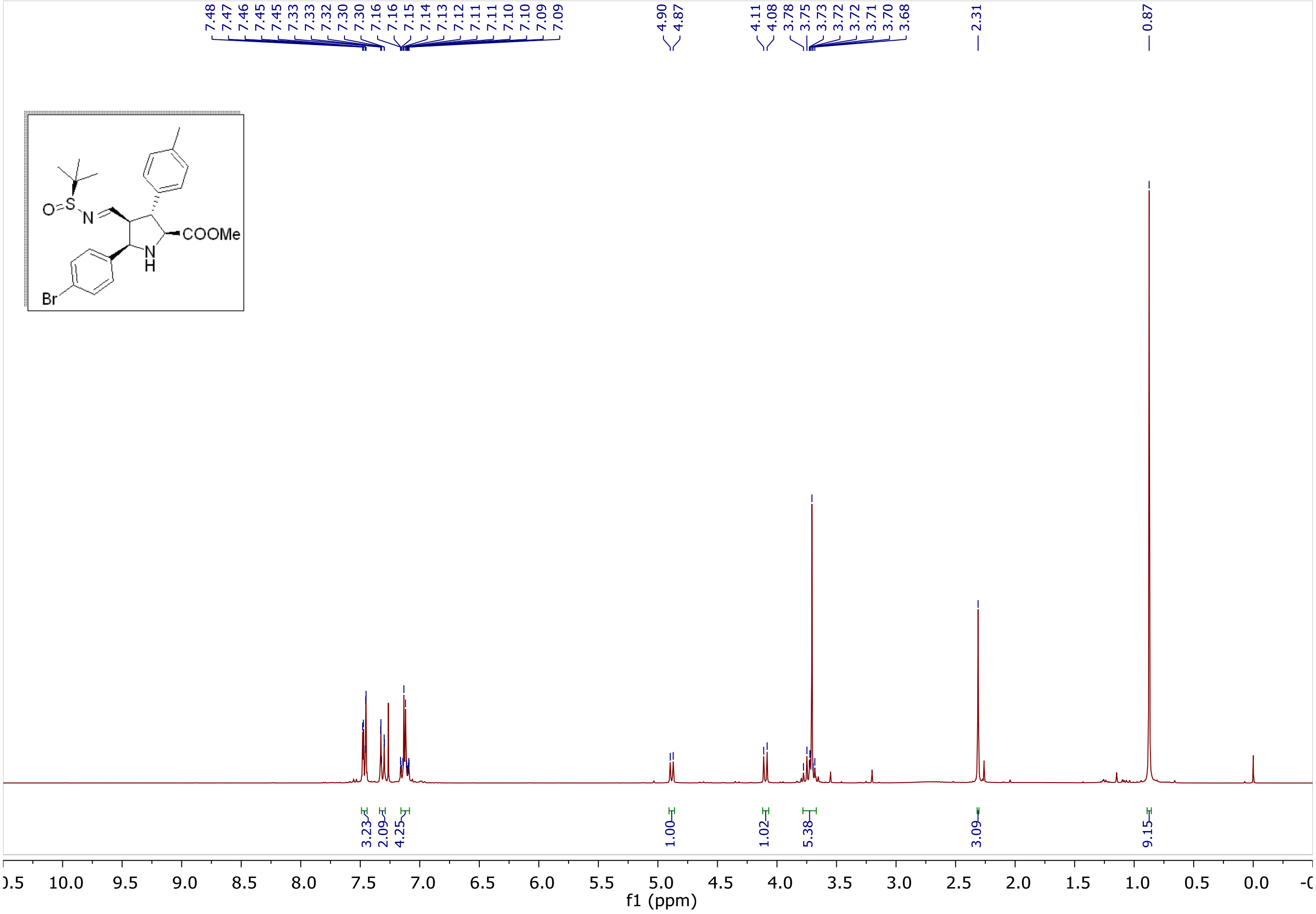

^{13}C{H} NMR (101 MHz, CDCl$_3$) of 3ca

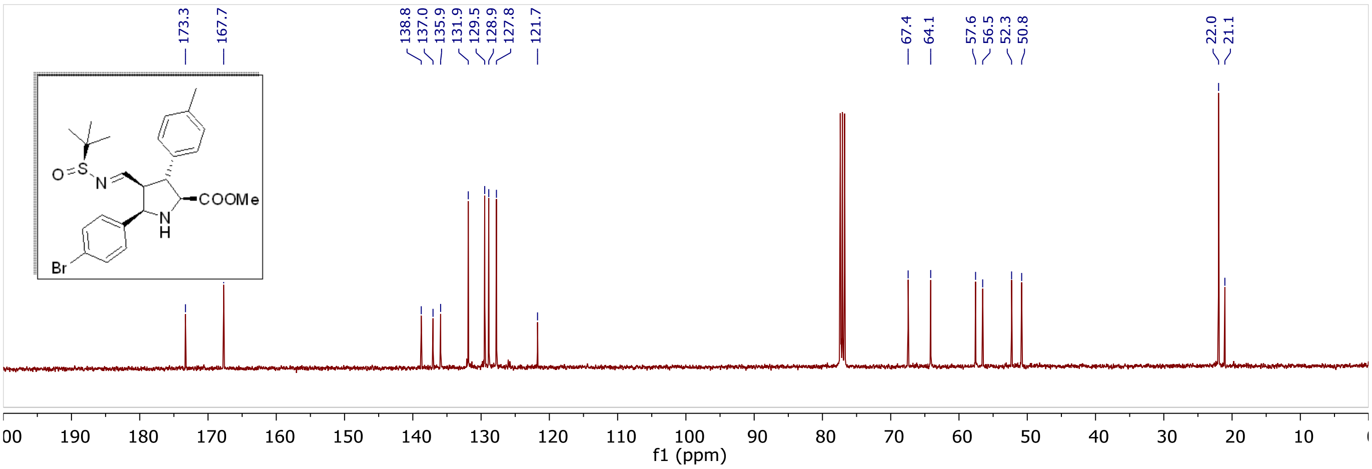

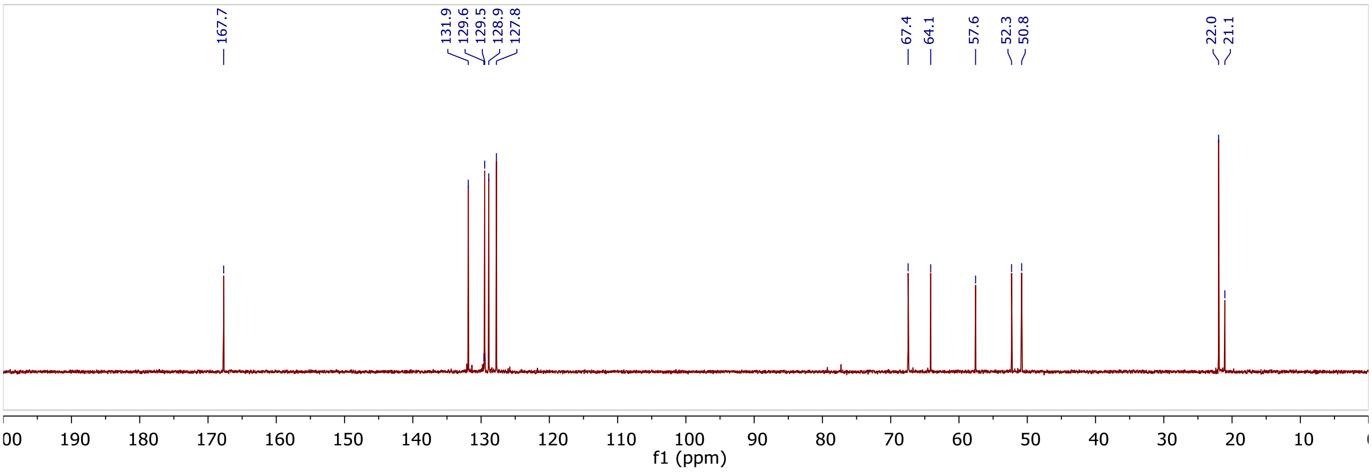

Methyl (2*S*,3*R*,4*S*,5*R*)-5-(4-bromophenyl)-4-((*E*)-(((*S*)-*tert*-butylsulfinyl)imino)methyl)-3-(4-methoxyphenyl)pyrrolidine-2-carboxylate ^{1}H NMR (400 MHz, $CDCl_3$) of 3da

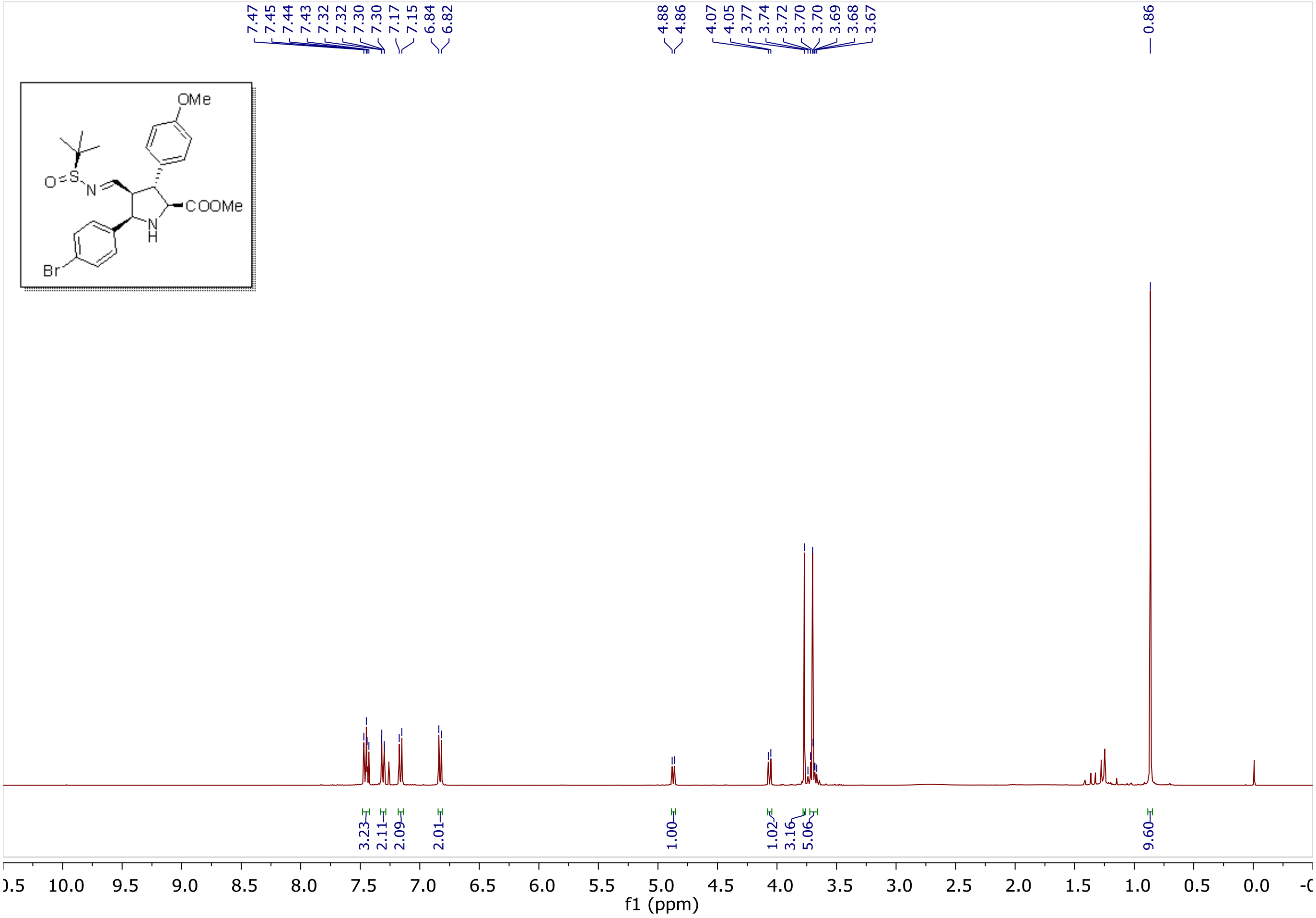

^{13}C{H} NMR (101 MHz, $CDCl_3$) of 3da

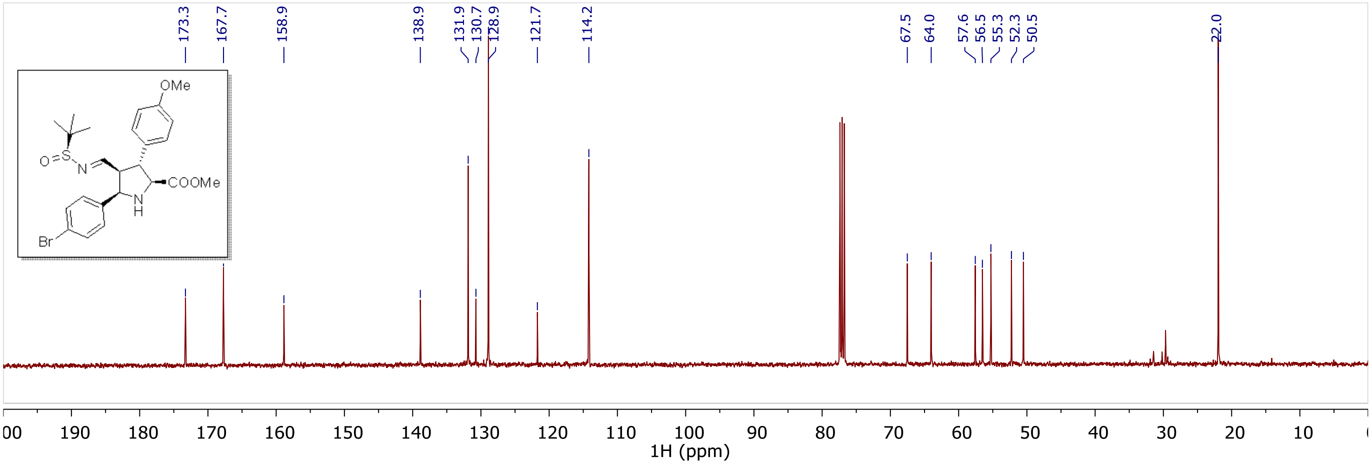

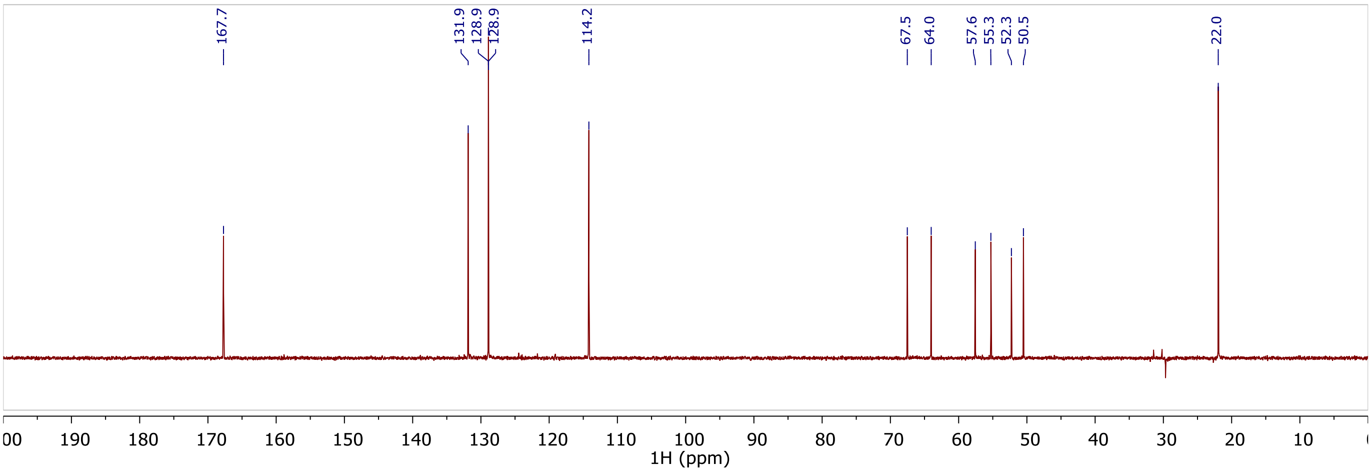

Methyl (2*S*,3*R*,4*S*,5*R*)-5-(2-bromophenyl)-4-((*E*)-(((*S*)-*tert*-butylsulfinyl)imino)methyl)-3-(4-methoxyphenyl)pyrrolidine-2-carboxylate ^{1}H NMR (300 MHz, $CDCl_3$) of 3dc

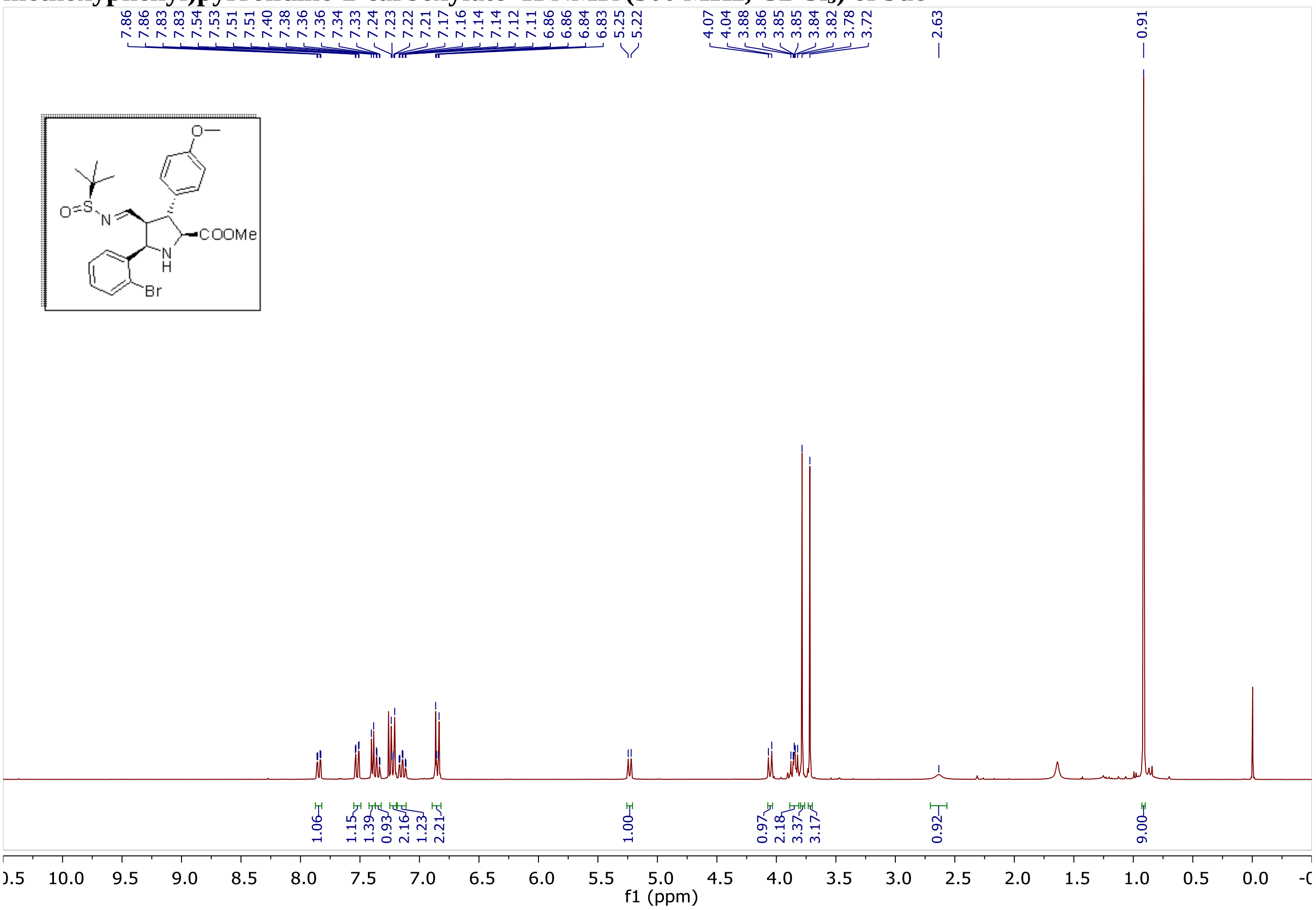

^{13}C{H} NMR (101 MHz, $CDCl_3$) of 3dc

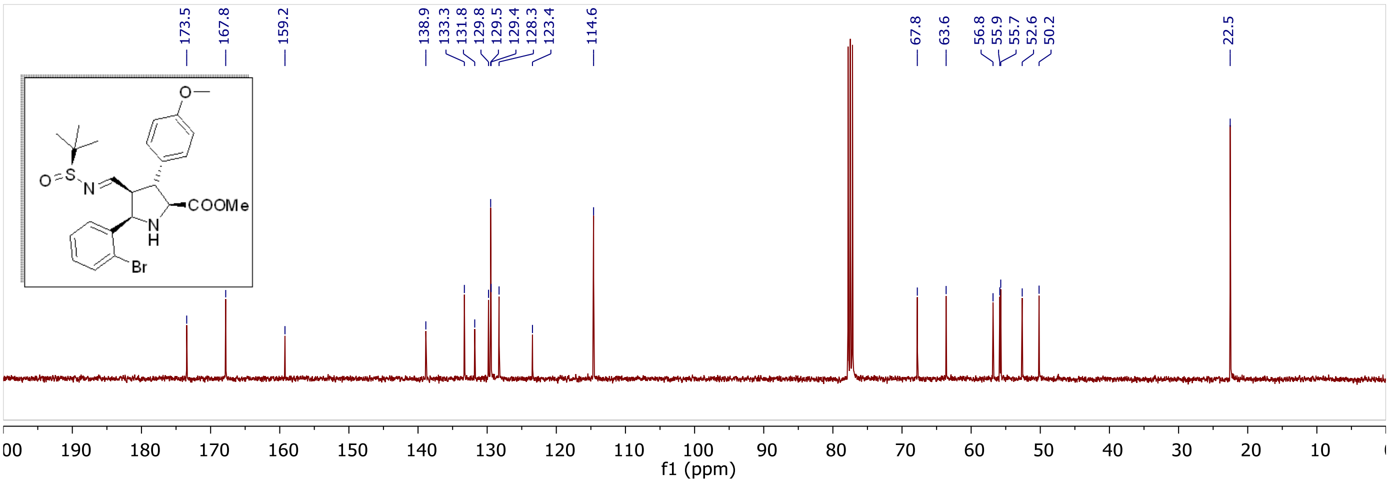

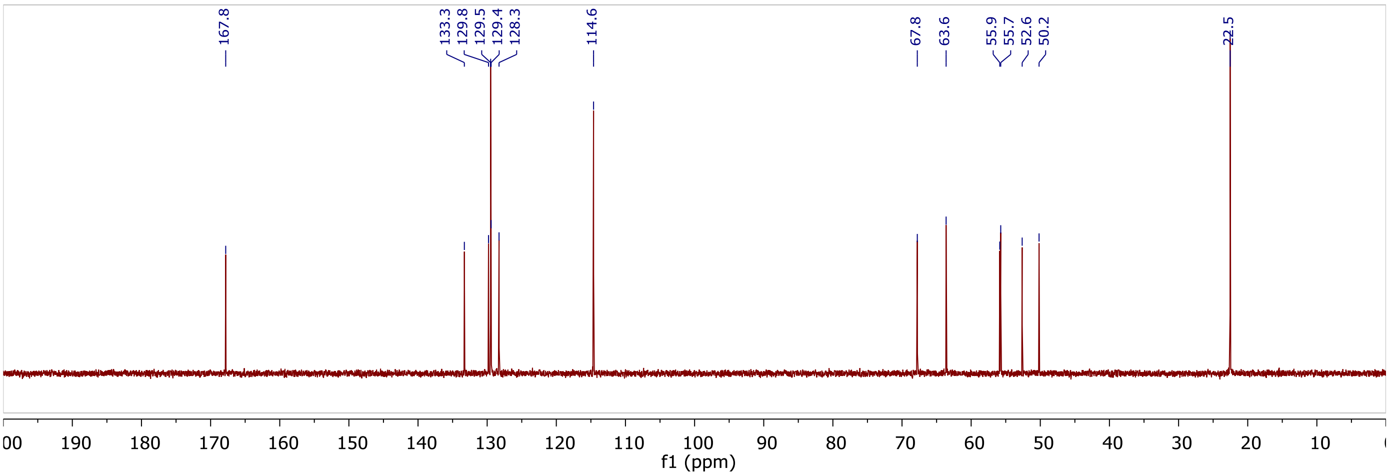

***Tert*-butyl (2*S*,3*R*,4*S*,5*R*)-5-(4-bromophenyl)-4-((*E*)-(((*S*)-*tert*-butylsulfinyl)imino)methyl)-3-(4-chlorophenyl)pyrrolidine-2-carboxylate ^{1}H NMR (400 MHz, $CDCl_3$) of 3bi**

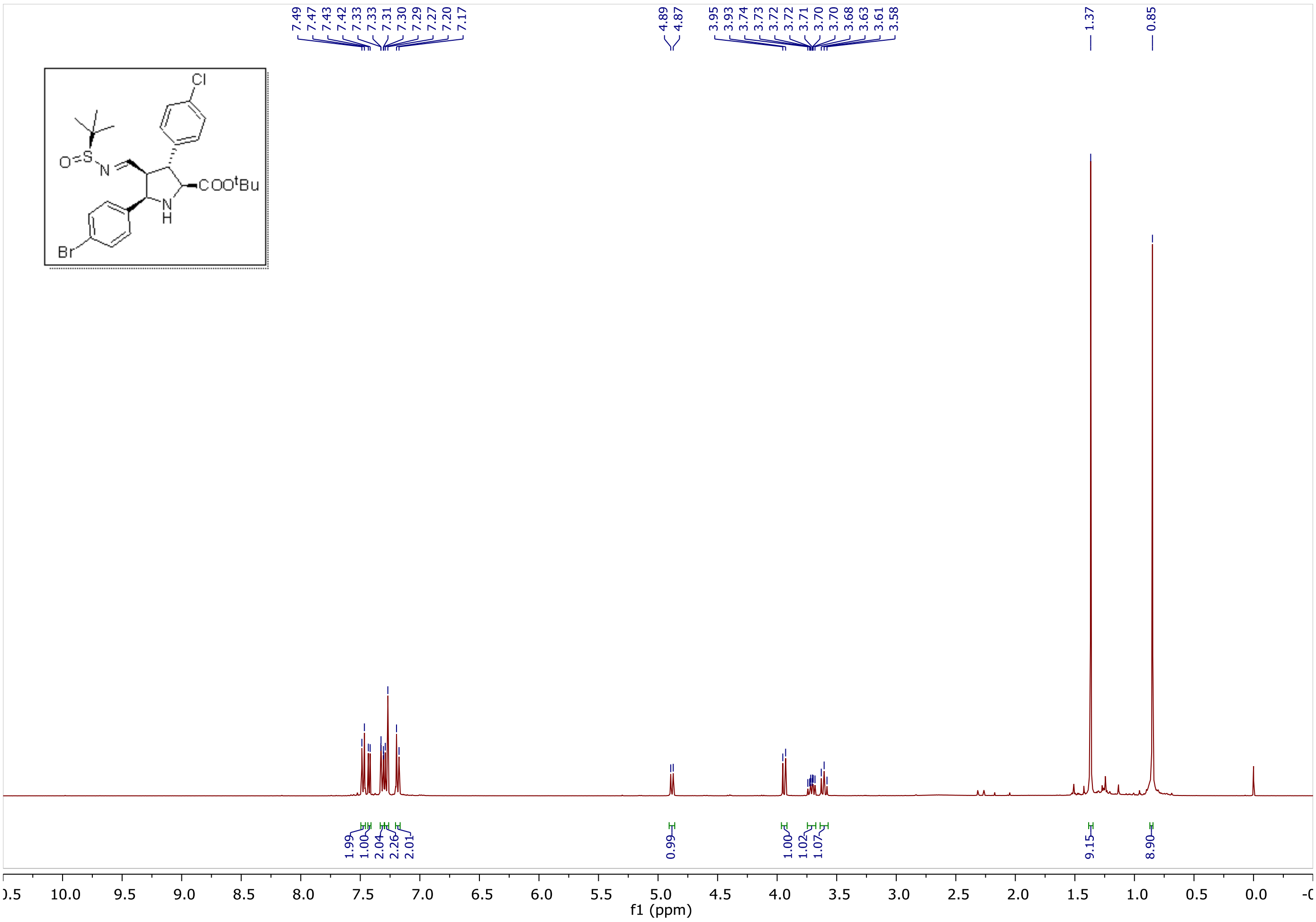

^{13}C{H} NMR (101 MHz, $CDCl_3$) of 3bi

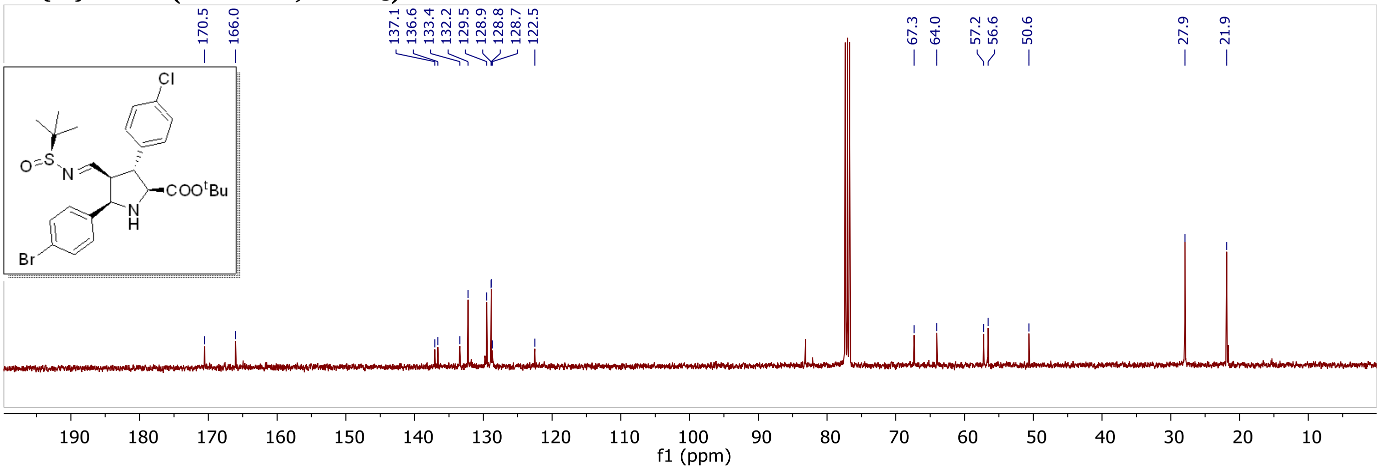

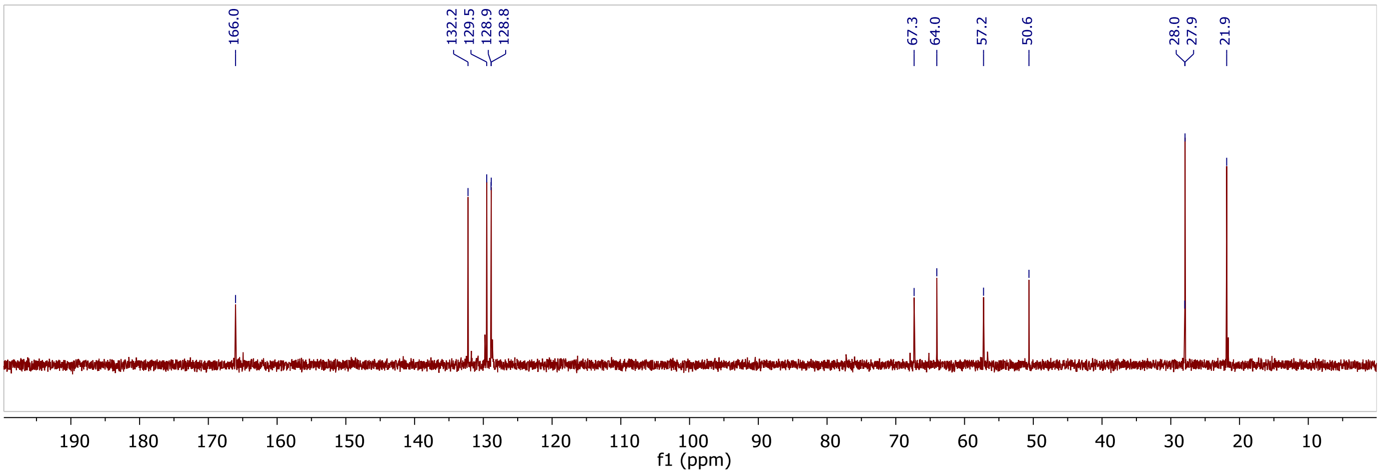

***Tert*-butyl (2*S*,3*R*,4*S*,5*R*)-5-(4-bromophenyl)-4-((*E*)-(((*S*)-*tert*-butylsulfinyl)imino)methyl)-3-(4-methoxyphenyl)pyrrolidine-2-carboxylate ^{1}H NMR (300 MHz, $CDCl_3$) of 3di**

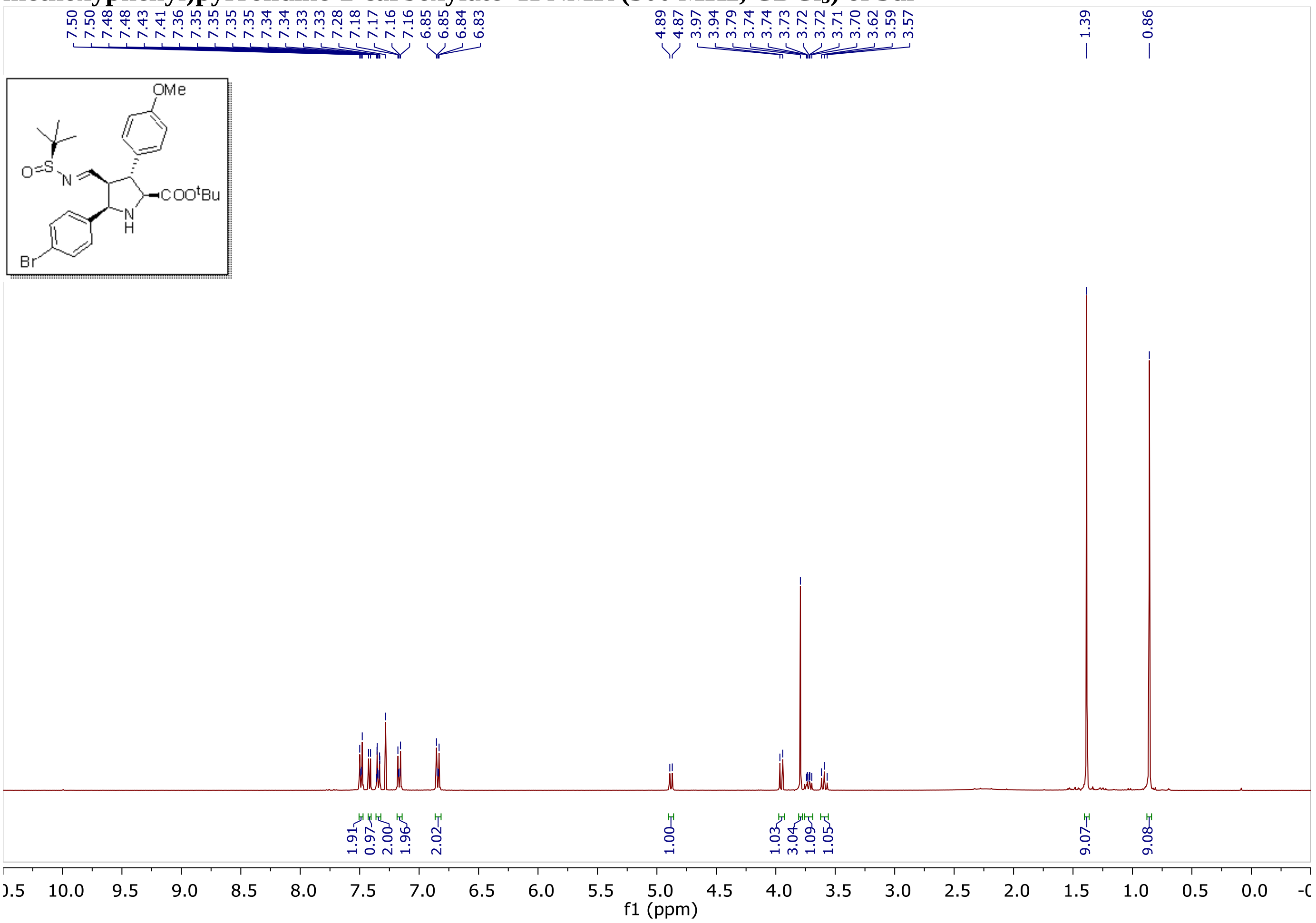

^{13}C{H} NMR (101 MHz, $CDCl_3$) of 3di

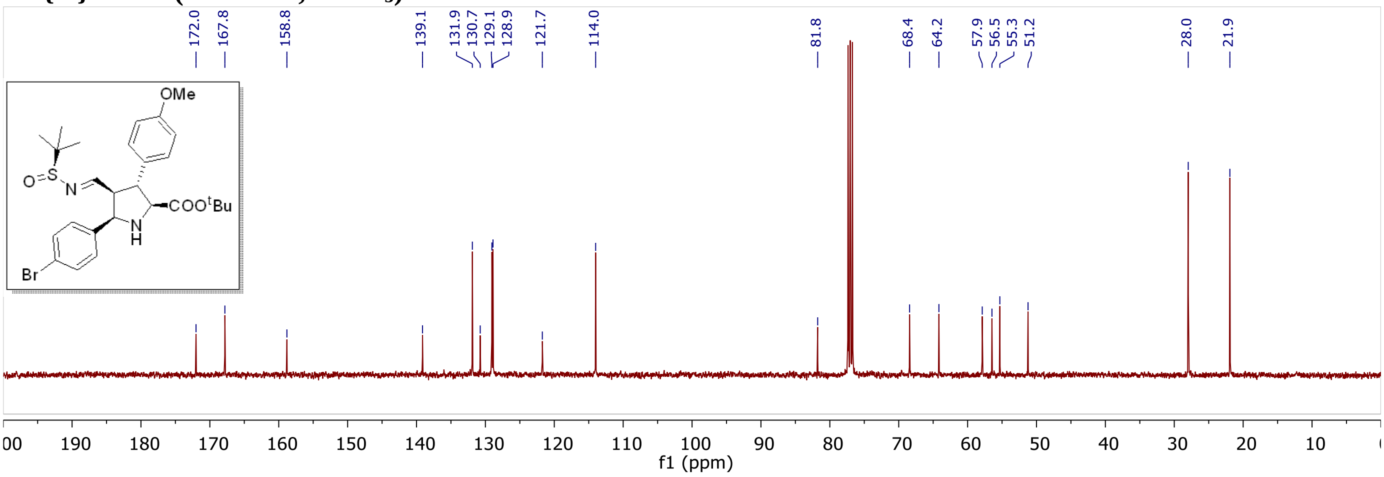

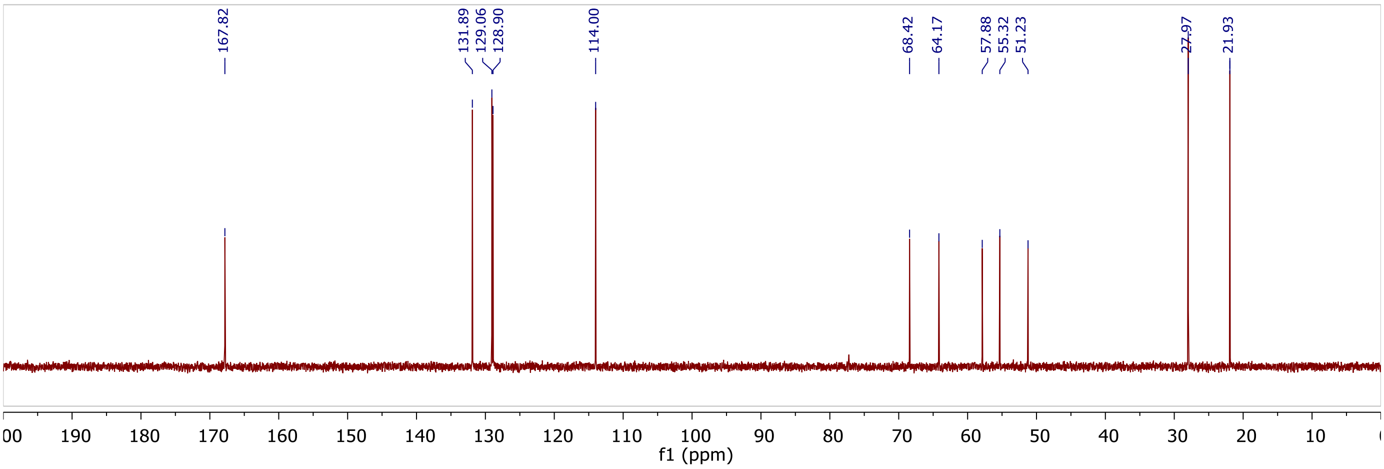

Methyl (2*S*,3*S*,4*S*,5*R*)-5-(4-bromophenyl)-4-((*E*)-(((*S*)-*tert*-butylsulfinyl)imino)methyl)-3-methylpyrrolidine-2-carboxylate.^{1}H NMR (400 MHz, $CDCl_3$) of 3ea

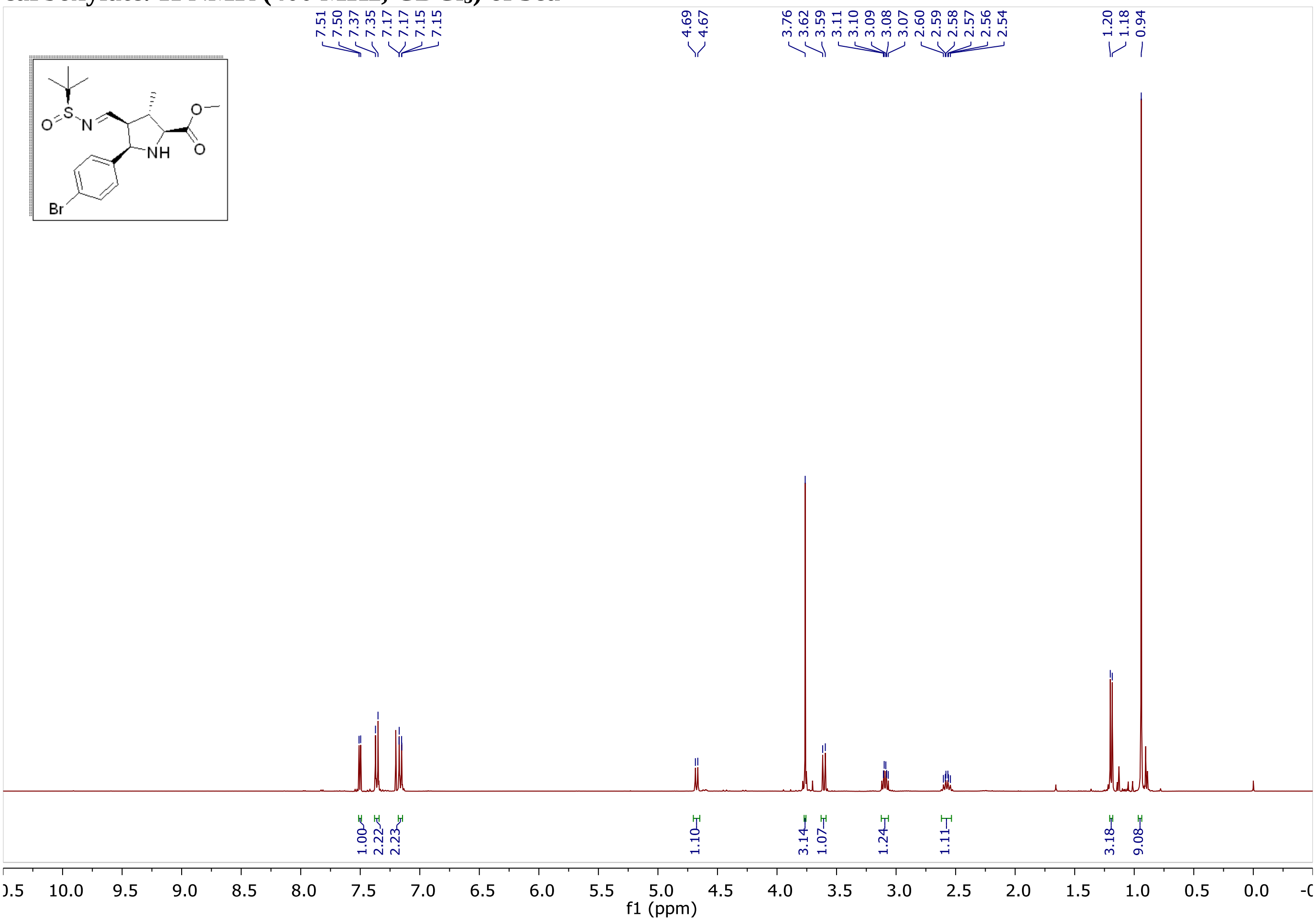

^{13}C{H} NMR (101 MHz, $CDCl_3$) of 3ea

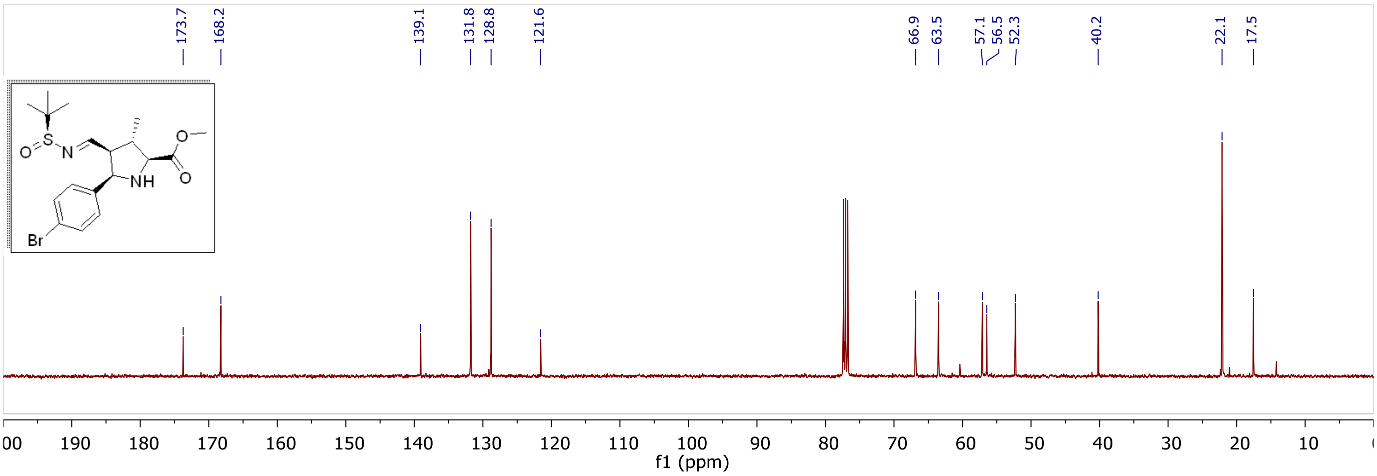

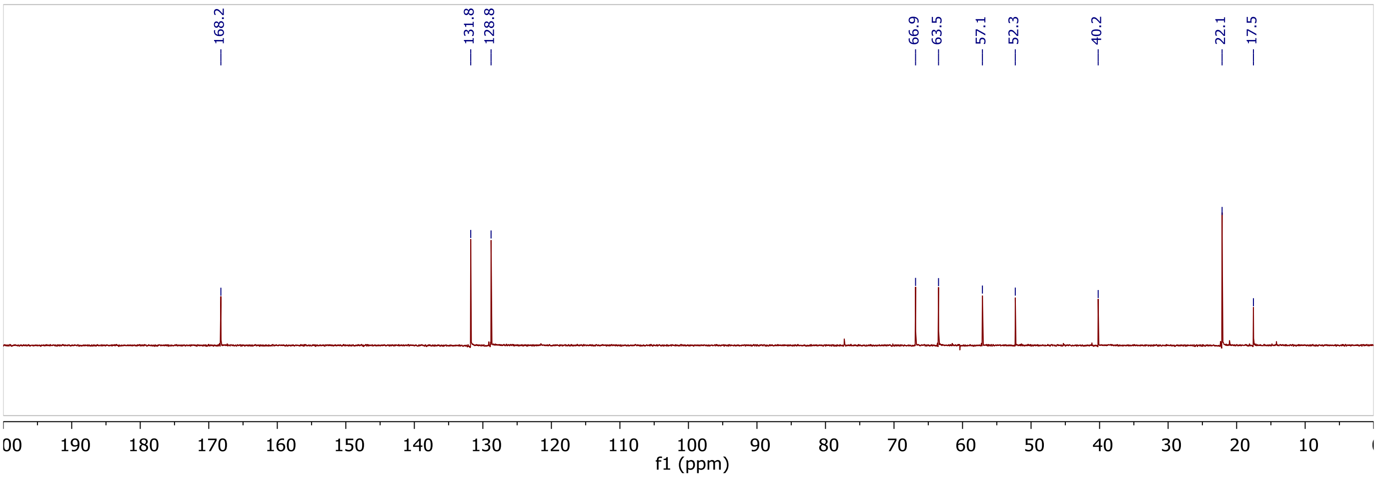

Methyl (2*S*,3*R*,4*S*,5*R*)-5-(4-bromophenyl)-4-((*E*)-(((*S*)-*tert*-butylsulfinyl)imino)methyl)-3-ethylpyrrolidine-2-carboxylate ^{1}H NMR (400 MHz, $CDCl_3$) of 3fa

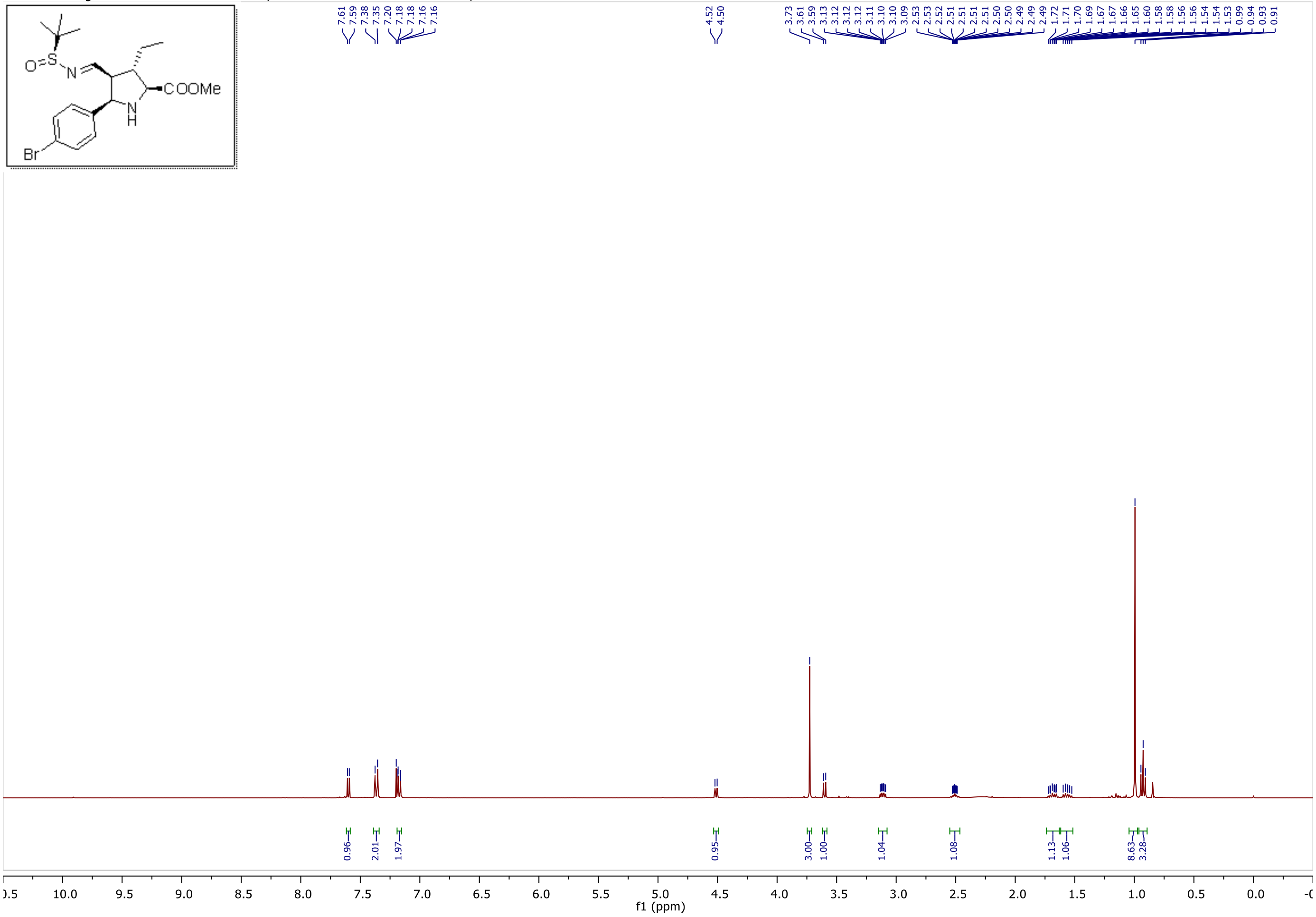

^{13}C{H} NMR (101 MHz, $CDCl_3$) of 3fa

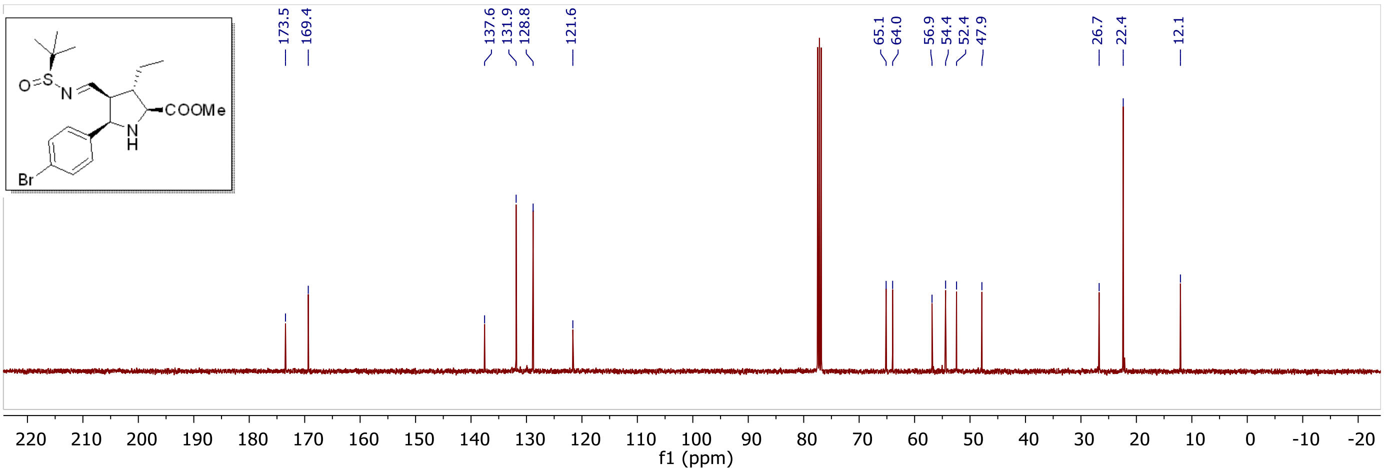

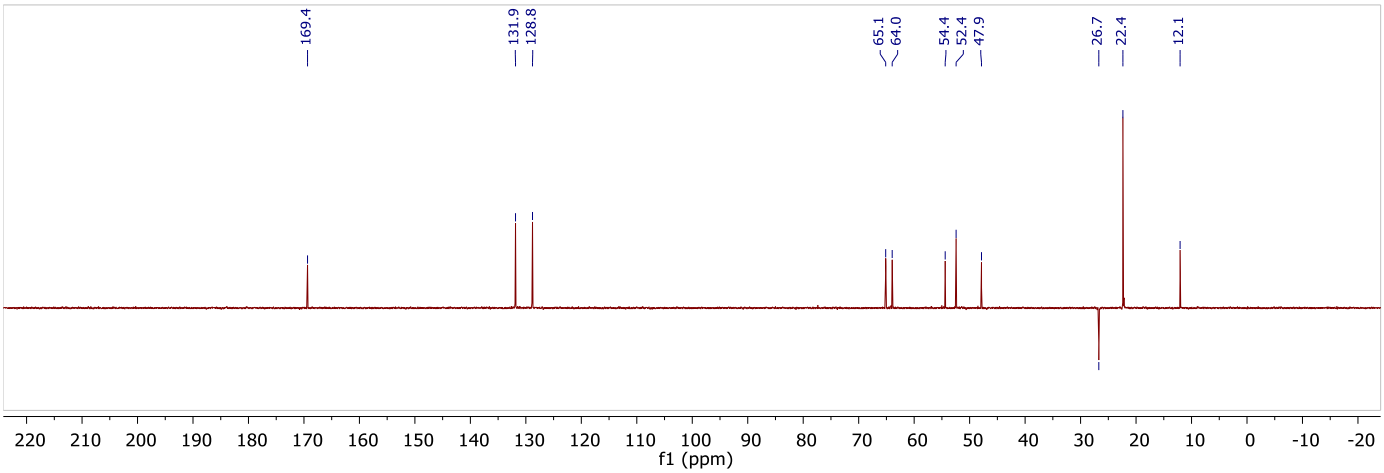

Methyl (2*S*,4*S*,5*R*)-5-(2-bromophenyl)-4-((*E*)-(((*S*)-*tert*-butylsulfinyl)imino)methyl)pyrrolidine-2-carboxylate ^{1}H NMR (300 MHz, $CDCl_3$) of 3ga

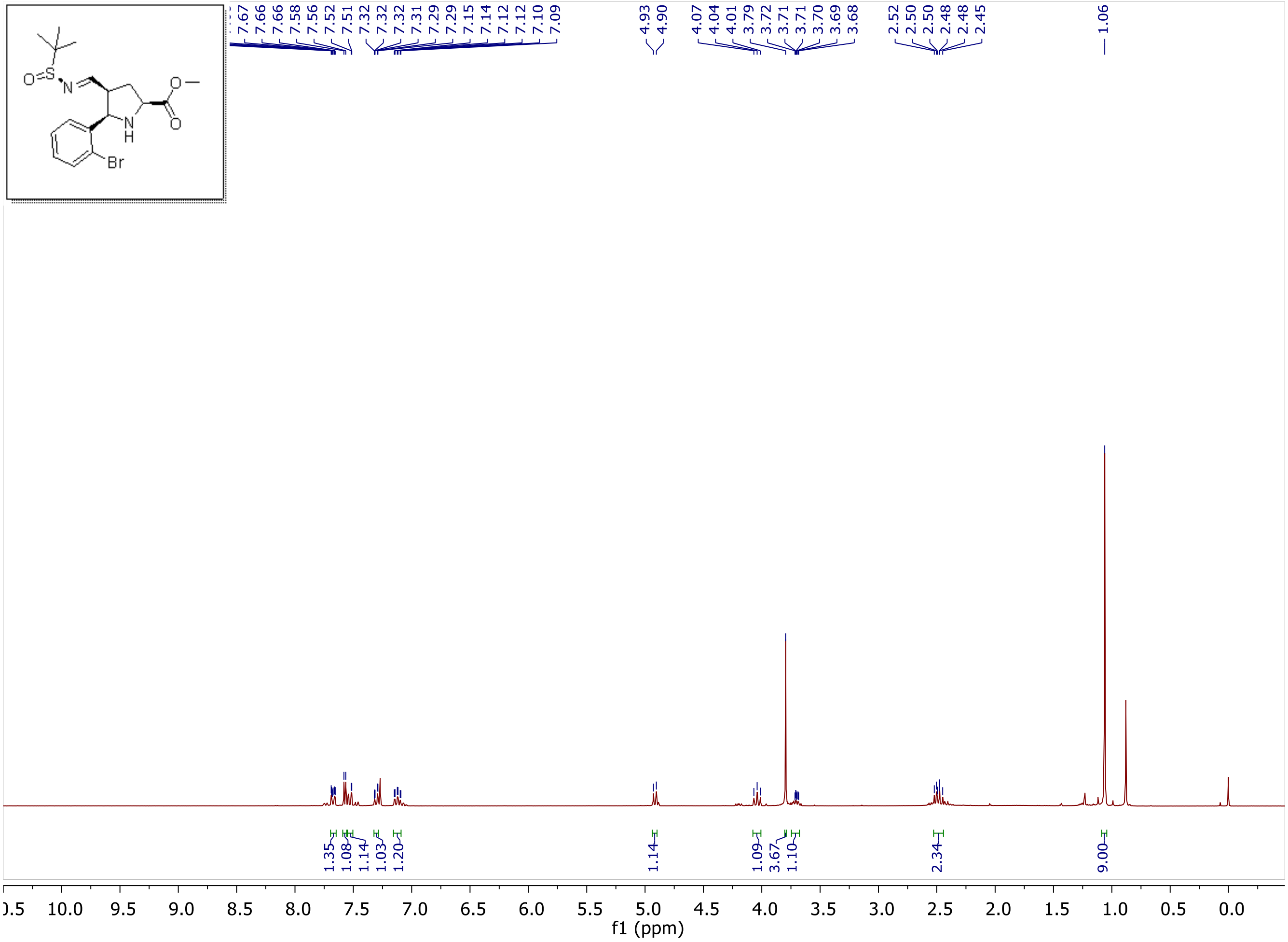

$^{13}C\{H\}$ NMR (101 MHz, $CDCl_3$) of 3ga

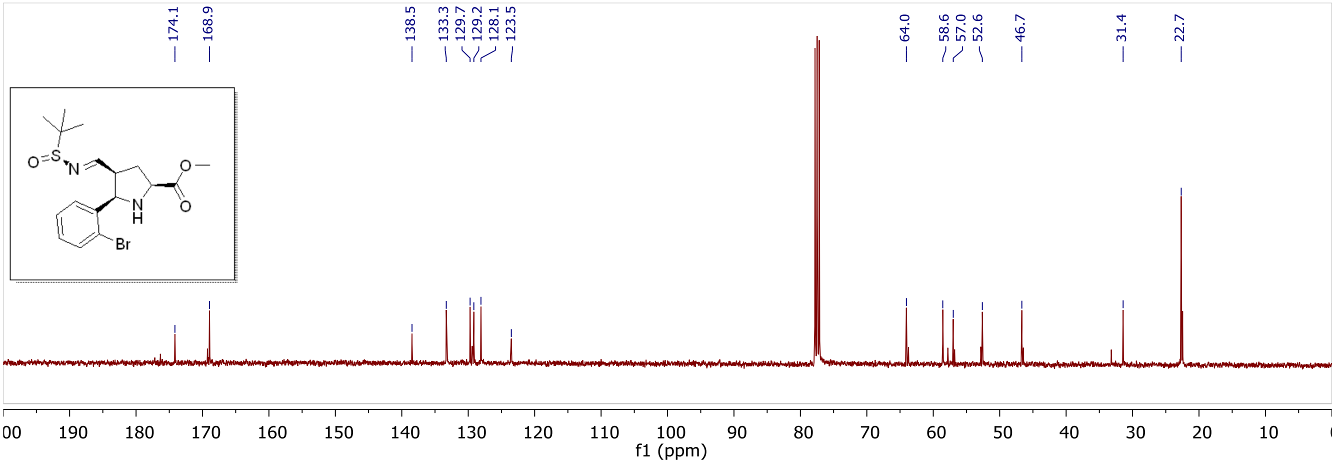

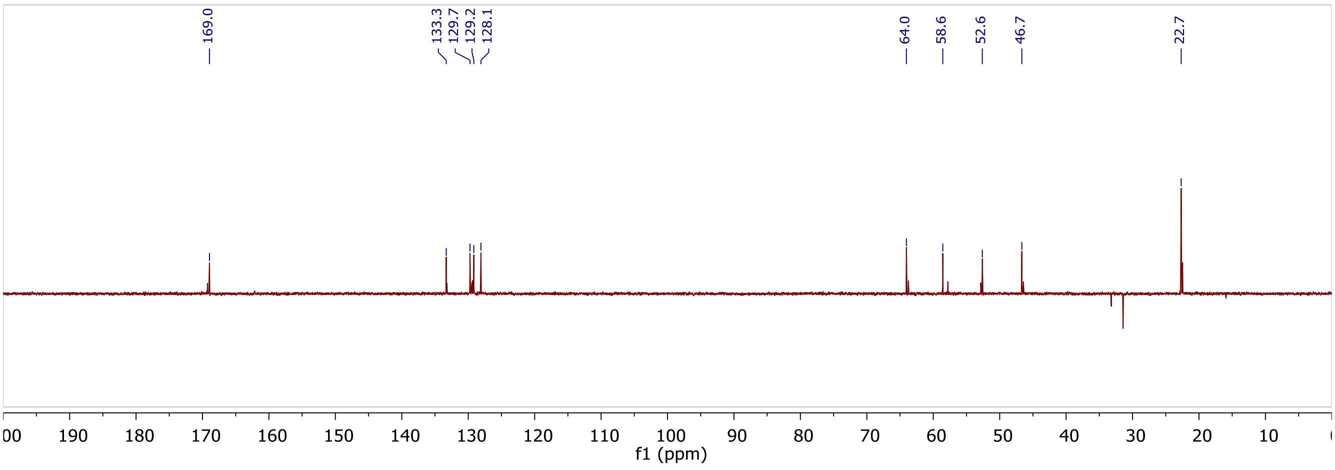

Methyl (2*S*,3*R*,4*S*,5*R*)-5-(4-bromophenyl)-4-((*E*)-1-(((*S*)-*tert*-butylsulfinyl)imino)ethyl)-3-phenylpyrrolidine-2-carboxylate ^{1}H NMR (300 MHz, $CDCl_3$) of 3ha

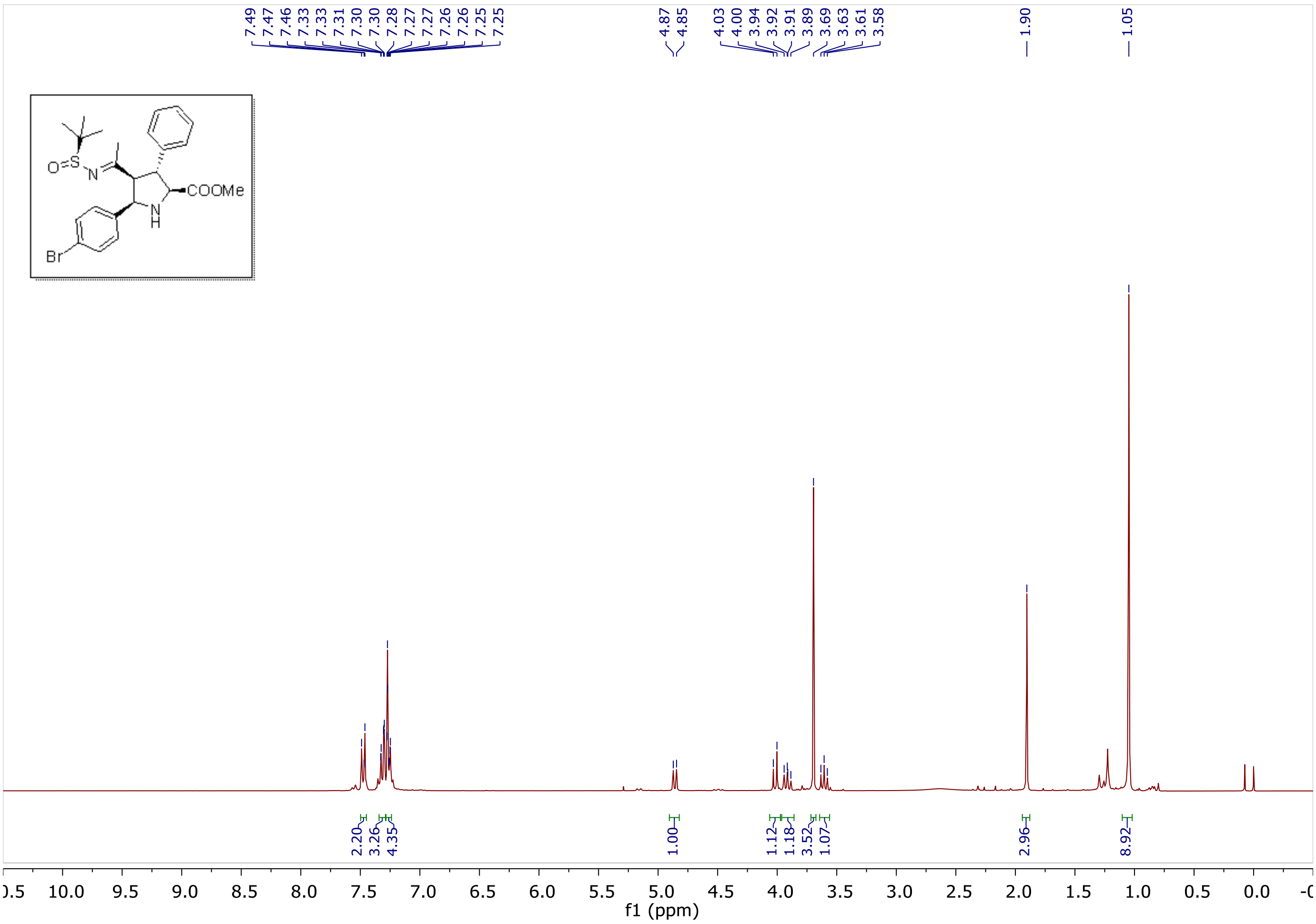

^{13}C{H} NMR (101 MHz, $CDCl_3$) of 3ha

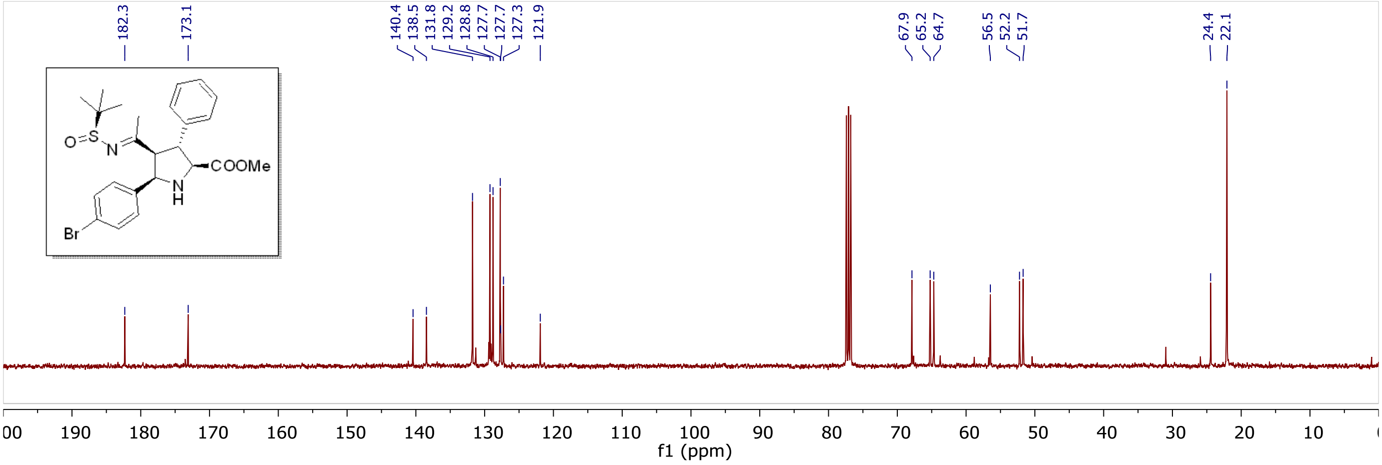

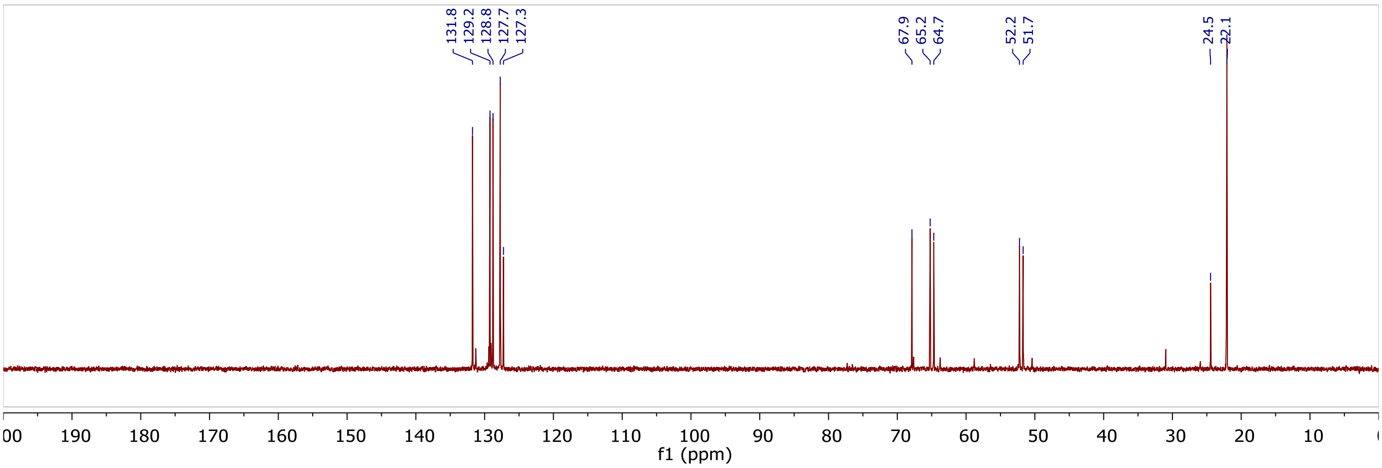

Methyl (2*S*,3*R*,4*S*,5*R*)-5-(4-bromophenyl)-4-((Z)-(((*S*)-*tert*-butylsulfinyl)imino)(phenyl)methyl)-3-phenylpyrrolidine-2-carboxylate ^{1}H NMR (400 MHz, $CDCl_3$) of 3ia

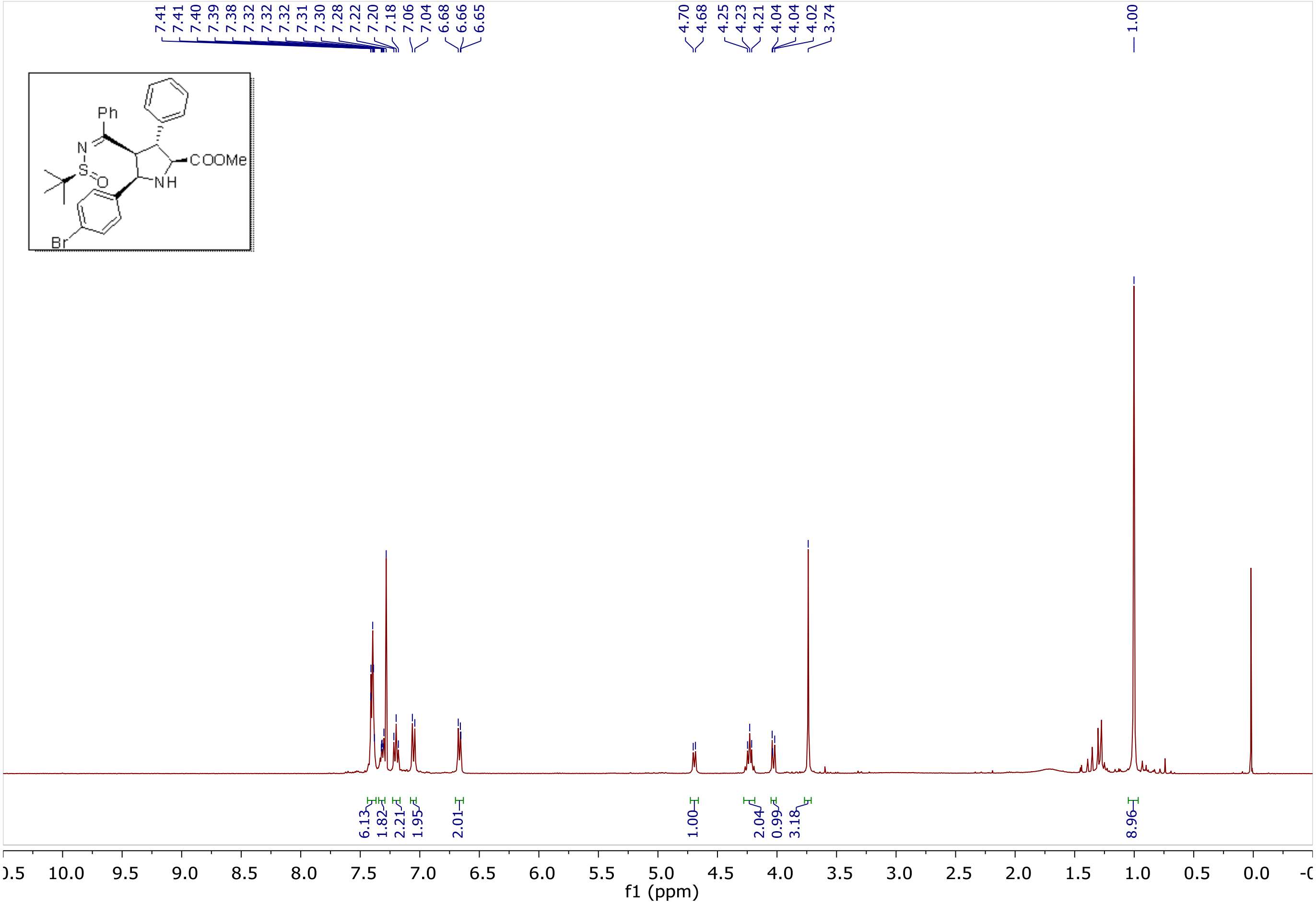

^{13}C{H} NMR (101 MHz, $CDCl_3$) of 3ia

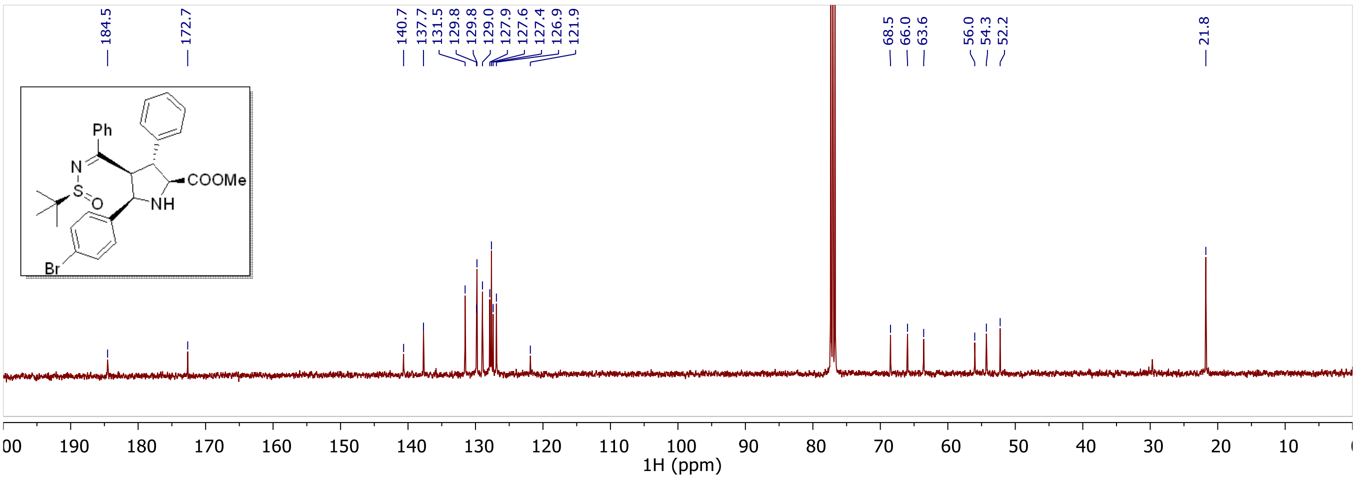

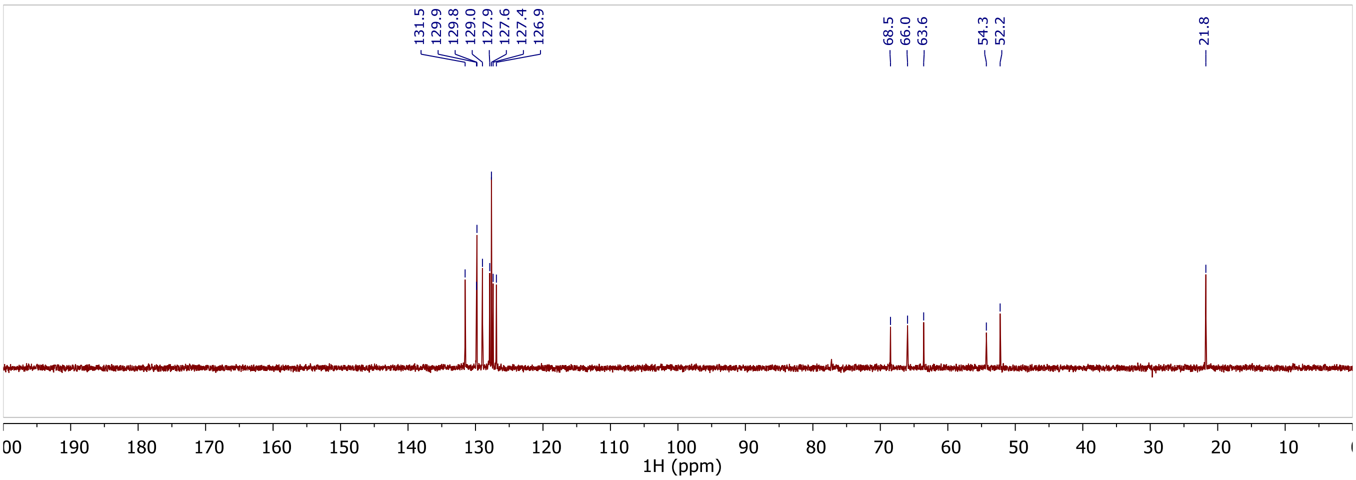

Methyl (2*S*,3*R*,4*S*,5*R*)-1-allyl-5-(2-bromophenyl)-4-((*E*)-(((*S*)-*tert*-butylsulfinyl)imino)methyl)-3-phenylpyrrolidine-2-carboxylate ^{1}H NMR (400 MHz, $CDCl_3$) of 4ac

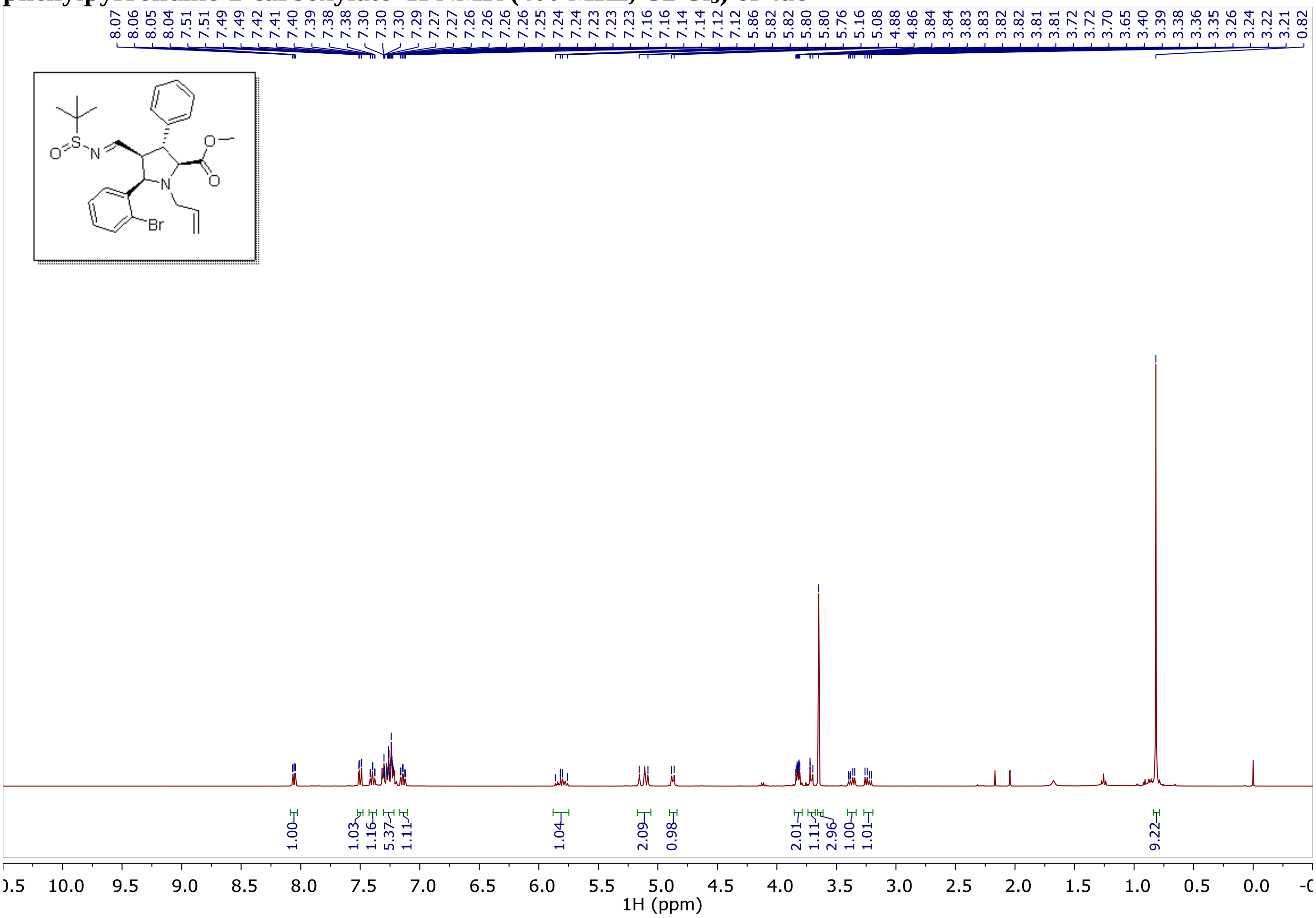

^{13}C{H} NMR (101 MHz, $CDCl_3$) of 4ac

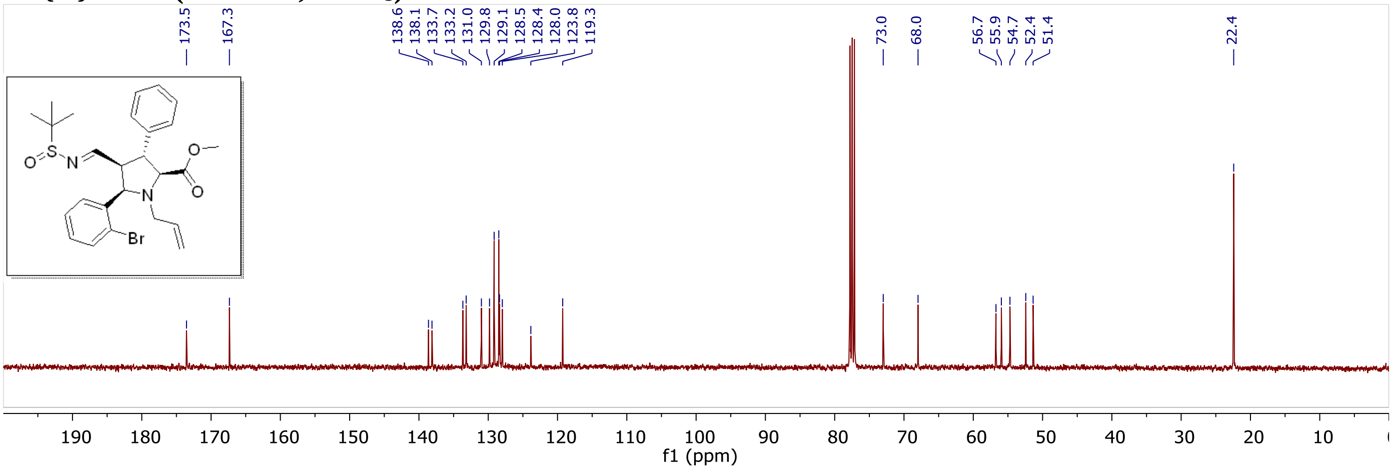

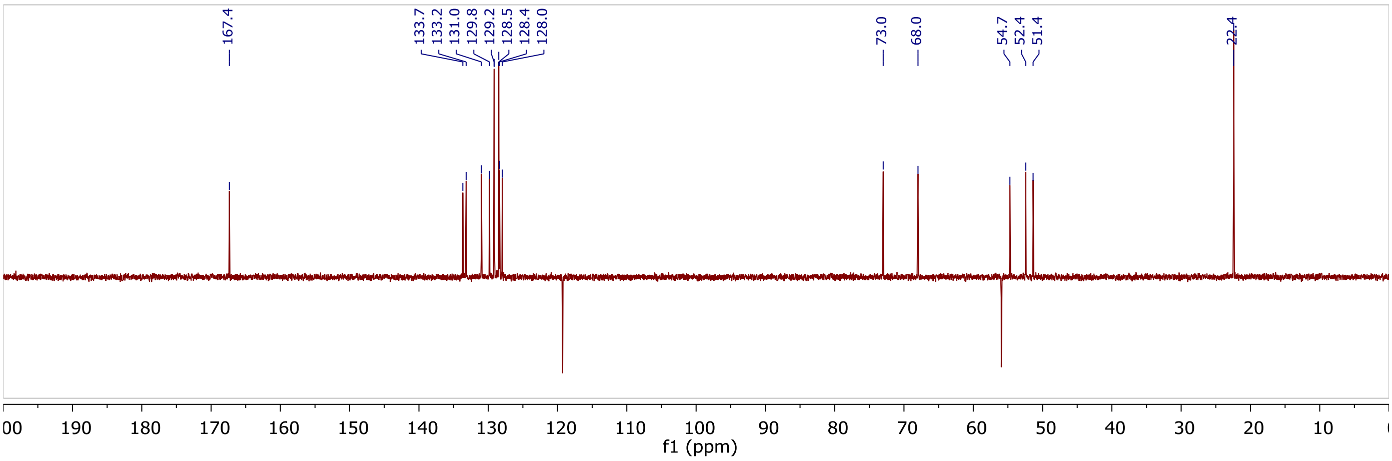

Methyl (2*S*,3*R*,4*S*,5*R*)-1-allyl-5-(2-bromophenyl)-4-((*E*)-(((*S*)-*tert*-butylsulfinyl)imino)methyl)-3-(4-methoxyphenyl)pyrrolidine-2-carboxylate ^{1}H NMR (300 MHz, $CDCl_3$) of 4dc

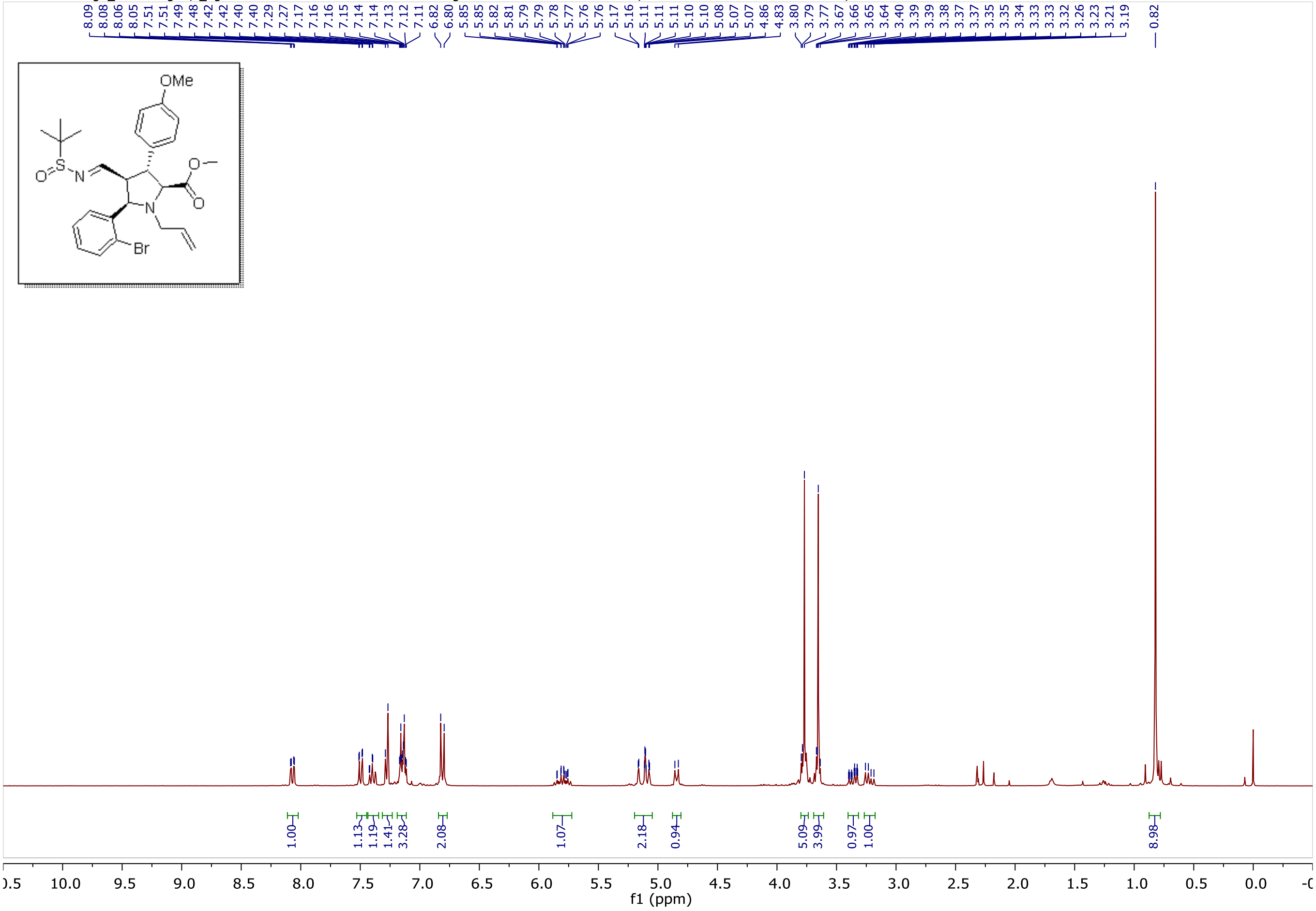

^{13}C{H} NMR (101 MHz, $CDCl_3$) of 4dc

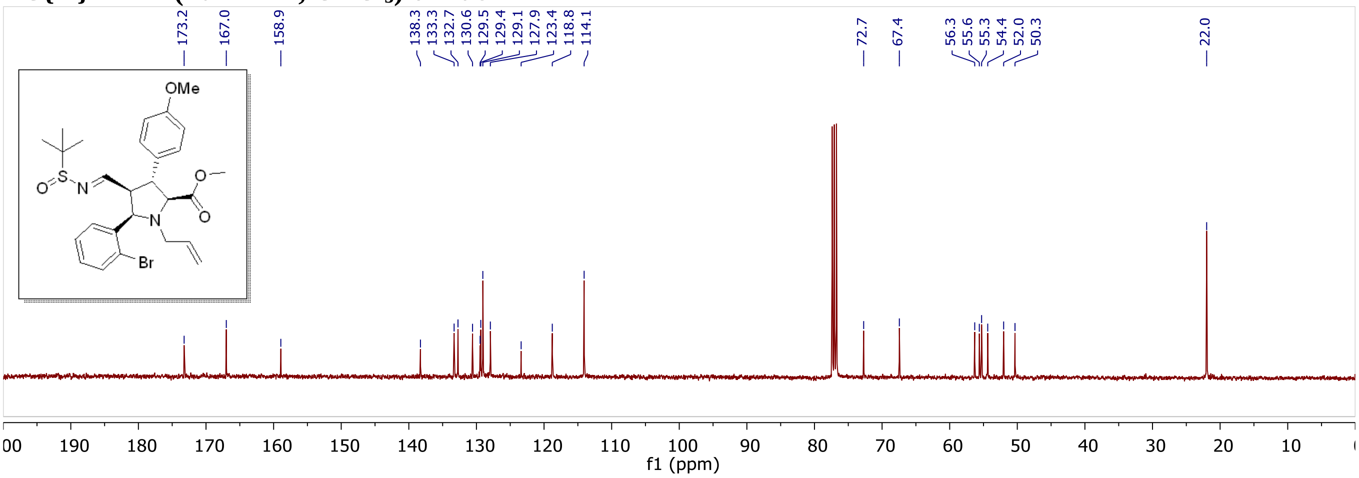

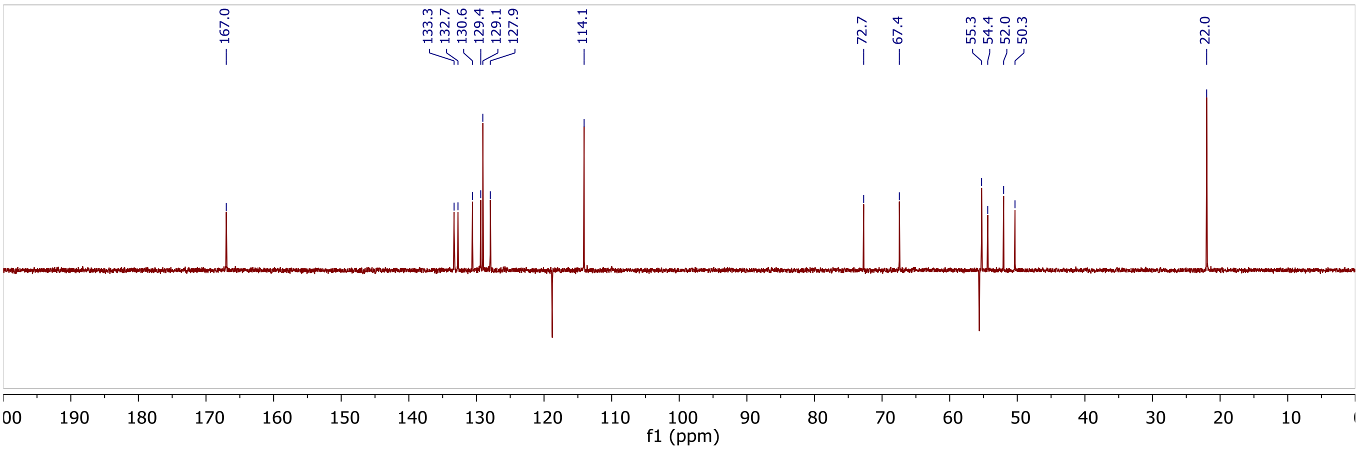

COSY ($CDCl_3$) of 4dc

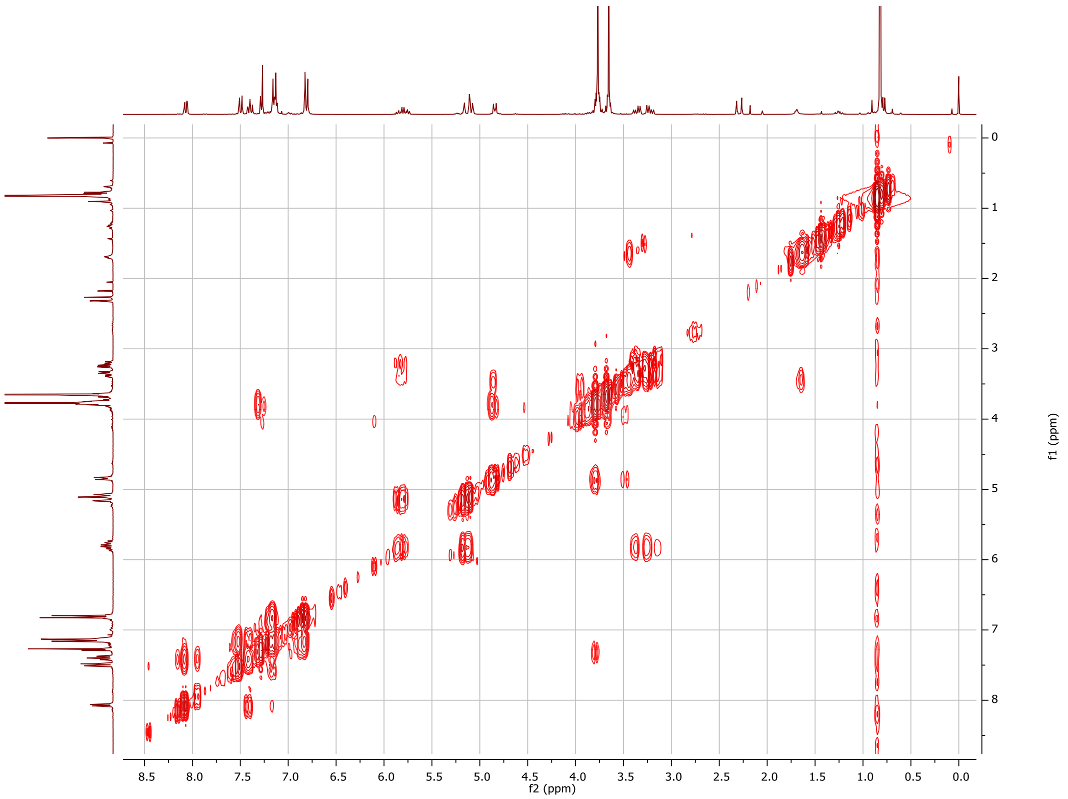

HSQC ($CDCl_3$) of 4dc

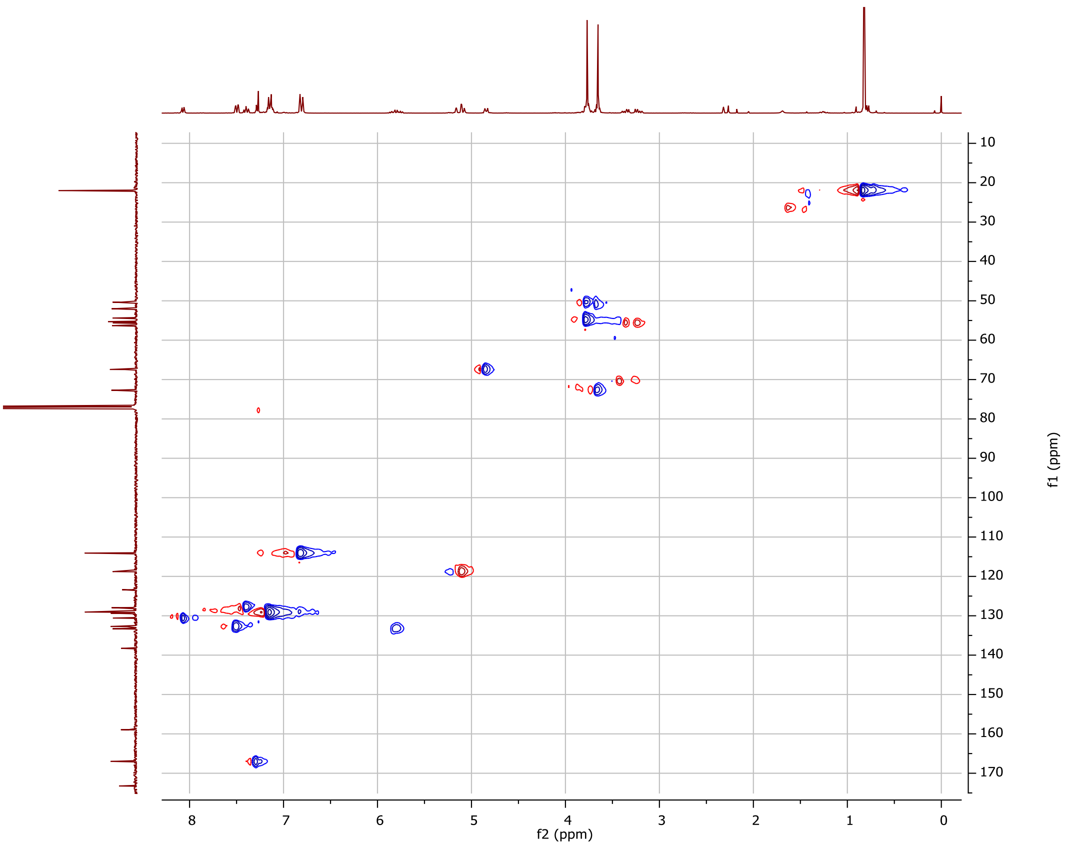

Methyl (2*S*,3*R*,4*R*,5*R*)-5-(4-bromophenyl)-4-((((*S*)-*tert*-butylsulfinyl)amino)methyl)-3-phenylpyrrolidine-2-carboxylate ^{1}H NMR (400 MHz, $CDCl_3$) of 5aa

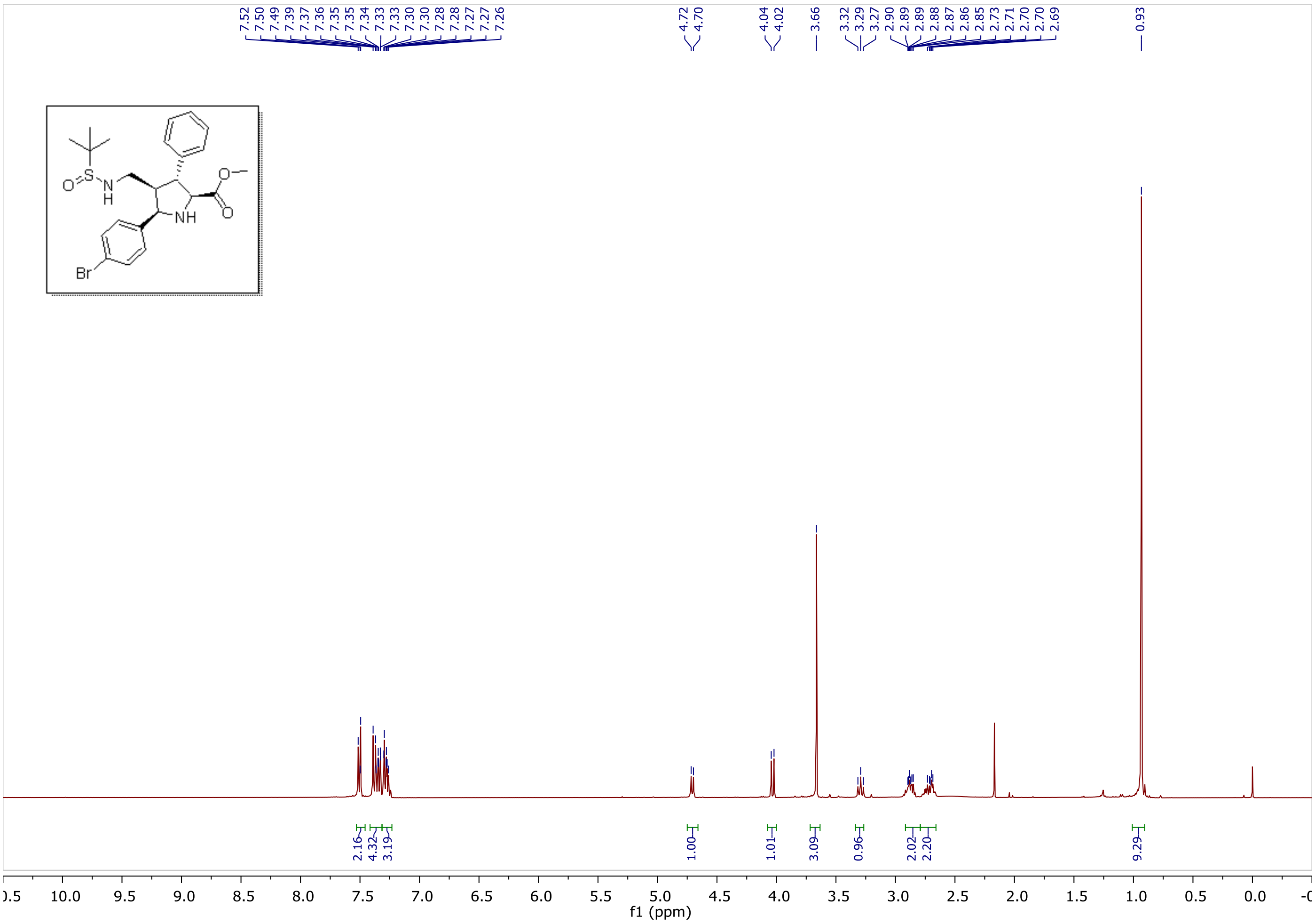

^{13}C{H} NMR (101 MHz, $CDCl_3$) of 5aa

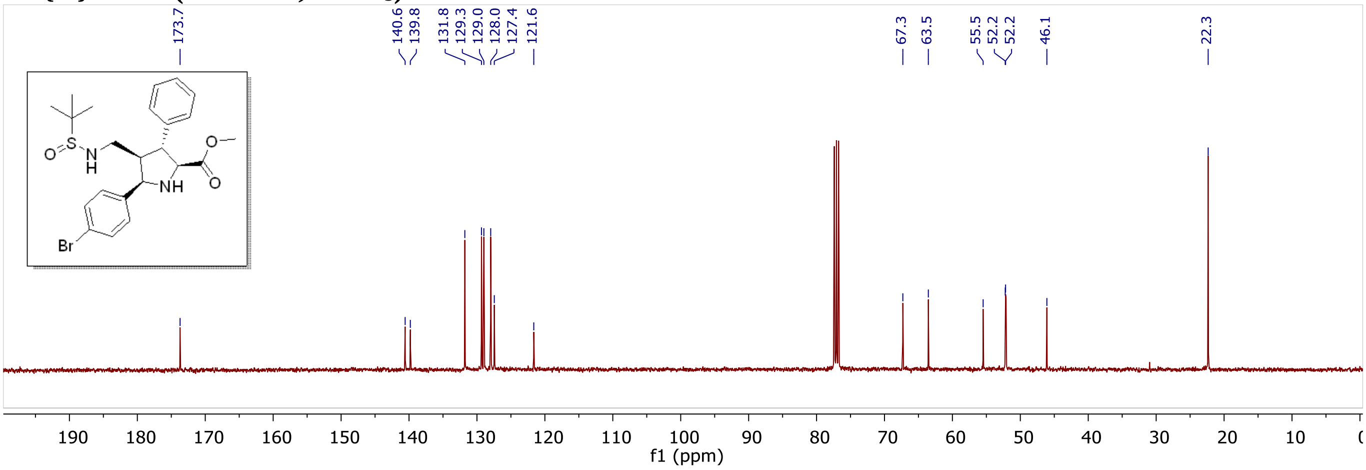

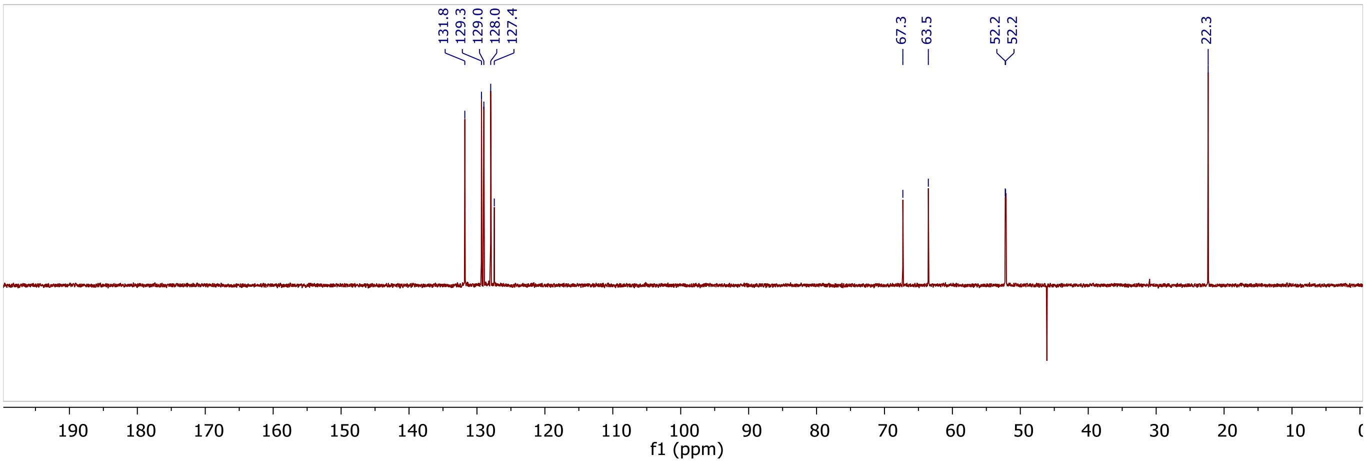

Methyl (2*S*,3*R*,4*R*,5*R*)-5-(2-bromophenyl)-4-((((*S*)-*tert*-butylsulfinyl)amino)methyl)-3-phenylpyrrolidine-2-carboxylate ^{1}H NMR (400 MHz, $CDCl_3$) of 5ac

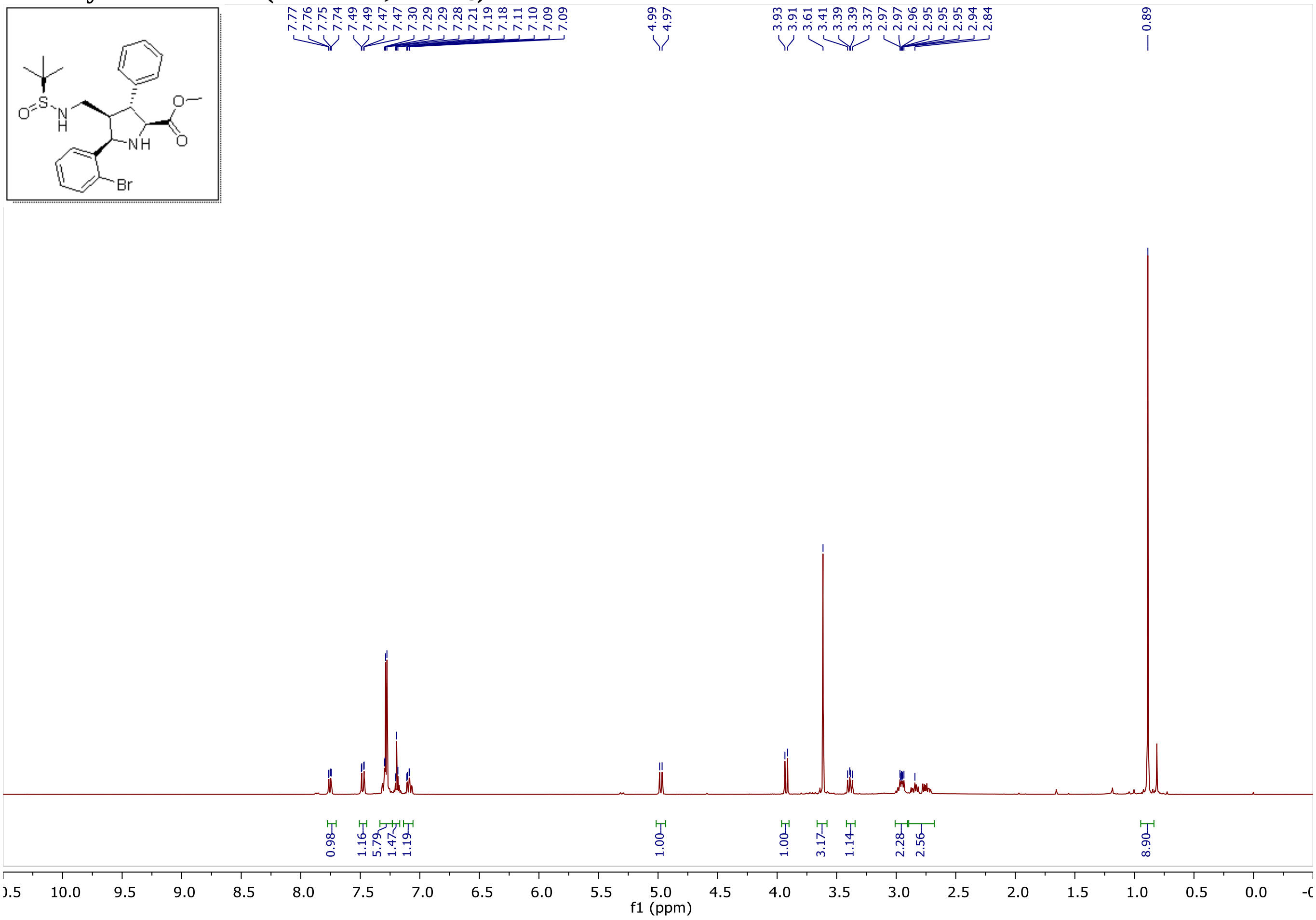

^{13}C{H} NMR (101 MHz, $CDCl_3$) of 5ac

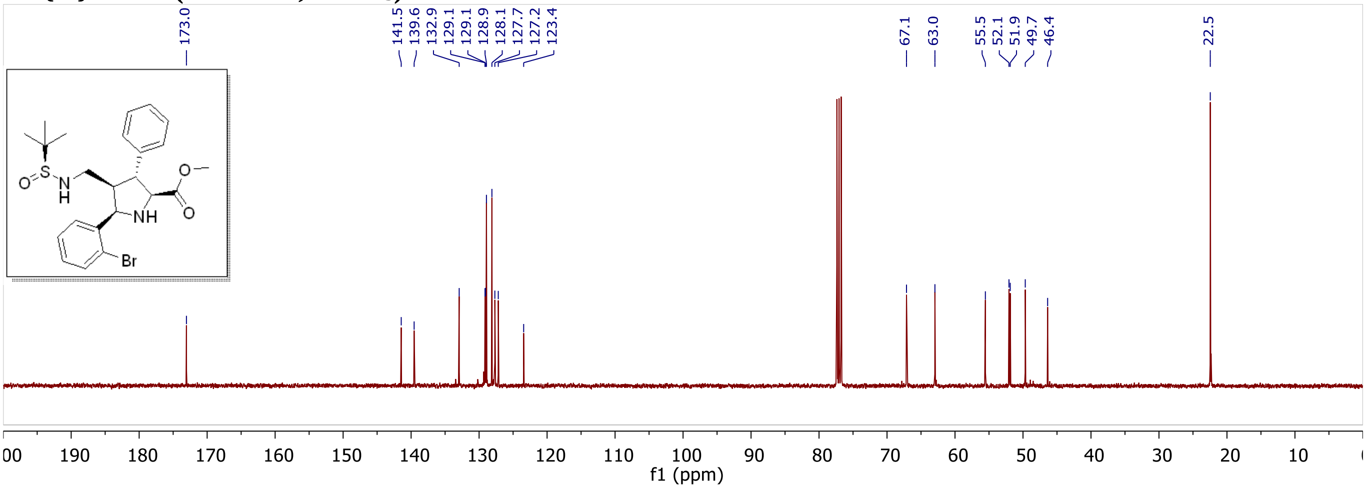

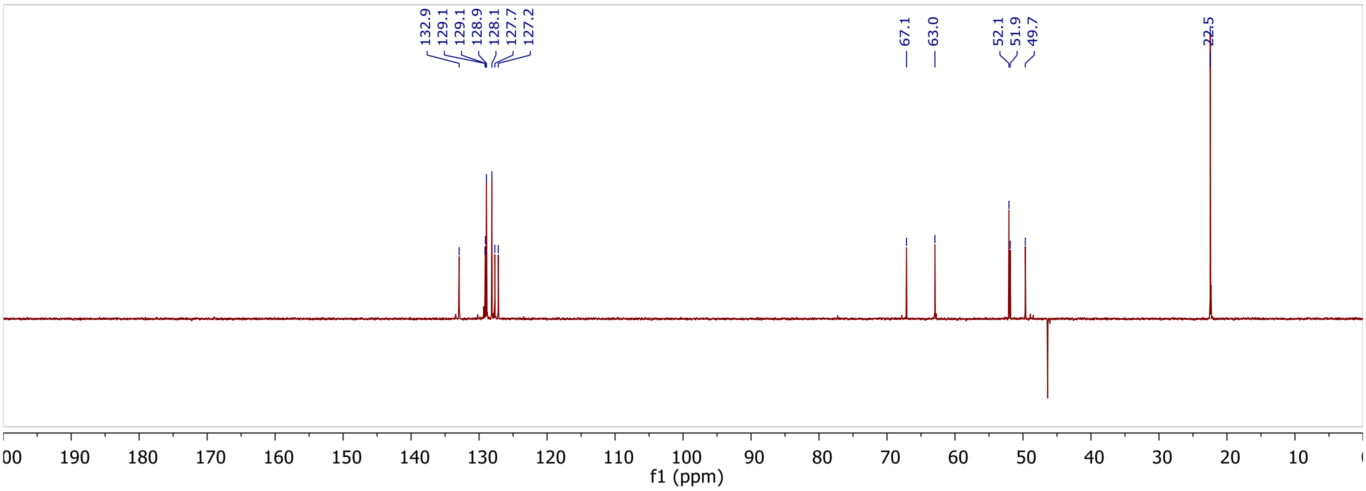

Methyl (2*S*,3*R*,4*R*,5*R*)-5-(2-bromophenyl)-4-((((*S*)-*tert*-butylsulfinyl)amino)methyl)-3-(4-methoxyphenyl)pyrrolidine-2-carboxylate ^{1}H NMR (400 MHz, $CDCl_3$) of 5dc

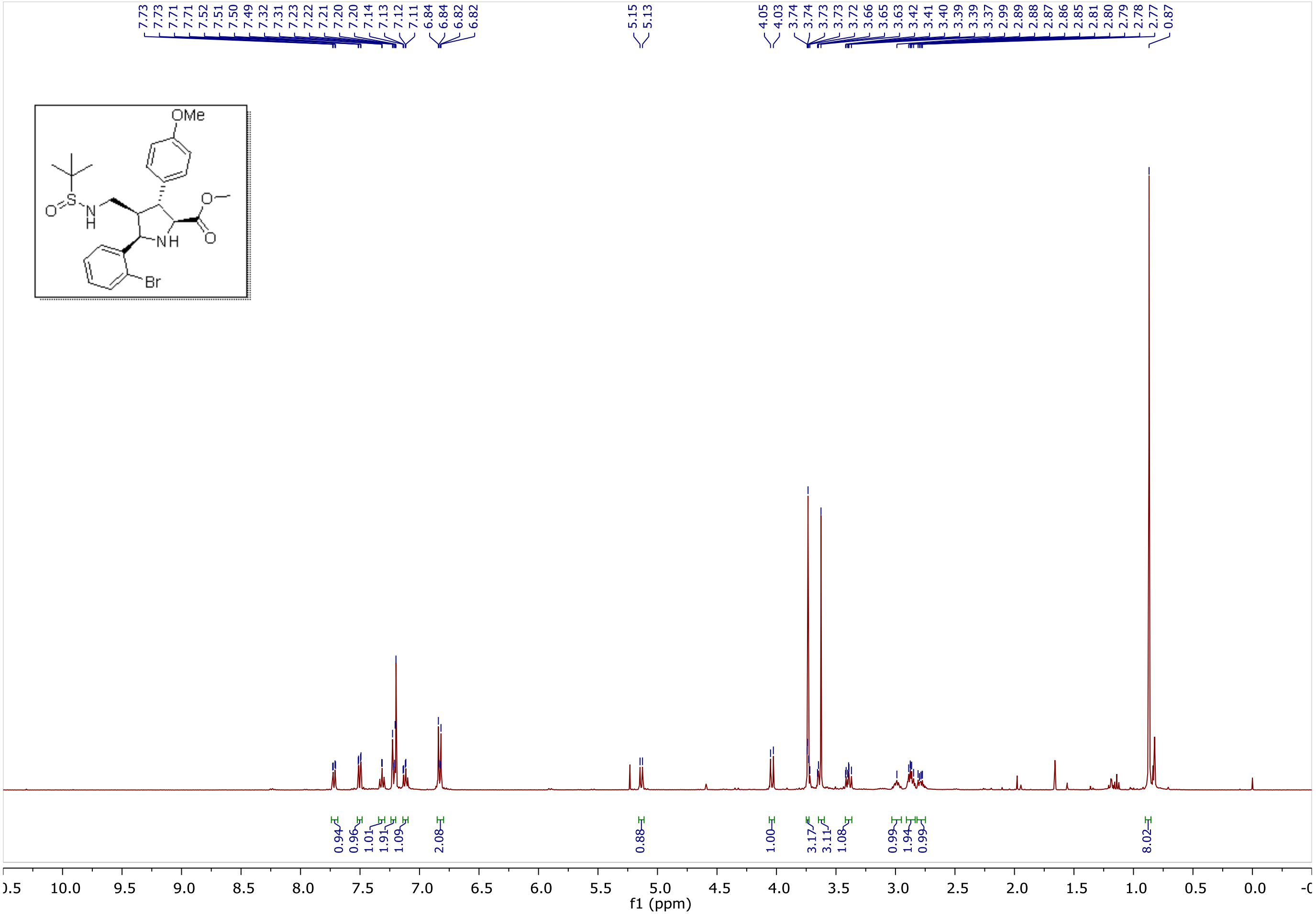

^{13}C{H} NMR (101 MHz, $CDCl_3$) of 5dc

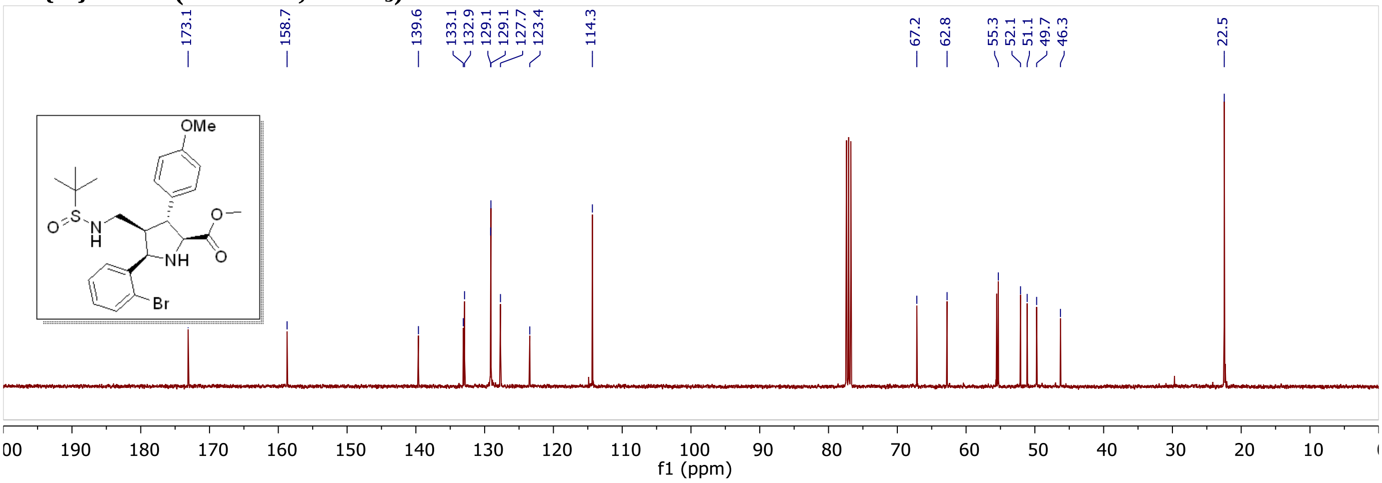

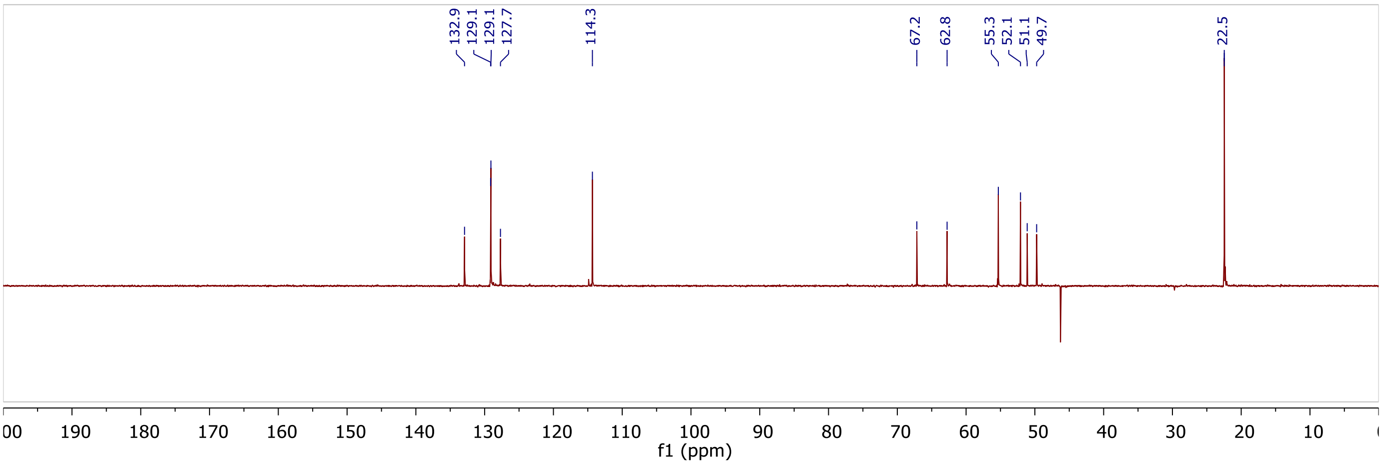

(5*R*,7*S*,8*R*)-7-(2-bromophenyl)-8-phenyl-3,6-diazabicyclo[3.2.1]octan-4-one ^{1}H NMR (400 MHz, $CDCl_3$) of 6ac

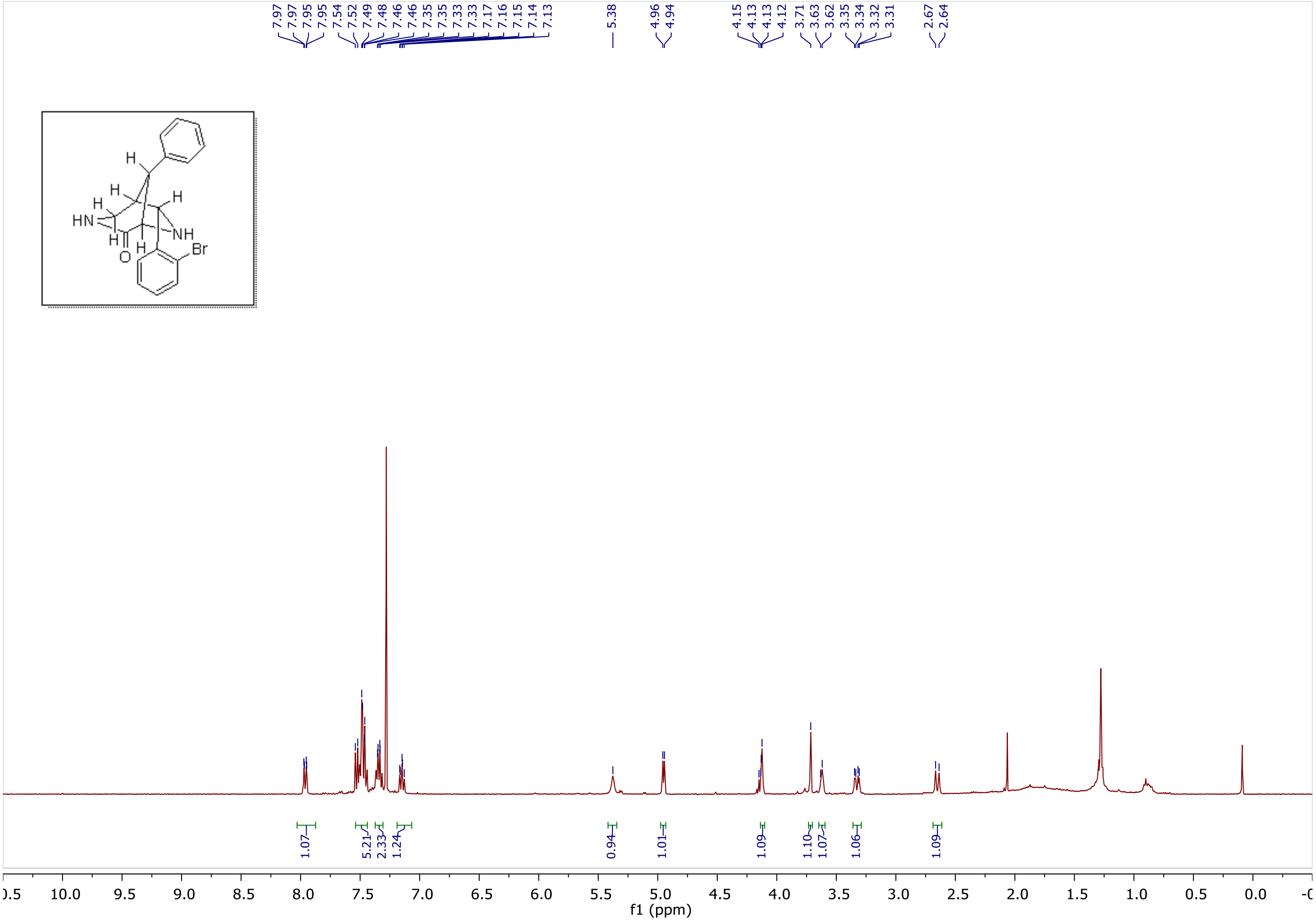

^{13}C{H} NMR (101 MHz, $CDCl_3$) of 6ac

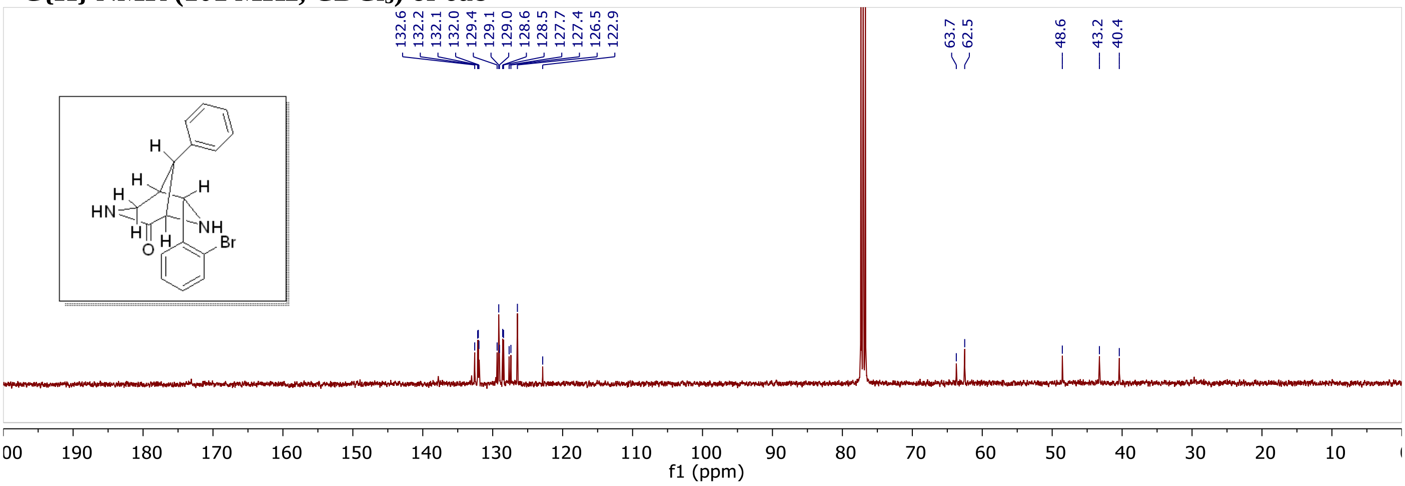

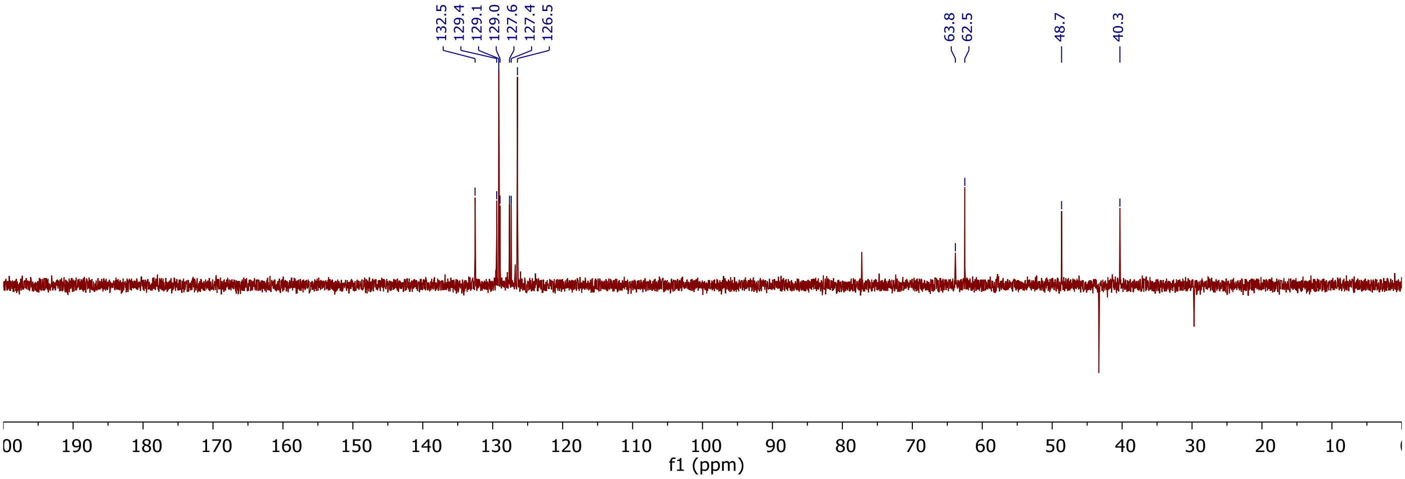

COSY ($CDCl_3$) of 6ac

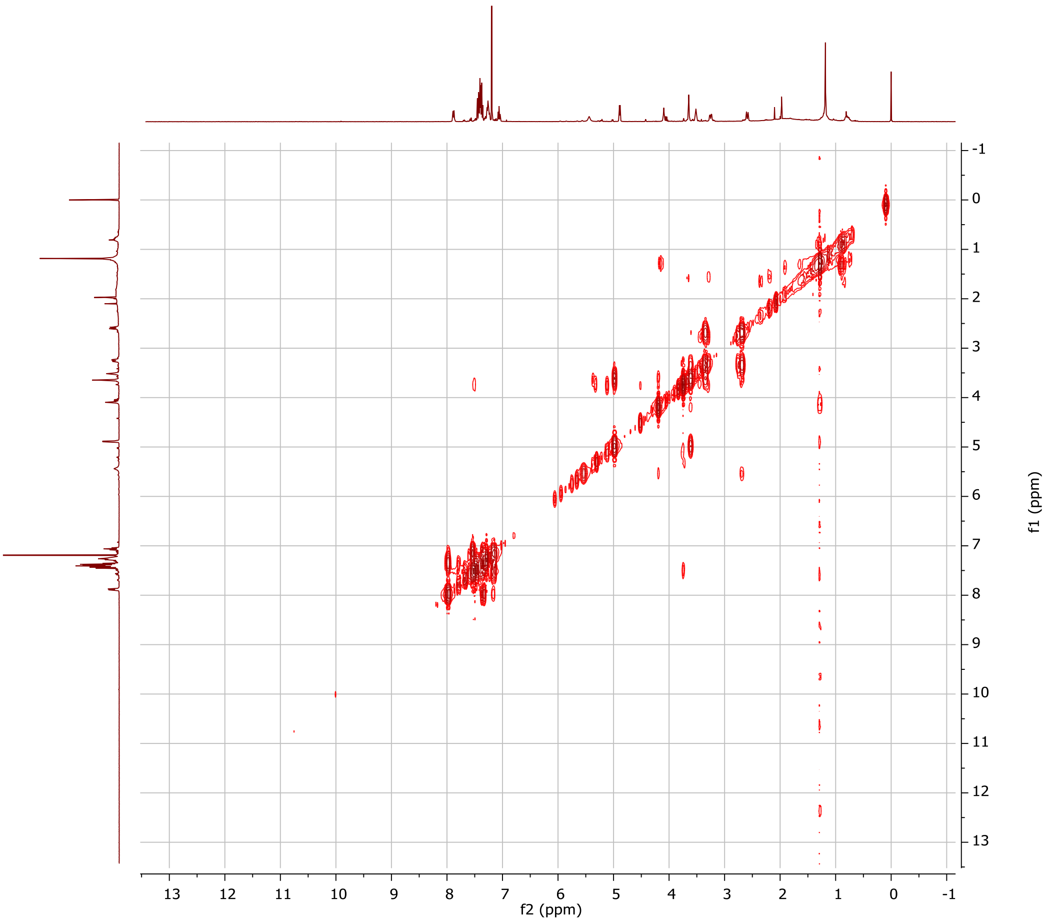

HSQC ($CDCl_3$) of 6ac

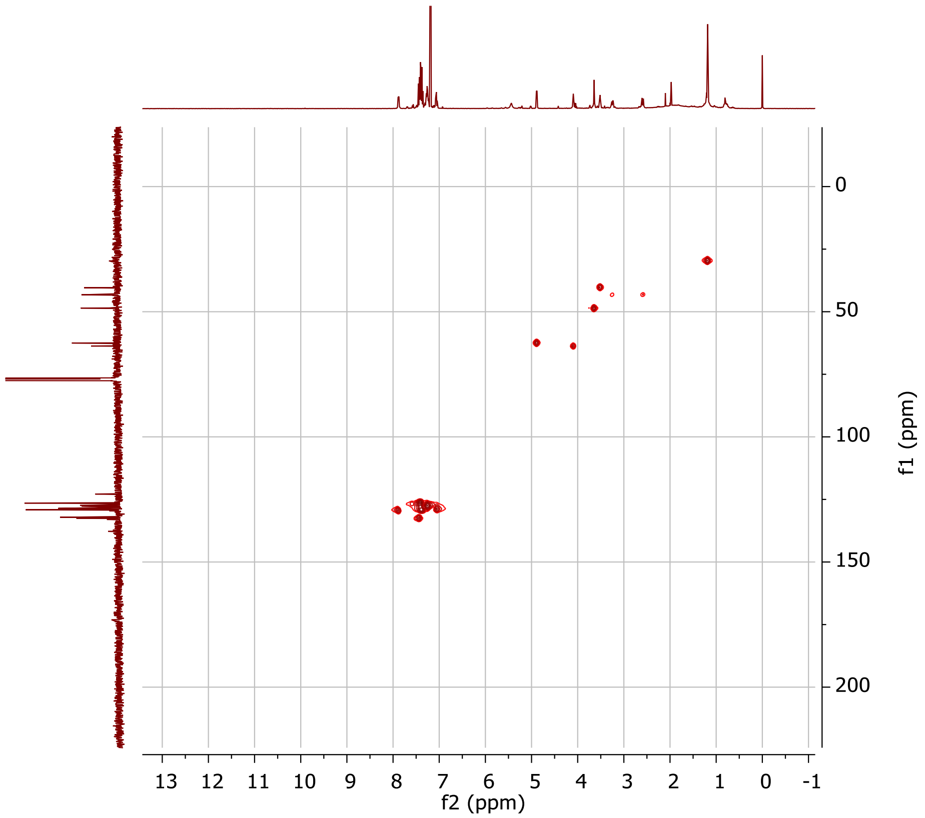

(5*R*,7*S*,8*R*)-7-(4-bromophenyl)-8-phenyl-3,6-diazabicyclo[3.2.1]octan-4-one ^{1}H NMR (400 MHz, $CDCl_3$) of 6aa

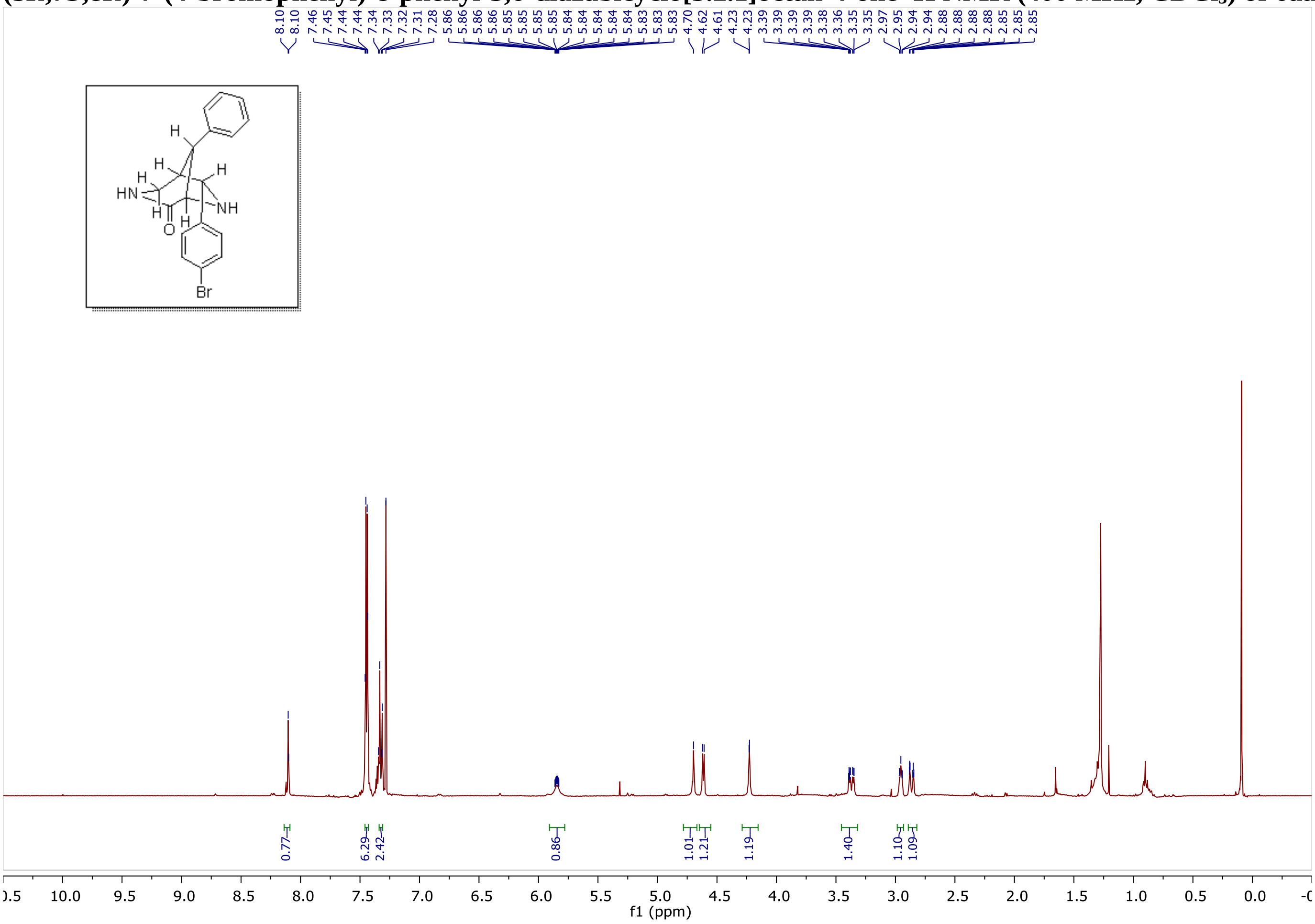

^{13}C{H} NMR (101 MHz, $CDCl_3$) of 6aa

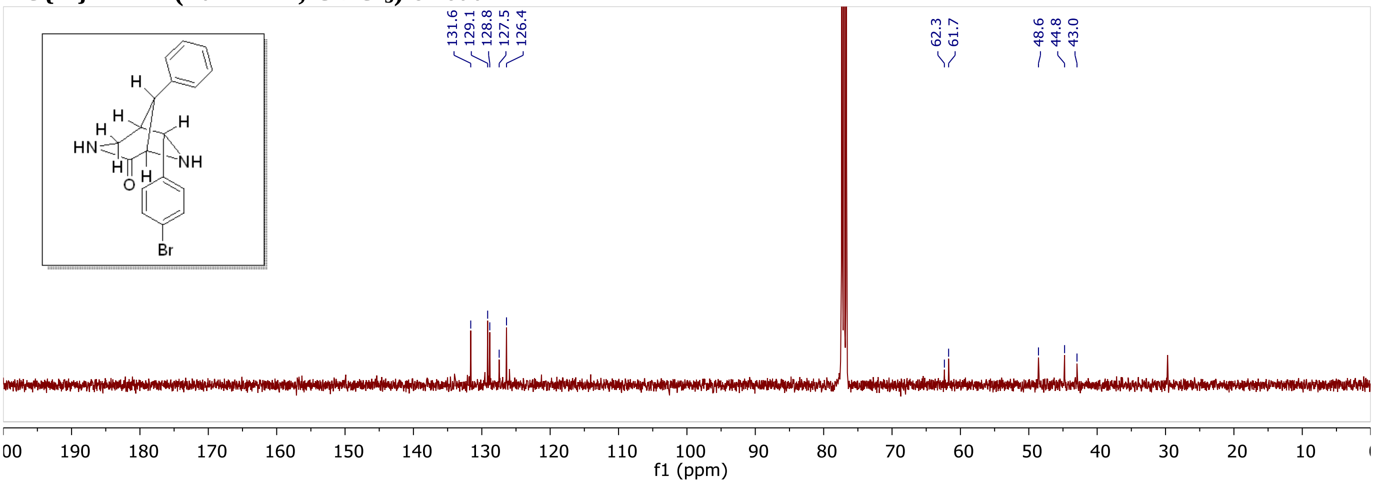

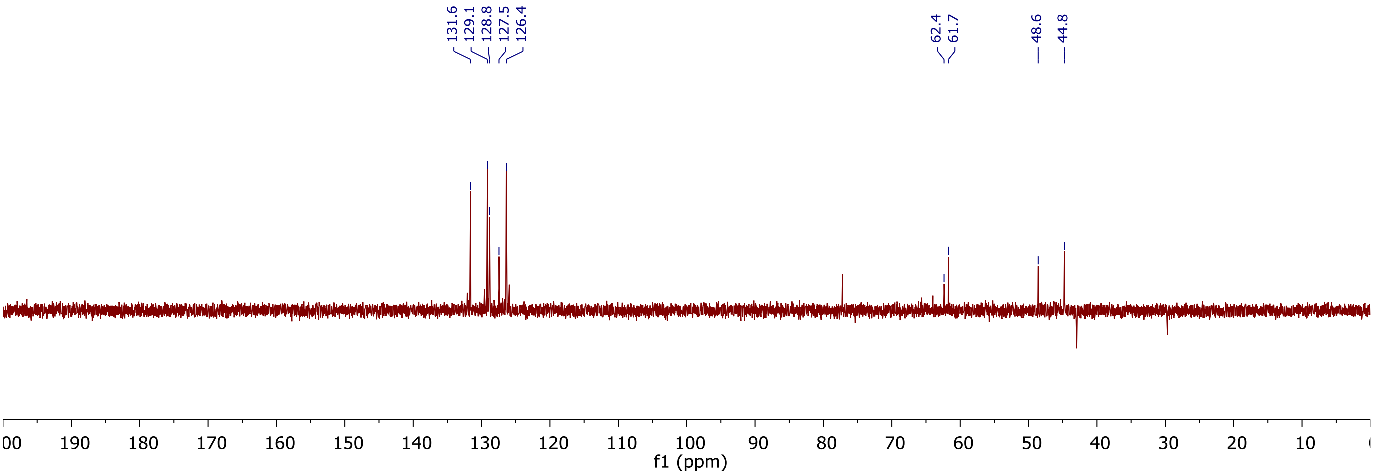

9 X-Ray diffraction structures

Compounds **3aa** and **3ai’** were recrystallized in Et_2O. Crystal growth was performed by slow evaporation at of the solvent.

9.1 X-Ray diffraction of 3aa (CCDC 2285841)

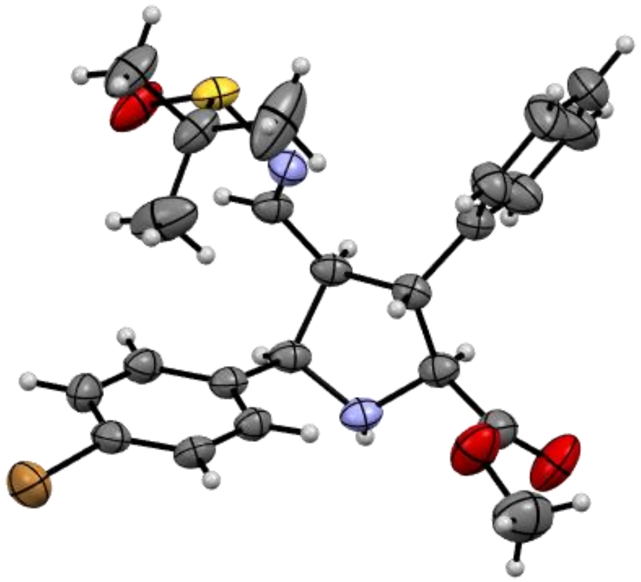

Figure S1. ORTEP diagram with thermal ellipsoids in 50% probability for **3aa**

CheckCIF/PLATON report for

```
Bond precision:    C-C = 0.0045 A           Wavelength=1.54184

Cell:               a=12.3738(2)     b=9.0664(1)         c=21.7605(4)
                    alpha=90         beta=106.230(2)     gamma=90
Temperature:        200 K

                   Calculated                  Reported
Volume             2343.93(7)                  2343.93(6)
Space group        I 2                         I 2
Hall group         I 2y                        I 2y
Moiety formula     C23 H27 Br N2 O3 S          C23 H27 Br N2 O3 S
Sum formula        C23 H27 Br N2 O3 S          C23 H27 Br N2 O3 S
Mr                 491.43                      491.44
Dx,g cm-3          1.393                       1.393
Z                  4                           4
Mu (mm-1)          3.432                       3.432
F000               1016.0                      1016.0
F000’              1017.01
h,k,lmax           15,11,26                    15,11,26
Nref               4653[ 2482]                 4571
Tmin,Tmax                                      0.833,1.000
Tmin’

Correction method= # Reported T Limits: Tmin=0.833 Tmax=1.000
AbsCorr = MULTI-SCAN

Data completeness= 1.84/0.98        Theta(max)= 72.540

                                                 wR2(reflections)=
R(reflections)= 0.0353( 4427)                    0.1028( 4571)
S = 1.008                    Npar= 279
```

9.2 X-Ray diffraction of 3ai' (CCDC 2292927)

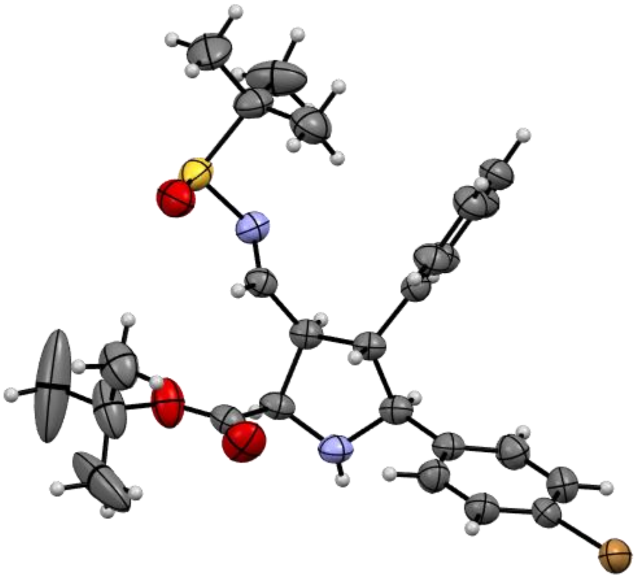

Figure S2. ORTEP diagram with thermal ellipsoids in 50% probability for **3ai'**

CheckCIF/PLATON report for

Bond precision:	C-C = 0.0084 A		Wavelength=1.54184
Cell:	a=22.6412(3)	b=22.6412(3)	c=10.8041(2)
	alpha=90	beta=90	gamma=120
Temperature:	200 K		

	Calculated	Reported
Volume	4796.43(15)	4796.43(13)
Space group	P 61	P 61
Hall group	P 61	P 61
Moiety formula	C26 H33 Br N2 O3 S	C26 H33 Br N2 O3 S
Sum formula	C26 H33 Br N2 O3 S	C26 H33 Br N2 O3 S
Mr	533.50	533.51
Dx,g cm-3	1.108	1.108
Z	6	6
Mu (mm-1)	2.550	0.000
F000	1668.0	1668.0
F000'	1669.82	
h,k,lmax	27,27,13	27,27,13
Nref	6257[3304]	5925
Tmin,Tmax		0.707,1.000
Tmin'		

Correction method= # Reported T Limits: Tmin=0.707 Tmax=1.000
AbsCorr = MULTI-SCAN

Data completeness= 1.79/0.95 Theta(max)= 71.720

R(reflections)= 0.0639(5526) wR2(reflections)= 0.1810(5925)

S = 1.059 Npar= 308

10 Experimental for DFT calculations

All the calculations reported in this work were performed within the Density Functional Theory (DFT) framework using the B3LYP hybrid functional[11] as implemented in the Gaussian 16[12] suite of programs. The standard 6-31G(d) basis set was used. Optimization of saddle points were performed in terms of ONIOM method implemented in Gaussian16 suite of programs. Atoms in high layer were represented in ball&stick. Transparent ball&stick atoms represent the ones included in the low layer. In the high-level layer, the electron correlation was taken into account by using the hybrid functional B3LYP3. In order to consider nonbonding interactions and dispersion forces, single-point calculations of the optimized ONIOM structures were carried out employing the Truhlar functional M06. All stationary points were characterized by harmonic analysis. Reactants, intermediates and cycloadducts have positive definite Hessian matrices.

Cartesian Coordinates for **TS*endo*^up^** (268.29 kcal·mol^{-1})

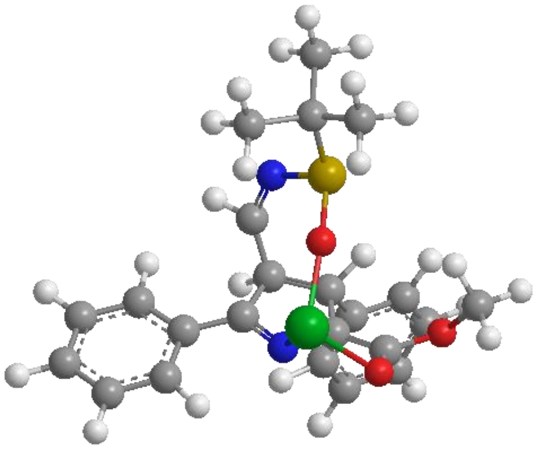

Cartesian Coordinates for **TSendoup** (Å)

N(1)	9.352	5.655	0.649
C(2)	9.127	5.608	-0.602
C(3)	10.141	5.417	-1.492
C(4)	8.317	6.110	1.231
C(5)	8.189	6.042	2.582
O(6)	9.019	5.314	3.137
C(7)	9.982	5.420	-2.834
C(8)	10.997	5.229	-3.691
C(9)	12.240	5.031	-3.235
C(10)	12.442	5.037	-1.912
C(11)	11.413	5.229	-1.071
O(12)	7.057	6.413	3.265
C(13)	6.488	5.436	4.106
Ag(14)	9.375	3.943	1.742
H(15)	6.386	5.961	0.760
H(16)	8.014	6.686	-1.685
H(17)	9.008	5.587	-3.315

[11] A. D. Becke *J. Chem. Phys.* **1993**, *98*, 5648–5650.
[12] Gaussian 16, Revision C.01, M. J. Frisch, G. W. Trucks, H. B. Schlegel, G. E. Scuseria, M. A. Robb, J. R. Cheeseman, G. Scalmani, V. Barone, G. A. Petersson, H. Nakatsuji, X. Li, M. Caricato, A. V. Marenich, J. Bloino, B. G. Janesko, R. Gomperts, B. Mennucci, H. P. Hratchian, J. V. Ortiz, A. F. Izmaylov, J. L. Sonnenberg, D. Williams-Young, F. Ding, F. Lipparini, F. Egidi, J. Goings, B. Peng, A. Petrone, T. Henderson, D. Ranasinghe, V. G. Zakrzewski, J. Gao, N. Rega, G. Zheng, W. Liang, M. Hada, M. Ehara, K. Toyota, R. Fukuda, J. Hasegawa, M. Ishida, T. Nakajima, Y. Honda, O. Kitao, H. Nakai, T. Vreven, K. Throssell, J. A. Montgomery, Jr., J. E. Peralta, F. Ogliaro, M. J. Bearpark, J. J. Heyd, E. N. Brothers, K. N. Kudin, V. N. Staroverov, T. A. Keith, R. Kobayashi, J. Normand, K. Raghavachari, A. P. Rendell, J. C. Burant, S. S. Iyengar, J. Tomasi, M. Cossi, J. M. Millam, M. Klene, C. Adamo, R. Cammi, J. W. Ochterski, R. L. Martin, K. Morokuma, O. Farkas, J. B. Foresman, and D. J. Fox, Gaussian, Inc., Wallingford CT, 2016.

H(18)	10.815	5.240	-4.780
H(19)	13.080	4.877	-3.933
H(20)	13.462	4.888	-1.517
H(21)	11.685	5.233	-0.004
H(22)	5.548	5.855	4.531
H(23)	6.240	4.526	3.515
H(24)	7.179	5.191	4.942
Lp(25)	9.909	5.797	0.822
Lp(26)	9.094	5.152	3.711
Lp(27)	7.274	6.836	3.632
Lp(28)	6.659	6.505	2.828
N(29)	6.430	3.993	-1.253
S(30)	6.152	3.259	0.254
O(31)	7.600	3.384	1.055
C(32)	7.160	4.995	-1.535
C(33)	7.824	5.970	-0.844
C(34)	7.323	6.398	0.351
C(35)	5.754	1.483	0.006
C(36)	7.089	7.741	0.485
C(37)	8.004	8.659	0.099
C(38)	7.794	9.979	0.226
C(39)	6.649	10.432	0.752
C(40)	5.722	9.548	1.147
C(41)	5.945	8.231	1.014
C(42)	5.603	0.811	1.384
C(43)	4.432	1.368	-0.775
C(44)	6.889	0.803	-0.783
H(46)	7.089	5.100	-2.641
H(49)	8.975	8.358	-0.329
H(50)	8.568	10.696	-0.097
H(51)	6.472	11.515	0.861
H(52)	4.775	9.909	1.583
H(53)	5.136	7.563	1.359
H(54)	5.347	-0.267	1.275
H(55)	6.544	0.867	1.977
H(56)	4.794	1.284	1.986
H(57)	4.152	0.301	-0.929
H(58)	4.504	1.836	-1.783
H(59)	3.590	1.857	-0.234
H(60)	7.027	1.262	-1.789
H(61)	7.862	0.866	-0.246
H(62)	6.670	-0.277	-0.942
Lp(63)	6.181	3.694	-1.711

Cartesian Coordinates for **TS*endo*$_{down}$** (271.07 kcal·mol^{-1})

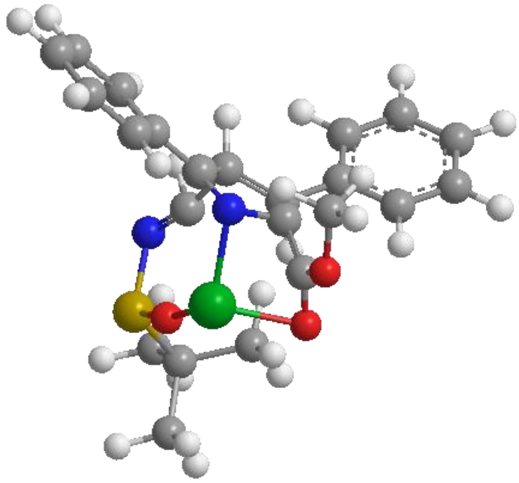

Cartesian Coordinates for **TS*endo*$_{down}$** (271.07 kcal·mol^{-1})

N(1)	0.709	-2.301	-2.344
C(2)	1.859	-1.842	-2.048
C(3)	2.615	-2.474	-1.110
C(4)	0.482	-1.928	-3.536
C(5)	-0.865	-2.107	-3.620
O(6)	-1.606	-1.105	-3.667
C(7)	3.850	-2.059	-0.755
C(8)	4.583	-2.690	0.177
C(9)	4.100	-3.773	0.799
C(10)	2.875	-4.206	0.476
C(11)	2.154	-3.564	-0.457
O(12)	-1.478	-3.359	-3.749
C(13)	-0.637	-4.485	-3.883
Ag(14)	-0.952	-1.270	-1.781
H(15)	0.571	-0.109	-3.958
H(16)	3.373	-1.042	-3.101
H(17)	4.325	-1.171	-1.199
H(18)	5.588	-2.315	0.439
H(19)	4.696	-4.293	1.566
H(20)	2.460	-5.095	0.984
H(21)	1.156	-3.990	-0.657
H(22)	-1.278	-5.364	-4.120
H(23)	-0.105	-4.698	-2.931
H(24)	0.080	-4.333	-4.720
Lp(25)	0.549	-2.838	-2.133
Lp(26)	-1.574	-1.280	-4.239
Lp(27)	-1.727	-3.320	-4.295
Lp(28)	-1.765	-3.444	-3.228
N(29)	2.105	1.409	-1.522
S(30)	0.584	1.900	-0.940
O(31)	-0.289	0.514	-1.230
C(32)	2.033	0.312	-2.165
C(33)	2.297	-0.834	-2.870
C(34)	1.348	-0.918	-3.860
C(35)	-0.061	3.264	-1.991
C(36)	1.676	-0.833	-5.186
C(37)	2.894	-0.424	-5.608
C(38)	3.224	-0.339	-6.907

C(39)	2.333	-0.660	-7.854
C(40)	1.111	-1.056	-7.476
C(41)	0.799	-1.133	-6.172
C(42)	0.869	4.484	-1.851
C(43)	-0.114	2.820	-3.466
C(44)	-1.478	3.631	-1.510
H(46)	3.144	0.534	-2.243
H(49)	3.686	-0.110	-4.911
H(50)	4.229	0.006	-7.203
H(51)	2.596	-0.589	-8.922
H(52)	0.361	-1.315	-8.242
H(53)	-0.230	-1.461	-5.955
H(54)	0.498	5.339	-2.460
H(55)	0.933	4.833	-0.795
H(56)	1.902	4.257	-2.198
H(57)	-0.495	3.641	-4.114
H(58)	-0.793	1.950	-3.613
H(59)	0.891	2.539	-3.853
H(60)	-2.179	2.770	-1.601
H(61)	-1.479	3.960	-0.447
H(62)	-1.901	4.464	-2.115
Lp(63)	2.590	1.750	-1.390
